# The Frq–Frh Complex Light-Dependently Delays Sfl1-Induced Microsclerotia Formation in *Verticillium dahliae*

**DOI:** 10.3390/jof9070725

**Published:** 2023-07-04

**Authors:** Alexandra Nagel, Miriam Leonard, Isabel Maurus, Jessica Starke, Kerstin Schmitt, Oliver Valerius, Rebekka Harting, Gerhard H. Braus

**Affiliations:** Department of Molecular Microbiology and Genetics, Institute of Microbiology and Genetics and Göttingen Center for Molecular Biosciences (GZMB), University of Göttingen, Grisebachstr. 8, D-37077 Göttingen, Germany

**Keywords:** *Verticillium dahliae*, plant pathogen, development, microsclerotia formation, clock gene homologs

## Abstract

The vascular plant pathogenic fungus *Verticillium dahliae* has to adapt to environmental changes outside and inside its host. *V. dahliae* harbors homologs of *Neurospora crassa* clock genes. The molecular functions and interactions of Frequency (Frq) and Frq-interacting RNA helicase (Frh) in controlling conidia or microsclerotia development were investigated in *V. dahliae* JR2. Fungal mutant strains carrying clock gene deletions, an *FRH* point mutation, or *GFP* gene fusions were analyzed on transcript, protein, and phenotypic levels as well as in pathogenicity assays on tomato plants. Our results support that the Frq–Frh complex is formed and that it promotes conidiation, but also that it suppresses and therefore delays *V. dahliae* microsclerotia formation in response to light. We investigated a possible link between the negative element Frq and positive regulator Suppressor of flocculation 1 (Sfl1) in microsclerotia formation to elucidate the regulatory molecular mechanism. Both Frq and Sfl1 are mainly present during the onset of microsclerotia formation with decreasing protein levels during further development. Induction of microsclerotia formation requires Sfl1 and can be delayed at early time points in the light through the Frq–Frh complex. Gaining further molecular knowledge on *V. dahliae* development will improve control of fungal growth and Verticillium wilt disease.

## 1. Introduction

Fungi can sense and adapt to changes in their environment [1,2]. A circadian clock allows for the anticipation and better preparation of rhythmic changes such as changing light and temperature conditions. This circadian clock system has been extensively studied using *Neurospora crassa* as reference organism [3]. In this fungus, the transcription factors White collar 1 (Wc1) and White collar 2 (Wc2) form the White collar complex (WCC), which is required for blue-light sensing and regulates expression of many light- and clock-regulated genes, including the circadian clock gene *FREQUENCY* (*FRQ*) [2,4,5,6,7]. The WCC induces *FRQ* transcription and subsequently alternative splicing leads to the production of two different Frq proteins, which are important for the fine-tuning of the clock [8,9,10,11,12]. Frq is an intrinsically disordered protein, meaning that it does not have a defined structure on its own and therefore requires interactions with other molecules. Homodimers of Frq associate with the highly structured Frq-interacting RNA helicase (Frh) to form the Frq–Frh complex [13,14,15,16]. Through this complex formation, Frh stabilizes Frq and regulates its cellular localization and interaction with the WCC [13,15,17,18]. In a negative feedback loop, the Frq–Frh complex promotes phosphorylation of the WCC, thereby inhibiting WCC activity and repressing *FRQ* transcription [19,20,21]. Additionally, the Frq–Frh complex mediates exosomal degradation of *FRQ* mRNA [22]. Frq itself becomes extensively phosphorylated and is eventually ubiquitinated. The latter is mediated by the F-box/WD-40 repeat-containing protein-1 (Fwd1) and results in degradation through the 26S proteasome [8,23,24,25,26,27,28]. This negative feedback loop allows for rhythmic expression of clock-regulated genes such as *FRQ* itself. A mutation in the essential *FRH* gene resulted in an amino acid (aa) exchange of Frh, namely, Frh^R806H^ [29]. This significantly weakened but still allowed Frq–Frh complex formation, whereas interaction of the Frq–Frh complex with the WCC was impaired and thus the negative feedback abolished [29,30].

Putative homologous genes encoding Wc1, Wc2, Frq, and Frh are widely distributed among the fungal clades and can be found in Ascomycota, Basidiomycota, and Mucoromycota [31,32]. Recently, the roles of circadian clock proteins in the insect-pathogenic ascomycetes *Beauveria bassiana* and *Metarhizium robertsii* have been investigated [33,34,35,36,37]. Notably, the *FRH* gene was not essential for cell viability in either of the entomopathogenic fungi. The respective clock gene homologs were found to have overlapping but also distinct or dispensable functions in important processes such as conidiation or virulence toward larvae of the wax moth *Galleria mellonella* [33,34,35,36,37]. Moreover, circadian clocks or clock gene homologs are not only relevant for plant immunity and defense responses against plant pathogens [38,39] but also for the development and virulence of phytopathogenic fungi [40,41,42,43,44,45,46]. MgWc-1 and MoFrq, as well as MoFwd1, which, as Fwd1 in *N. crassa*, modulates the degradation of MoFrq, control developmental processes and the virulence or disease severity of the rice blast fungus *Magnaporthe oryzae* (formerly *M. grisea*, anamorph: *Pyricularia oryzae*) [42,44,45]. In contrast, the Wc1 proteins of the soil-borne pathogens *Fusarium graminearum* and *Fusarium oxysporum* are dispensable for virulence on plants, although *F. oxysporum* Wc1 is required for full virulence on mammals [46,47]. Most fungi contain only one or two *FRQ* homologous gene copies, whereas even three genes were found in the *F. oxysporum* genome [48]. However, to date, the role of *FRQ* in *Fusarium* species has not been investigated [49]. In the necrotrophic plant pathogenic fungus *Botrytis cinerea*, the functions of BcWcl1 and BcFrq1, encoded by *WC1* and *FRQ* homologs, have been analyzed. BcWcl1 forms the WCC with BcWcl2 and mediates some transcriptional changes in response to light [41,50]. Further, BcWcl1 is involved in the response to oxidative stress, required for full virulence in light, and for suppression of conidiation [40,41]. The *FRQ* homolog of *B. cinerea* encodes BcFrq1, a functional circadian oscillator that modulates fungal virulence during *A. thaliana* infection. BcFrq1 was further found to be medium-dependently involved in the production of macroconidia and the repression of sclerotia formation in the light [40]. Altogether, this underlines the similar and yet distinct functions of core clock gene homologs in different fungi.

The fungus *Verticillium dahliae* is a broad-range plant pathogen in regions with temperate to subtropical temperatures that causes disease (“Verticillium wilt”) in diverse crops grown in these regions, such as olive trees, cotton, or tomato plants [51,52]. *V. dahliae* microsclerotia, which are its melanized resting structures, persist in the soil and ensure survival of the fungus for years until the next host plant can be infected [51,53,54]. Upon recognition of a nearby host, microsclerotia germinate and fungal hyphae can use the roots as entry points into the plant [51,55]. Once *V. dahliae* reaches the vasculature, it forms conidiospores, which serve to distribute the fungus within the xylem vessels of the host plant [56]. The fungus has to adapt to growth in xylem sap, which is poor and unbalanced in nutrients [57]. This adaptation has been studied using transcriptomics [58,59] and secretomics [60]. If conidia become stuck in different places in the plant xylem system, they germinate and proliferate in adjacent tissue. This is accompanied by the onset of disease symptoms [51,52]. Eventually, as the plant tissue becomes necrotic or senescent, *V. dahliae* forms microsclerotia as resting structures, which are released back into the soil [51]. This underlines the importance of both conidiospores for distribution through the xylem sap and microsclerotia for the ex planta survival of the fungus. Different transcription factors control the melanization, formation of microsclerotia, or conidiation in *V. dahliae* [58,61,62,63,64,65,66,67,68,69,70,71,72,73,74]. These include, among others, the Som1 and Vta3 transcription factors with shared and distinct regulatory networks. Downstream transcription factors of these regulatory networks include Vta1, Vta2, Aba1, Mtf1, and Suppressor of flocculation 1 (Sfl1) [58,61,67,68], of which Sfl1 was shown to be required for the formation of microsclerotia [61].

*V. dahliae,* like many other fungi, harbors clock gene homologs [31,48]. However, so far, no evidence for an endogenous circadian regulation in *V. dahliae* has been described [48]. *FRQ* plays a nutrient-dependent role in the vegetative growth of two different *V. dahliae* isolates from strawberry when external cues such as light or temperature are applied. However, *FRQ* was dispensable for rhythmic conidiation and microsclerotia formation. RNA-seq analyses suggested that *V. dahliae FRQ* controls the transcript levels of transcription factor-encoding genes as well as the transcript levels of genes involved in redox processes, transport, and metabolism, including several candidates for the biosynthesis of secondary metabolites. *FRQ* enhances infectivity and disease of a moderately but not of a highly virulent *V. dahliae* isolate [48].

The question of whether *FRQ* affects the amounts of conidia or microsclerotia produced by *V. dahliae* remains unresolved. Moreover, it is unknown whether the presence of a weakly conserved Frq–Frh interaction domain of *V. dahliae* Frq (4 out of 10 aa [48]) permits the formation of the Frq–Frh complex and whether Frh might affect protein amounts or localization of Frq. We addressed these questions genetically by comparing wild-type with mutant strains producing GFP fusion proteins or the Frh^R806H^ protein, *FRQ* deletion mutant strains, and for a more complete picture, *WC1* deletion strains. The tomato isolate *V. dahliae* JR2 [75] as wild-type background and the mutant strains were examined on transcript, protein, and phenotypic levels. Moreover, plant pathogenicity assays were conducted with these strains to explore whether there are connections to disease induction. This genetic comparative analysis revealed that the main function of the Frq–Frh complex in the analyzed *V. dahliae* strains is to slow down the Sfl1-mediated formation of microsclerotia as the resting structure during illumination.

## 2. Materials and Methods

### 2.1. Bioinformatic Methods

*V. dahliae* gene annotations were obtained from the Ensembl Fungi database [76], and protein sequences of other fungi used for comparisons were retrieved from FungiDB [77] or NCBI [78]. The InterPro website [79] was used for analysis of protein sequences, and alignments were performed using Clustal Omega [80,81]. Subcellular localization of proteins was predicted using DeepLoc 2.0 [82]. Statistical significances were calculated with independent two-sample *t*-tests using the iCalcu website (https://www.icalcu.com/stat/two-sample-t-test-calculator.html, accessed on 9 May 2023). Welch’s *t*-tests were used in case of unequal variance, and student’s *t*-tests were used on samples with equal variance as previously described [58]. In case of plant pathogenicity assay data, two-tailed Mann–Whitney *U* tests [83] were used (n.s.: not significant, *: *p* < 0.05, **: *p* < 0.01, ***: *p* < 0.001).

### 2.2. Cultivation of Microorganisms

*Escherichia coli* and *Agrobacterium tumefaciens* strains were cultivated in liquid or on solid lysogeny broth (LB) medium [84] according to previous descriptions [69,70]. *V. dahliae* cultures were incubated at 25 °C as previously described [60,68,70]. Conidia were harvested and concentrations determined as described [69] or using a Thoma counting chamber (Paul Marienfeld GmbH & Co. KG, Lauda-Königshofen, Germany).

### 2.3. Verification of Gene Annotation

The gene annotations of *V. dahliae* JR2 *FRQ* (*VDAG_JR2_Chr1g01960a*), *FRH* (*VDAG_JR2_Chr4g00070aa*), *WC1* (*VDAG_JR2_Chr2g01990a*), and *SFL1* (*VDAG_JR2_Chr4g02790a*), available at Ensembl Fungi database [76], were verified via the amplification and sequencing of the wild-type cDNA. Oligonucleotides ML60/ML61 were used for cDNA amplification of *FRQ*, AN67/AN64 for *FRH* cDNA, AN76/AN77 for amplification of *WC1* cDNA, and *SFL1* cDNA was amplified using RH664/RH665. Fragments were ligated into the pJET1.2/blunt cloning vector using the CloneJET PCR Cloning Kit (Thermo Fisher Scientific, Waltham, MA, USA) and sequenced (Microsynth Seqlab, Göttingen, Germany).

### 2.4. Plasmid and Verticillium Strain Construction

The general steps conducted for cloning and strain construction were previously described [59]. All primer oligonucleotides used in this study are listed in Table S1 and plasmids are listed in Table S2. Newly constructed plasmids were transformed into chemically competent *E. coli* DH5α (Invitrogen, Thermo Fisher Scientific) using the heat shock method [85,86]. Confirmed plasmids were transformed into *A. tumefaciens* AGL1 [87] as described [88]. *V. dahliae* mutant strains were generated via *A. tumefaciens*-mediated transformation (ATMT) based on the method described [89]. Plasmid and strain constructions are described in detail in Methods S1. All bacterial and fungal strains that were used or generated in this study are listed in Table S3.

### 2.5. Genomic DNA Extraction and Southern Hybridization

Constructed strains were verified through Southern hybridizations as depicted in Figures S1–S5. Mycelium for the extraction of genomic DNA (gDNA) was obtained according to the previous description [59]. The gDNA extraction protocol was modified from [90] according to [60]. Southern hybridization was performed as previously described [60,61] using either Amersham Hybond-N membrane (GE Healthcare) or Whatman Nytran N nylon membrane (Cytiva).

### 2.6. Cultivation of V. dahliae for Time Point Experiments

Fungal strains were inoculated either into 50 mL liquid simulated xylem medium (SXM) using 1 × 10^6^ spores or onto 30 mL SXM agar covered with a nylon membrane (Cytiva) using 1 × 10^6^ or 4 × 10^6^ spores. Cultures were incubated at 25 °C for two, four, and six days, respectively. Mycelia from SXM agar plates were scraped off the membrane, transferred to Miracloth filters, dried between paper towels, and frozen in liquid nitrogen. Mycelia from liquid cultures were collected in Miracloth filters and washed with 0.96% (*w*/*v*) NaCl before drying and freezing. Material of one to three cultures was combined for one biological replicate (*N* = 1). The experiment was performed three times (*N* = 3). Representative pictures of cultures are presented in Figure S6.

### 2.7. Western Experiments and Quantification of GFP Fusion Protein Levels

Proteins were extracted from ground mycelium. The strains were cultivated as described above for quantitative western experiments, or for three days in potato dextrose medium (PDM). B* buffer was used for extraction, and protein concentrations were determined as previously described [69]. The extracts were subjected to western experiments according to [60]. A total of 50 to 100 µg protein extracts were separated in non-gradient 8–10% SDS gels. Signals were visualized as described [69]. Detected signals were quantified, and Ponceau S staining was used for normalization as previously described [91]. Three independent biological replicates were conducted (*N* = 3). Images were processed afterwards using GNU Image Manipulation Program version 2.8.2 (https://www.gimp.org/).

### 2.8. RNA Extraction, cDNA Synthesis and Transcript Level Quantification

RNAs were extracted from ground *V. dahliae* mycelia using the TRIzol/chloroform protocol according to [70] with the following modifications: The mycelium was mixed with 1.3 mL TRIzol for 10 min and the RNA-containing aqueous phase that was obtained after addition of chloroform was mixed with 300 µL isopropanol and 300 µL high-salt buffer. Extracted RNAs were dried at 65 °C and then dissolved in 60–80 µL RNase-free water. Synthesis of cDNA was performed as described [59] and gDNA contamination was excluded via PCR with primer pair SZ19/SZ20 [68]. Quantitative reverse transcription PCR (qRT-PCR) was conducted using the CFX Connect Real Time PCR Detection System (Bio-Rad Laboratories) with MESA GREEN qPCR MasterMix Plus for SYBR Assay (Eurogentec) and primers listed in Table S4. Three independent experiments, each consisting of one biological replicate (*N* = 3), were conducted for transcript level analysis, with the exception of *CMR1* and *VTA1* transcript level analysis after two-day cultivation, for which four experiments with a total of six biological replicates (*N* = 6) were conducted. The qRT-PCRs were run for 30 cycles, or in the case of *VTA3*, for 35 cycles. Transcript levels were quantified relative to the reference genes histone *H2A* and *EIF2B* using the $2^{-∆∆CT}$ method [92]. Wild-type transcript levels after two days of incubation were set to one.

### 2.9. Phenotypical Analysis

Freshly harvested spores of the *V. dahliae* JR2 wild-type and respective mutant strains were point-inoculated onto Czapek-Dox medium (CDM), SXM, and PDM agar as previously described [68,70] and grown either in light or darkness for 10 to 14 days. For the analysis of colony melanization over time, 50,000 spores were point-inoculated onto CDM plates and incubated in the light for 24 days. The bottom view of the colonies was documented after 10, 14, 17, 21, and 24 days.

### 2.10. Quantification of Microsclerotia and Conidiospore Formation

Melanization was quantified as indication for microsclerotia formation as previously described [70]. Colony melanization was quantified after 19 to 14 days of incubation at 25 °C either in light or in darkness. Conidiospore formation was quantified after five days of incubation according to previous descriptions [69,70]. Two to four technical replicates were conducted per biological replicate (*N* = 1) for all experiments.

### 2.11. Pathogenicity Assay on Tomato Plants

Pathogenicity assays were conducted using *Solanum lycopersicum* (‘Moneymaker’, Kiepenkerl Bruno Nebelung). Surface sterilization of the seeds was performed as previously described [59,60]. Ten-day-old seedlings were infected via root dipping in 50 mL of a 1 × 10^7^ spores per ml suspension, grown for 21 days under long-day conditions and evaluated according to the protocol described previously [60,68]. Briefly, plant height, weight, and longest leaf length were measured and compared with the means of water-inoculated (mock) plants, each set as 100%. Each parameter was characterized as either healthy (≥80%; (1)) or as a mild (60–79%; (2)), strong (40–59%; (3)) or very strong symptom (≤39%; (4)). The mean disease level of the three parameters determined the disease score of the individual plant. Plants with a mean disease level of 1–1.99, 2–2.99, 3–3.99 or 4 were rated as healthy, as having weak symptoms, as having strong symptoms, or as having very strong symptoms, respectively. Stacking diagrams show the relative number of plants assigned to the respective symptom category. Hypocotyl discoloration was observed by binocular microscopy (SZX12-ILLB2-200, illuminated with KL1500 LCD, Olympus).

### 2.12. Confocal Microscopy

Subcellular localizations of GFP fusion proteins were examined via fluorescence microscopy as described [69]. Briefly, approximately 1 × 10^4^ to 1 × 10^6^ spores were inoculated into 300 µL PDM and grown overnight at 25 °C in the light. Either a 100×/1.4 oil objective (Plan-Apochromat) or 63×/0.75 air objective (Plan-Neofluar) were used with the Axio Observer Z1 system (Zeiss) with Laser Lunch System (Model 3iL32, Intelligent Imaging Innovations), QuantEM:512SC camera (Photometrics), and Slide Book 6.0 imaging software (Intelligent Imaging Innovations).

Nuclei were visualized either through ectopically expressed *RFP–H2B* fusion constructs or staining with 4′,6-diamidino-2-phenylindole (DAPI, Carl Roth GmbH + Co. KG, Karlsruhe, Germany).

### 2.13. In Vitro Protein Pull-Down and LC/MS Analyses

As many as 5 × 10^9^ freshly harvested spores of an *FRH–GFP*-expressing strain, *FRH^R806H^–GFP*-expressing strain, ectopically *GFP*-overexpressing strain, or *V. dahliae* JR2 wild-type strain were used to inoculate 500 mL SXM. After two days of incubation in light with shaking at 25 °C, mycelia from one to three cultures each were combined (*N* = 1) and ground in liquid nitrogen. Ground mycelia of *V. dahliae* wild-type and the *GFP*-overexpressing strain VGB45 were mixed to obtain approximately the same level of free GFP in the wild-type control compared with Frh–GFP fusion protein (Figure S7; 59/60 wild-type, 1/60 VGB45). Protein extraction with B*-buffer, the in vitro protein pull-down, subsequent chloroform–methanol extraction, protein digestion with trypsin in the presence of *Rapi*Gest SF (Waters), and subsequent TFA treatment were conducted for three biological replicates (*N* = 3) as previously described [70] with the following modification: the three eluates of the protein pull-down were mixed. Peptides were purified with StageTips [93,94] according to the described protocol [58]. Purified peptides were solved and liquid chromatography/mass spectrometry (LC/MS) analysis was conducted as previously described [70]. Raw MS data were analyzed and processed using MaxQuant 1.6.10.43 [95] and Perseus 1.6.0.7 [96] according to the described protocol [70]. Rows were filtered for a minimum of three valid values in samples of either the *FRH–GFP* or the *FRH^R806H^–GFP*-expressing strain. The replacement of missing values from normal distribution was independently conducted four times. The fusion protein-expressing strain (*FRH–GFP* or *FRH^R806H^–GFP*) was used as the first group in the volcano plot and the control as the second group. The mass spectrometry proteomics data have been deposited to the ProteomeXchange Consortium via the PRIDE [97] partner repository with the dataset identifier PXD041716.

## 3. Results

### 3.1. Light-Dependent Repression of Microsclerotia Formation and Induction of Aerial Hyphae Formation in V. dahliae Require FRQ, FRH, and WC1

Homologs of the key circadian clock genes known from *Neurospora crassa*, including *FRQ*, *FRH*, *WC1*, and *WC2*, are present in *V. dahliae* [48], but no circadian rhythmicity in *V. dahliae* has yet been described. The *V. dahliae* genomic loci and deduced protein structures of the corresponding clock gene homologs that were used for this study (*FRQ*: *VDAG_JR2_Chr1g01960a*, *FRH*: *VDAG_JR2_Chr4g00070aa*, *WC1*: *VDAG_JR2_Chr2g01990a*) are depicted in Figure S8. The 1175 aa protein sequence of *V. dahliae* JR2 Wc1 is 63 aa longer than the previously depicted and analyzed Wc1 sequence [48]. *V. dahliae* JR2 Wc1 shares 58.16% identity with *N. crassa* Wc-1 (NCU02356). An alignment of both protein sequences is depicted in Figure S9.

*V. dahliae FRQ* is dispensable for rhythmic conidiation or microsclerotia formation under oscillating light or temperature conditions, but a corresponding deletion affects metabolic and redox processes [48]. The impact of a *FRQ* deletion on *V. dahliae* development itself including the efficiency to produce conidia or microsclerotia is yet elusive and was addressed for *FRQ* as well as the clock gene homologs *FRH* and *WC1* of the tomato isolate *V. dahliae* JR2. As resting structures, melanized microsclerotia are the start and the end point of the plant infection cycle of *V. dahliae* [51]. Therefore, we examined whether clock gene homologs are required for microsclerotia formation. Strains carrying a *WC1* deletion (∆*WC1*), *FRQ* deletion (∆*FRQ*), or constructs for Frq–GFP fusion protein production were constructed (Figures S1–S3). The *V. dahliae FRH* gene was modified by a single point mutation (Figure S4) because it exhibits not only clock but also additional essential functions in *N. crassa* [15]. A point mutation from guanine to adenine at position 2481 leads to an arginine to histidine codon exchange (*FRH^R806H^*) and is known to permit these essential functions; however, it interrupts clock functions of Frh, leading to increased *FRQ* transcript levels [29].

**Figure 1:**
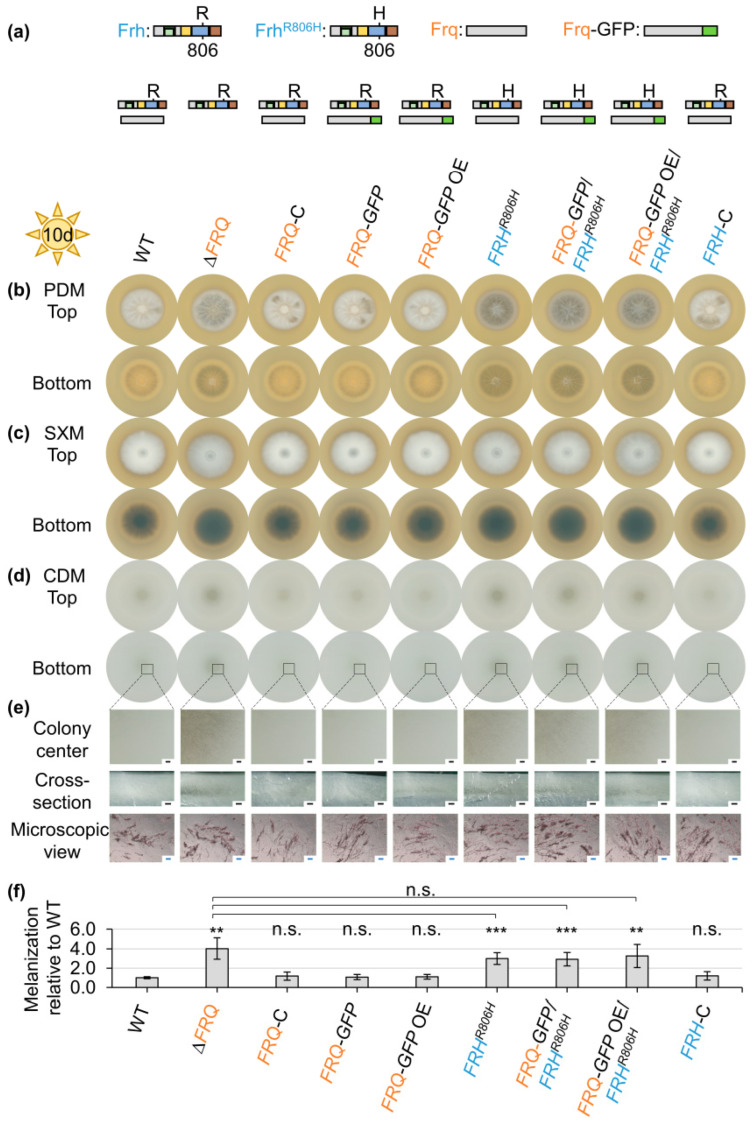
*V. dahliae* Frequency (Frq) and Frq-interacting RNA helicase (Frh) repress microsclerotia formation and induce aerial hyphae production. (**a**) Schematic depiction of Frq and Frh proteins present in respective strains. (**b**–**f**) The ex planta phenotype was analyzed of *V. dahliae* wild-type (WT), a *FRQ* deletion strain (∆*FRQ*), strains expressing *FRQ–GFP* under native- (*FRQ–GFP*) or *gpdA*-promoter control (*FRQ–GFP* OE), strains with a *FRH* point mutation in wild-type (*FRH^R806H^*) or mutant strain background (*FRQ–GFP*/*FRH^R806H^*, *FRQ–GFP* OE/*FRH^R806H^*), as well as respective complementation strains (*FRQ*-C, *FRH*-C). 50,000 spores were point-inoculated onto (**b**) potato dextrose medium (PDM), (**c**) simulated xylem medium (SXM), and (**d**–**f**) Czapek-Dox medium (CDM) agar and incubated at 25 °C in the light for ten days. Deletion of *FRQ* and point mutation of *FRH* resulted in darker, stronger melanized colonies, suggesting enhanced microsclerotia formation compared with wild-type. (**e**) Representative pictures of the colony center after removal of aerial hyphae, cross-sections, and microscopic images of microsclerotia of CDM cultures are depicted. Black scale bar: 500 µm; blue scale bar: 50 µm. (**f**) Colony melanization was quantified on CDM after ten days. The experiment was performed three times with two independent transformants of each mutant strain and one to three independent WT cultures (*N* = 6). Depicted is the mean of six biological replicates with standard deviation. WT melanization was set as one. Statistical differences were calculated using *t*-tests (n.s.: not significant, **: *p* < 0.01, ***: *p* < 0.001). Differences compared with WT are indicated on top of the bars and non-significant differences between *FRQ* deletion and *FRH* point mutation strains are shown by the connecting lines. Deletion of *FRQ* and *FRH* point mutation in all different backgrounds led to significantly increased colony melanization.

Spores of the wild-type, *FRQ*, and *FRH* mutants, as well as complementation strains, were point-inoculated onto a glucose-rich medium (PDM), a pectin-rich medium (SXM), and a minimal medium (CDM). The ex planta phenotypes of the colonies were analyzed after ten days (Figure 1). *FRQ* deletion and *FRH^R806H^* point mutation strains formed darker colonies on all media tested (Figure 1b–d). This was best visible on the bottom of the colony due to the production of aerial hyphae at the agar surface. Colony centers of CDM cultures were further investigated through binocular and light microscopy (Figure 1e). The observed intensification in colony pigmentation resulted from increased production of melanized microsclerotia compared with wild-type. *FRQ* deletion and *FRH^R806H^* point mutation resulted in an earlier onset of microsclerotia development as colonies of the mutant strains continuously melanized faster than those of the wild-type (Figure S10). Reintroduction of *FRQ* or introduction of the *FRQ–GFP* construct into the *FRQ* deletion strain (Figure S2) and reintroduction of wild-type *FRH* into the *FRH^R806H^* mutant strain (Figure S4) complemented the deletion or mutation phenotype, respectively. Notably, not only the expression of the *FRQ–GFP* construct under control of the endogenous promoter (*FRQ–GFP*) but also the *gpdA* promoter-controlled expression of *FRQ–GFP* (*FRQ–GFP* OE) allowed for growth similar to wild-type (Figure 1). Quantification of *FRQ–GFP* transcript levels in the presence of either wild-type or point-mutated *FRH* revealed that *gpdA* promoter-mediated expression did not result in elevated transcript levels. The Frq–GFP protein level was even reduced after two-day cultivation on SXM agar (Figure S11). Melanization of colonies grown on CDM was quantified as indication for microsclerotia formation (Figure 1f). Colony melanization was significantly increased through deletion of *FRQ* (~300% increased) and *FRH^R806H^* point mutation in either wild-type, *FRQ–GFP*, or *FRQ–GFP* OE background (~200% increased) compared with wild-type. The increase in microsclerotia formation due to *FRH^R806H^* point mutation does not significantly differ from the *FRQ* deletion effect. The melanization level of complementation and *FRQ–GFP*-expressing strains with intact *FRH* was similar to the wild-type. However, when incubated in the dark, colonies of the wild-type and strains with either *FRQ* deletion or Frh^R806H^ aa substitution were melanized to a similar extent (Figure S12). These results demonstrate that *FRQ* and *FRH* are both required for repression of microsclerotia formation specifically when grown in the light. In addition to this repressive effect of Frq and Frh on microsclerotia formation, both proteins also positively affect aerial hyphae formation. This is especially visible when the fungus is grown on PDM plates, but it is also observable for colonies grown on SXM plates (Figure 1b,c). There, *FRQ* deletion and *FRH^R806H^* mutation result in reduced aerial hyphae growth.

**Figure 2:**
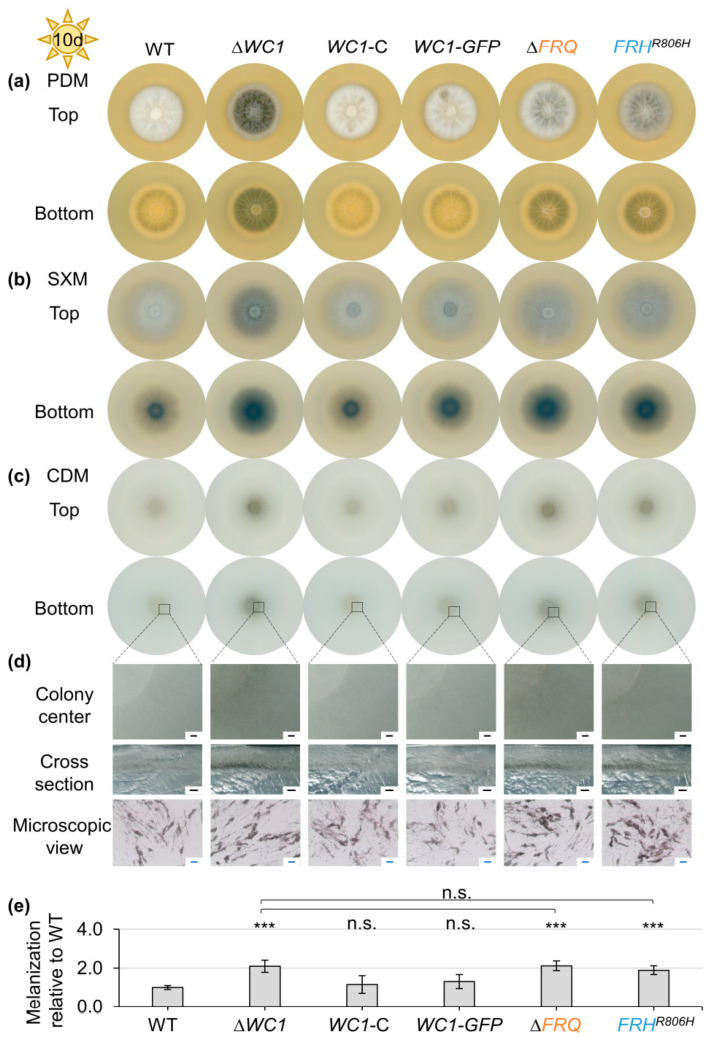
*V. dahliae* White collar 1 (Wc1) is required for repression of microsclerotia formation and induction of aerial hyphae production. A total of 50,000 spores of the *V. dahliae* wild-type (WT), *WC1* deletion (∆*WC1*), complementation (*WC1*-C), and WC1–GFP fusion protein producing (*WC1–GFP*) strains, as well as the *FRQ* deletion (∆*FRQ)* and *FRH* point mutation (*FRH^R806H^*) strains, were point-inoculated onto (**a**) PDM, (**b**) SXM, and (**c**–**e**) CDM. The phenotype was investigated after incubation in the light for ten days. Similar to the *FRQ* deletion and *FRH^R806H^* point mutation, *WC1* deletion resulted in increased colony melanization. (**d**) Depicted are representative pictures of the colony center after removal of aerial hyphae, cross-sections through the colony center, and microscopic images of microsclerotia of CDM-grown colonies. Black scale bar: 500 µm, blue scale bar: 50 µm. (**e**) The melanization of the colony center was quantified relative to WT (set as one) after incubation on CDM in the light for ten days. The experiment was performed three times with two independent mutant strain transformants or independent WT cultures (*N* = 6). Only one *WC1–GFP*-expressing transformant was analyzed (*N* = 3). Depicted is the mean of biological replicates with standard deviation. Statistical differences were calculated using *t*-tests (n.s.: not significant, ***: *p* < 0.001). Differences compared with WT are indicated above the bars and the result from statistical comparison between *WC1* deletion and *FRQ* deletion or *FRH* point mutation strains are shown by the connecting lines. Similar to *FRQ* deletion and *FRH^R806H^* strain colonies, the ∆*WC1* strain colony was stronger melanized than the WT colony. *FRQ*, *FRH*, and *WC1* are similarly involved in repression of microsclerotia formation.

*FRQ* expression in *N. crassa* is mostly dependent on the presence of an intact *WC1* gene, but also *WC*-independent expression of *FRQ* was found [98,99,100,101,102,103]. Analysis of *FRQ* transcript levels in a *WC1* deletion mutant under different conditions reveals that also in *V. dahliae*, *FRQ* expression partially depends on *WC1* (Figure S11). The ex planta phenotype of the *WC1* deletion strain was analyzed in comparison with wild-type, *FRQ* deletion, and *FRH^R806H^* mutation as well as complementation strains. In the latter strains, either the *WC1* gene or a *WC1–GFP* fusion construct replaced the *WC1* deletion (Figures S3 and S12). Spores of these strains were point-inoculated onto different media and the colony appearance was analyzed after cultivation for ten days as described before (Figure 2). *WC1* deletion, similar to *FRQ* deletion and *FRH^R806H^* mutation, led to enhanced colony melanization on all media tested when cultivated in the light (Figure 2a–d), which was quantified for colonies grown on CDM (~100% increase compared with wild-type, Figure 2e). In contrast, deletion of *WC1* resulted in slightly but significantly reduced melanization (~9.6% reduced) when cultivated in darkness (Figure S12). Complementation through reintegration of *WC1* or *WC1–GFP* into the *WC1* deletion strain allowed for growth similar to wild-type. Like *FRQ* and *FRH*, *WC1* is required for wild-type-like growth of aerial hyphae. However, the role of Wc1 in positive control of aerial hyphae formation appeared stronger than that of Frq and Frh (Figure 2a,b). The ex planta phenotypes of ∆*WC1*/∆*FRQ* and ∆*WC1*/*FRH^R806H^* double mutant strains were indistinguishable from that of the *WC1* single-deletion mutant strain (Figure S13). Taken together, aerial hyphae formation in the light depends stronger on the presence of *WC1* than *FRQ* or *FRH*, but all three are required for light-dependent repression of microsclerotia formation. *WC1* is also partially required for induction of microsclerotia formation in darkness.

### 3.2. V. dahliae FRQ and FRH Positively Affect Conidiation

Since *WC1*, *FRQ,* and *FRH* are required for microsclerotia formation, potential additional contributions to the developmental process of conidiation were investigated. Once *V. dahliae* reaches the xylem vessels, it forms conidiospores for fast proliferation in the vasculature [51,52,56]. Thus, conidiospore formation is important for effective fungal plant colonization. Spores of the previously described *FRQ*, *FRH,* and *WC1* mutant strains were inoculated into liquid SXM, a pectin-rich medium favoring *V. dahliae* conidiation. After five days of incubation, conidia were quantified relative to wild-type control set as one.

**Figure 3:**
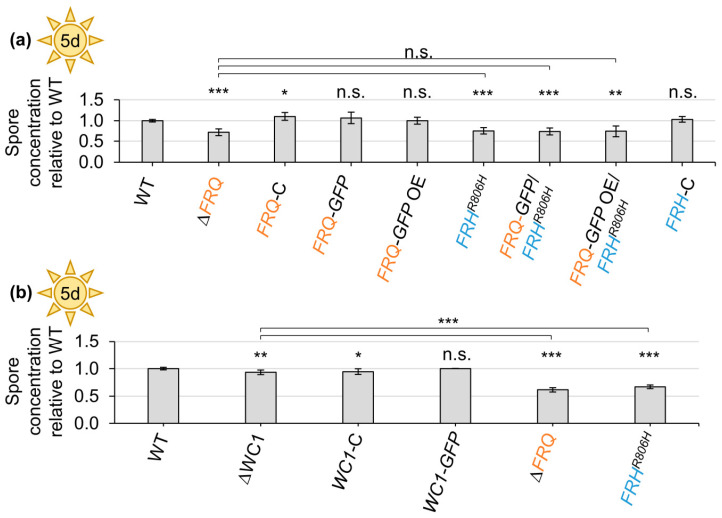
*V. dahliae* Frq and wild-type Frh positively affect spore production. A total of 200,000 spores of the *V. dahliae* wild-type (WT), *FRQ* deletion (∆*FRQ*), Frq–GFP fusion protein producing (*FRQ–GFP; FRQ–GFP* OE), *FRH^R806H^* point mutation (*FRH^R806H^*, *FRQ–GFP*/*FRH^R806H^*, *FRQ–GFP* OE/*FRH^R806H^*), *WC1* deletion (∆*WC1*), *WC1–GFP*-expressing (*WC1–GFP*), and respective complementation strains (*FRQ*-C, *FRH*-C, *WC1*-C) were inoculated into 50 mL liquid SXM and incubated under agitation at 25 °C in the light. The number of produced spores was quantified after five days. The experiment was performed three times with two transformants of the mutant strains and independent wild-type cultures (*N* = 6), except for *WC1–GFP*, of which only one transformant was used (*N* = 3). Bars represent the mean of biological replicates with standard deviation. WT spore production was set as one. Statistical differences were calculated using *t*-tests (n.s.: not significant, *: *p* < 0.05, **: *p* < 0.01, ***: *p* < 0.001). Results of statistical analyses compared with the WT are displayed above the bars, and other comparisons are indicated with the connecting lines. (**a**) Significantly fewer conidia were produced by *FRQ* deletion and *FRH* point mutation strains compared with WT. (**b**) *WC1* deletion also resulted in significantly reduced conidiation compared with WT. However, the positive effect on conidiation mediated by *WC1* was significantly smaller than that mediated by *FRQ* and *FRH*.

*FRQ* deletion or the Frh^R806H^ aa substitution in all different backgrounds led to significantly decreased conidia production (~25–29% reduced) compared with wild-type (Figure 3a). Reintegration of *FRQ* or *FRQ–GFP* under endogenous or *gpdA*-promoter control, as well as *FRH* reintegration, allowed for conidiation similar to wild-type. Deletion of *WC1* resulted in a small but significant decrease in produced conidia (~7% reduced). However, the positive effect of Wc1 on conidiation was significantly lower than the *FRQ*- and *FRH*-mediated induction (~33–39%) of conidia production (Figure 3b). Thus, Frq and Frh are required for induction of conidiation, whereas Wc1 plays only a minor role in this developmental process.

### 3.3. Symptom Induction in V. dahliae Infected Tomato Plants Is Independent of FRQ, FRH, and WC1

**Figure 4:**
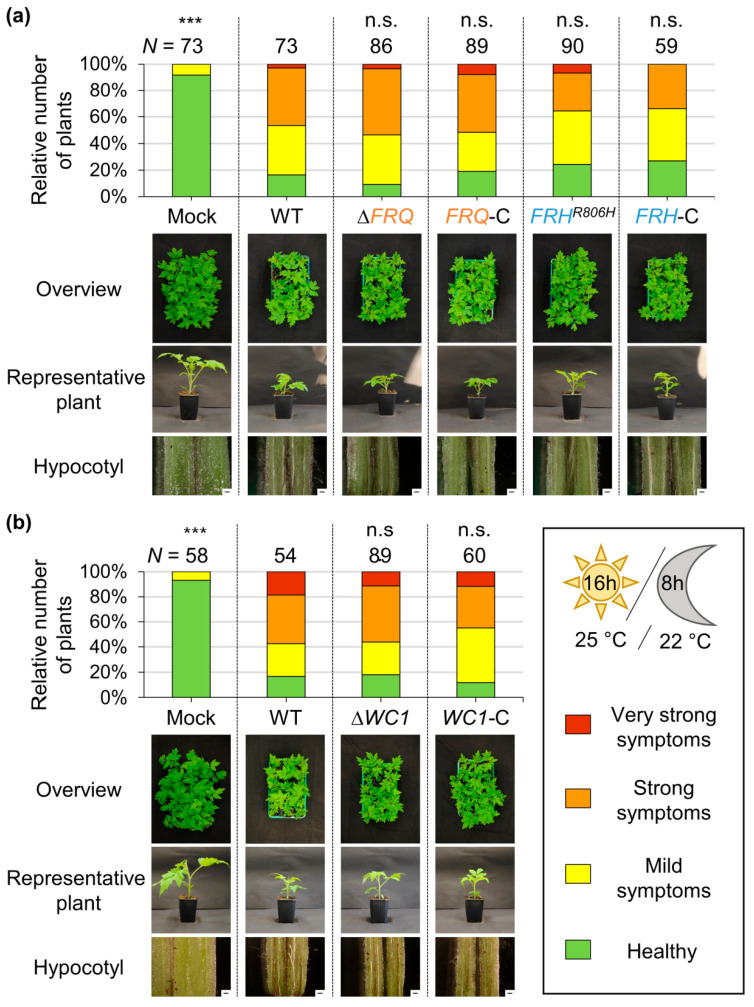
Clock gene homologs, *FRQ*, Frh amino acid residue arginine 806, and *WC1* are dispensable for *V. dahliae*-mediated symptom induction in tomato plants. Ten-day-old tomato plant seedlings were wounded and infected with spores of *V. dahliae* wild-type (WT), (**a**) *FRQ* deletion (∆*FRQ*), *FRH^R806H^* point mutation (*FRH^R806H^*), or their respective complementation strains (*FRQ*-C, *FRH*-C), as well as (**b**) *WC1* deletion (∆*WC1*) or complementation (*WC1*-C) strains. Two independent transformants of mutant strains served as biological replicates. Control plants were inoculated with water (mock). After incubation under long-day-conditions for 21 days, plants were classified as being healthy (mean disease level = 1–1.99; green), or showing mild (mean disease level = 2–2.99; yellow), strong (mean disease level = 3–3.99; orange), or very strong (mean disease level = 4; red) symptoms based on the height of the plant, the length of the longest leaf, and the fresh weight. Depicted are results from (**a**) three or (**b**) two independent experiments with 11 to 15 plants per biological replicate. The number of total plants (*N*) is indicated above the bars. Statistical significances compared with WT infection were calculated with Mann–Whitney *U* tests (n.s: not significant, ***: *p* < 0.001). Pictures of plant trays, representative individual plants, and hypocotyl cross sections are depicted below the diagram. *V. dahliae FRQ*, Frh amino acid residue arginine 806, and *WC1* were dispensable for wild-type-like symptom development in tomato plants.

In *V. dahliae* strawberry isolates, the presence of *FRQ* is important for disease induction. This effect is isolate dependent and was not found for disease induction of highly virulent isolates [48]. We previously observed a role of the clock gene homologs in microsclerotia formation and conidiation, with the latter being important for host plant colonization. Thus, we analyzed if these genes are also important for disease induction in the tomato isolate *V. dahliae* JR2. Tomato seedlings were infected with spores of wild-type, clock gene mutant strains, and their respective complementation strains or inoculated with water (mock). Plant height, leaf length, and plant fresh weight were measured after 21 days to characterize plants as being healthy, or showing mild, strong, or very strong symptoms compared with mock plants [60,68]. The percentage of plants belonging to the individual categories is displayed in stack diagrams (Figure 4). The spore-treated plants were stunted compared with mock-treated plants and had brownish discolored hypocotyls. We found no significant difference between disease symptoms induced by strains with *FRQ* or *WC1* deletion, Frh^R806H^ aa exchange, or respective complementation strains compared with wild-type. Thus, *V. dahliae* JR2 *FRQ*, *FRH^R806^*, and *WC1* were dispensable for disease induction in its host plant tomato.

### 3.4. The Frq–Frh Complex Formation in V. dahliae Depends on Frh Amino Acid Residue Arginine 806

**Figure 5:**
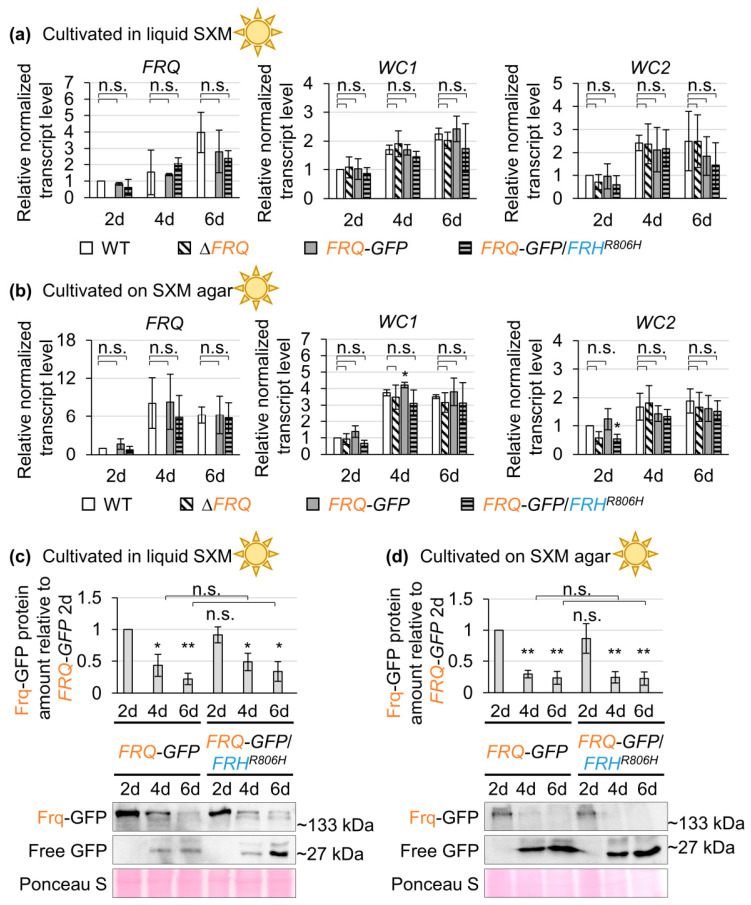
*V. dahliae* Frh amino acid residue arginine 806 is largely dispensable for *FRQ*, *WC1*, and *WHITE COLLAR 2* (*WC2*) transcript and Frq protein levels. As many as 1 × 10^6^ spores of the *V. dahliae* wild-type (WT), *FRQ* deletion strain (∆*FRQ*) as well as *FRQ–GFP*-expressing strains with either wild-type *FRH* (*FRQ–GFP*) or point mutated *FRH* (*FRQ–GFP*/*FRH^R806H^*) were grown in (**a**,**c**) liquid SXM or (**b**,**d**) on SXM agar covered with a nylon membrane. Cultures were incubated at 25 °C in the light for two (2d), four (4d), and six days (6d) before (**a**,**b**) RNAs or (**c**,**d**) proteins were extracted. (**a**,**b**) Transcript levels of *FRQ, WC1*, and *WC2* were investigated via quantitative reverse transcription PCR for indicated strains and time points. Depicted is the mean of three biological replicates with standard deviation (*N* = 3). Reference genes *H2A* and *EIF2B* were used for normalization and the two-day WT transcript levels were set as one. Significant differences to WT samples of respective time points are labeled above the bar. Non-significant differences are indicated by connecting lines (calculated with *t*-tests, n.s.: not significant, *: *p* < 0.05). Differences in *FRQ* transcript levels between WT and the ∆*FRQ* strain could not be statistically analyzed as no FRQ transcript was detected in the latter strain. *FRQ* transcript levels were not significantly changed upon Frh^R806H^ amino acid exchange. The *WC2* transcript level was only significantly decreased upon *FRH* mutation during cultivation on SXM agar for two days. (**c**,**d**) The protein amount of Frq–GFP was quantified using a GFP antibody in western experiments. Images of representative replicates are depicted below the graphs. The pixel density of signals was normalized to the Ponceau S staining. The protein signal intensity of the *FRQ–GFP* strain after two days of growth was set as one. The mean of three biological replicates with standard deviation is depicted (*N* = 3). Statistical significances were determined with *t*-tests (n.s.: not significant, *: *p* < 0.05, **: *p* < 0.01). Differences to *FRQ–GFP* after two days are indicated above the bars. As shown by the connecting lines, no significant difference was observed between four-day or six-day Frq–GFP protein levels. The amino acid exchange in the Frh protein did not significantly affect the Frq–GFP protein levels.

Previous research in *N. crassa* suggested that the *FRH^R806H^* mutation breaks the negative feedback loop and therefore causes *FRQ* overexpression [29]. In *V. dahliae*, however, codon exchange for the respective residue (Frh^R806H^) leads to the same developmental phenotypes as the *FRQ* deletion (Figure 1 and Figure 3). Even *gpdA* promoter-mediated *FRQ* expression in the presence of the mutated *FRH* did not result in elevated Frq levels, but cultivation on SXM agar for two days even led to significantly reduced Frq–GFP levels (Figure S11). Thus, we further investigated how the *FRH^R806H^* point mutation affects endogenously expressed *FRQ* transcript and protein levels. RNAs and proteins were extracted from cultures grown under conditions favoring either conidiation (in liquid SXM) or microsclerotia formation (on SXM agar). Samples were harvested after two, four, and six days (Figure S6). Relative normalized transcript levels of *FRQ*, *WC1*, and *WC2* are depicted in Figure 5a,b. Wild-type expression after two days was set as one. No *FRQ* transcript was detected in the *FRQ* deletion strain; therefore, in this case no significance of differences were calculated. Neither expression of *WC1* nor *WC2* differed significantly between wild-type, *FRQ* deletion strain, or the *FRQ–GFP*-expressing strain with or without aa substitution in Frh during cultivation under conidiation favoring conditions (Figure 5a). The transcript level of *FRQ* was not significantly affected by *FRH^R806H^* point mutation in either of the two cultivation conditions (Figure 5a,b). When the strains were cultivated under microsclerotia favoring conditions, *WC1* expression was slightly elevated in the *FRQ–GFP*-expressing strain (~12% increased) compared with wild-type after four days of cultivation (Figure 5b). The transcript level of *WC2* was only significantly decreased upon *FRH* point mutation (~46% decreased) after two days of cultivation on SXM agar. Taken together, the Frh^R806H^ aa exchange did not affect *FRQ* transcript levels and only led to a reduction in *WC2* transcript levels after two days.

As the transcript level does not necessarily reflect the protein level, we next investigated if the Frh^R806H^ aa substitution influences the protein abundance of Frq. Frq–GFP protein levels were quantified via western experiments using a GFP antibody. Proteins were extracted after two, four, and six days of incubation (Figure S6). The level of Frq–GFP was compared between the *FRQ–GFP*-expressing strains in wild-type *FRH* and *FRH^R806H^* background. Frq–GFP has a predicted molecular weight of ~133 kDa but fusion protein signals slightly above 180 kDa were also detected. This difference likely originates from post-translational modification of Frq, which is known to be extensively phosphorylated in *N. crassa* [8], with more than 100 identified phosphorylation sites [25,26,27,28]. Of these phosphorylation sites, 51 seem to be conserved in *V. dahliae* Frq (Figure S9). For quantification of Frq–GFP levels, signals ≥ 133 kDa were measured. In general, the Frq–GFP protein level decreased after two days of cultivation, and free GFP (~27 kDa) was detected as a degradation product after four and six days of cultivation (Figure 5c,d). A direct comparison of the Frq–GFP levels after two days of cultivation in liquid SXM or on SXM agar revealed that the growth conditions and presumably the developmental program which is mainly induced by the respective environment influenced Frq protein levels (Figure S14). When cultured under microsclerotia-promoting conditions (on SXM agar), Frq–GFP levels were significantly reduced in comparison with cultivation in an environment promoting conidiation (in liquid SXM). Frq–GFP was also present when cultures were grown in the dark, and the protein level did not significantly differ from light-incubated cultures (Figure S14). These results indicate that the protein level of *V. dahliae* Frq does not oscillate in a circadian manner but depends on nutrition and fungal development. The *FRH^R806H^* point mutation could not even affect Frq–GFP protein levels of natively expressed *FRQ–GFP* under any of the tested cultivation conditions (Figure 5c,d).

**Figure 6:**
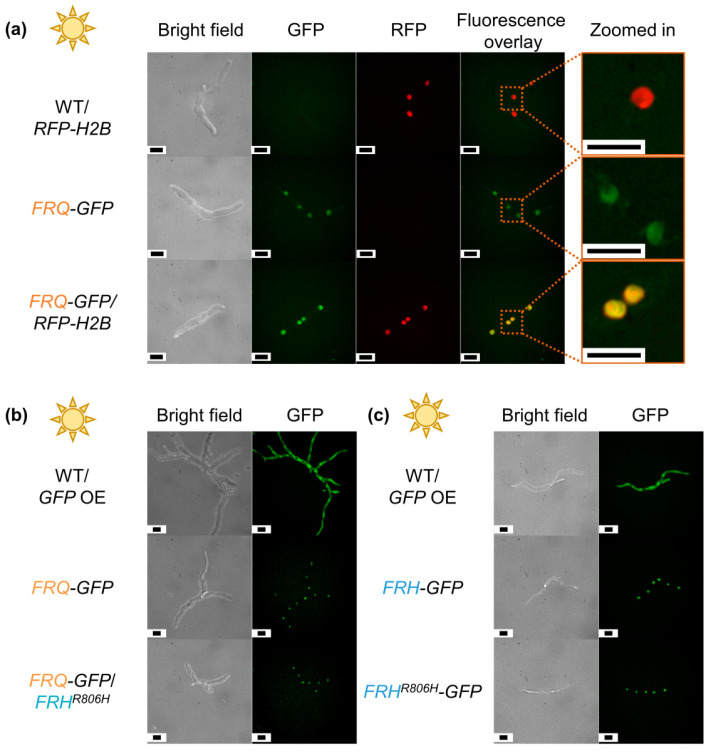
Nuclear localization of Frq–GFP and Frh–GFP is unaffected by Frh amino acid substitution. For the analysis of subcellular localization of Frq–GFP, Frh–GFP, or Frh^R806H^–GFP fusion proteins, spores of the indicated strains were inoculated into PDM and incubated overnight in the light. (**a**) Fluorescence signals of *V. dahliae* WT with RFP-labeled histones (WT/*RFP–H2B*), as well as a strain expressing *FRQ–GFP* under control of the native promoter either without (*FRQ–GFP*) or with expression of the *RFP–H2B* fusion construct (*FRQ–GFP*/*RFP–H2B*), were compared. Frq–GFP and RFP–H2b co-localized in the nuclei. Scale bar: 10 µm. (**b**,**c**) The GFP signal localization of strains producing (**b**) Frq–GFP with wild-type *FRH* (*FRQ–GFP*) or point-mutated *FRH* (*FRQ–GFP*/*FRH^R806H^*) or (**c**) Frh–GFP *(FRH–GFP*) or Frh^R806H^–GFP fusion proteins (*FRH^R806H^–GFP*) was analyzed. *V. dahliae* wild-type ectopically overexpressing *GFP* (WT/*GFP* OE) served as control. Localization of Frq–GFP and Frh–GFP was unaffected by Frh^R806H^ amino acid substitution. Scale bar: 10 µm.

As neither the endogenous *FRQ* transcript nor Frq protein levels were affected in the *FRH^R806H^* strain, another possible explanation for the deletion-like phenotype would be an altered localization of Frq due to the *FRH* point mutation. *V. dahliae* Frh was predicted to localize in nuclei, whereas Frq was predicted to be localized both nuclear and cytoplasmic. Nuclear localization of *N. crassa* Frq is required for its clock-related functions, but still most Frq protein was detected to be cytoplasmic [15,17,104]. The C-terminal part of *N. crassa* Frq, including the Frq–Frh interaction domain, is required for the regulation of Frq distribution between the nucleus and cytoplasm [17]. A portion of the mainly nuclear localized Frh was also detected in cytoplasmic extracts, and reduction of Frh amounts led to increased nuclear localization of Frq [15,17]. Therefore, localizations of the *V. dahliae* GFP-fused proteins Frq–GFP and Frh–GFP were investigated through fluorescence microscopy. Strains expressing either *FRH–GFP* or the point mutated *FRH^R806H^–GFP* construct were obtained (Figure S4). Both Frq–GFP and Frh–GFP were predominantly localized in nuclei. This localization was unaffected by *FRH^R806H^* point mutation under the tested condition (Figure 6). Thus, neither protein abundance nor localization seem to be affected by the point mutation. These observations support that the aa exchange in Frh hinders Frq from performing its functions.

**Figure 7:**
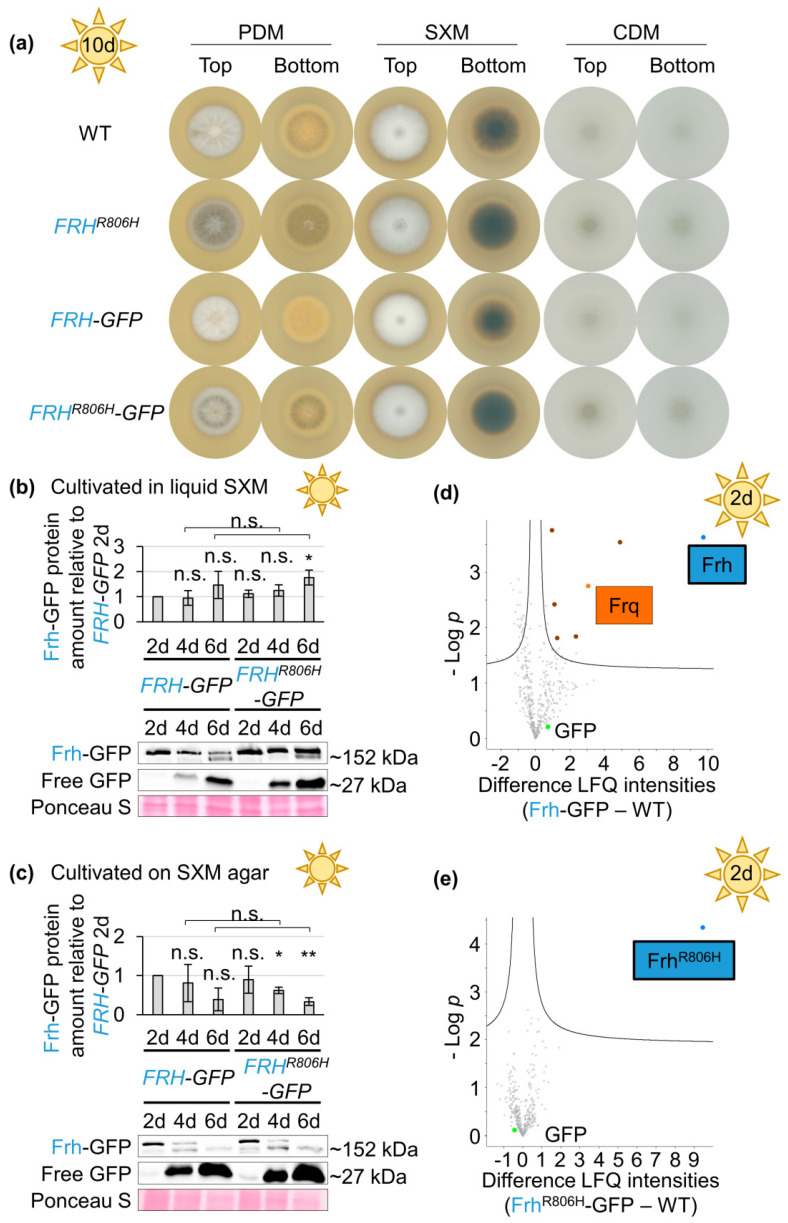
*V. dahliae* Frh arginine at position 806 is dispensable for Frh–GFP protein levels but is required for Frh-Frq interaction. (**a**) A total of 50,000 spores of *V. dahliae* wild-type (WT), an *FRH* point mutation strain (*FRH^R806H^*), and strains expressing either *FRH–GFP* or *FRH^R806H^–GFP* (*FRH–GFP*, *FRH^R806H^–GFP*) under control of the native promoter were point-inoculated onto PDM, SXM, and CDM. The phenotype was investigated after incubation at 25 °C in the light for ten days. The strain producing the Frh–GFP fusion protein grew similar to the WT. (**b**,**c**) Spores of *V. dahliae* strains expressing *FRH–GFP* and *FRH^R806H^–GFP* were inoculated (**b**) into liquid SXM (1 × 10^6^ spores) or (**c**) spread on solid SXM covered with a nylon membrane (4 × 10^6^ spores). Cultures were grown at 25 °C for two (2d), four (4d), and six days (6d). Proteins were extracted and the protein amount of Frh–GFP and Frh^R806H^–GFP (≥152 kDa) was quantified via western experiments using a GFP antibody. The pixel density of detected signals was quantified and normalized to the respective Ponceau S staining. Images of one replicate are depicted below the graphs. The fusion protein amount of the *FRH–GFP* strain after two days of cultivation was set as one. Depicted is the mean of three biological replicates with standard deviation (*N* = 3). Statistical significances were determined with *t*-tests (n.s.: not significant, *: *p* < 0.05, **: *p* < 0.01). Significances compared with Frh–GFP 2d fusion protein amount are indicated on top of the bars. Comparisons between the 4d or 6d time points are shown by connecting lines. The amino acid exchange does not significantly affect the fusion protein levels. (**d**,**e**) As many as 5 × 10^9^ spores of *V. dahliae* WT, *V. dahliae* WT with ectopically overexpressed *GFP*, *FRH–GFP*, and *FRH^R806H^–GFP* strains were inoculated into 500 mL SXM and grown at 25 °C for two days. One to three cultures were combined per sample (*N* = 1). As control, mycelium of wild-type strains with and without *GFP* overexpression were mixed approx. 1/60 (with GFP/without GFP). GFP trap pull-down was performed with total protein extracts. Peptides obtained through trypsin digestion were analyzed by LC/MS. The experiment was performed in triplicates (*N* = 3). During subsequent analysis, missing values were replaced by imputation four times. Interaction candidates found to be significant in all four imputation repetitions are colored in the volcano plots—Frh (bait): blue; Frq: orange; other significant interaction partners: brown. GFP is colored green. Proteins in the upper right part are significant interactors. (**d**) Frh interacted with six proteins, including Frq. (**e**) Frh^R806H^ did not significantly interact with any other protein.

*N. crassa* Frq and Frh form a heteromeric Frq–Frh complex [15]. Co-immunoprecipitation experiments using the protein variant with aa exchange (Frh^R806H^) in *N. crassa* suggested that the Frq–Frh complex can still be formed [29] although interaction of Frq and Frh^R806H^ seemed significantly weakened [30]. An in vitro protein pull-down was conducted to investigate whether the Frq–Frh complex is also formed in *V. dahliae* and whether complex formation is affected by the *FRH^R806H^* aa exchange. Strains expressing *FRH–GFP* or *FRH^R806H^–GFP* fusion gene constructs at the native locus were examined. Expression of the Frh–GFP fusion protein allowed for development similar to the wild-type, whereas the *FRH^R806H^–GFP*-expressing strain had the same phenotype of increased microsclerotia formation as the *FRH^R806H^* strain (Figure 7a). C-terminal GFP fusion only causes a slight reduction of fungal growth, as corresponding colonies of the *FRH–GFP* and *FRH^R806H^–GFP* strains are smaller than wild-type or *FRH^R806H^* colonies. Before the in vitro protein pull-down was conducted, production of the fusion proteins was verified via western experiments. The cultures were incubated for two, four, and six days before proteins were extracted (Figure S6). Both Frh–GFP and Frh^R806H^–GFP have a predicted molecular weight of ~152 kDa. The largest detected molecular weight of the fusion proteins in western experiments, however, was slightly above 180 kDa, which indicates additional potential post-translational modifications. Signals ≥ 152 kDa were used for quantification of Frh–GFP and Frh^R806H^–GFP protein amounts. Levels of both fusion proteins did not significantly differ from each other when cultivated under the same conditions (Figure 7b,c).

Extracts of *FRH–GFP* and *FRH^R806H^–GFP* strains from two-day cultivation in liquid SXM were used for in vitro protein pull-downs, when both Frh–GFP and Frh^R806H^–GFP fusion proteins were sufficiently and comparably detected in western experiments. *V. dahliae* wild-type protein extracts with levels of free GFP comparable to the fusion protein levels were used as control (Figure S7). Significantly enriched proteins are depicted in the top right part of the volcano plots (Figure 7d,e).

Frh–GFP interacted with Frq and five other proteins (Table S5). Predicted domains or protein family types of the identified interacting proteins are listed in Table S6. With Frh^R806H^–GFP as bait, no other proteins were significantly enriched (Table S7). This suggests that Frq–Frh interaction depends on the arginine at position 806 in the Frh protein and is abolished or at least significantly weakened by the aa exchange. These results indicate that the function of Frq in controlling *V. dahliae* development depends on Frq–Frh complex formation.

### 3.5. Sfl1 Positively Affects Microsclerotia Formation Prior to Frq and Contributes to V. dahliae Spore Formation

The role of Frq and the importance of the Frq–Frh complex in the development of *V. dahliae* have to be connected to corresponding pathways. The Som1- and Vta3-regulatory networks are involved in initial plant infection and conidia formation, as well as microsclerotia formation of *V. dahliae* [61]. The transcription factor Vta3 controls the expression of other transcription factors involved in the regulation of microsclerotia formation and conidiation. Expression of the Vta3-encoding gene is, in turn, induced by Som1, which also stimulates microsclerotia formation and conidiation by a Vta3-independent pathway [61]. We investigated if point mutation of *FRH* or deletion of *WC1* or *FRQ* affects the transcript levels of transcription factors that are part of these regulatory networks. Neither *SOM1* nor *VTA3* transcript levels did in any case significantly differ from wild-type control (Figure S15). Under conidiation-favoring conditions, transcript levels of *ABA1* were not affected by *FRQ* deletion or *FRH* point mutation. The transcript levels of *VTA2* did significantly differ from wild-type levels in the *FRH^R806H^* mutant strain after two days of incubation (liquid SXM, ~15% increased). *FRH* mutation, however, resulted in reduced conidiation (Figure 3), and Vta2 is a positive regulator of this developmental process [67]. Moreover, *VTA2* levels were reduced in the *FRQ–GFP* strain after cultivation in liquid SXM for six days (~46% reduced). Vta2 is also involved in repression of microsclerotia formation, but its transcript levels did not significantly change upon *WC1* deletion during growth on SXM agar (Figure S15). In contrast, deletion of *WC1* resulted in significantly elevated transcript levels of *CMR1* (~250% increased) and *VTA1* (~150% increased) after two days of cultivation under microsclerotia-favoring conditions (on SXM agar, Figure S15). Both are transcription factors that positively affect melanization in *V. dahliae* [68,71]. Thus, the increased demand of melanization upon enhanced microsclerotia formation in the *WC1* deletion strain seems to be mediated by elevated transcript levels of *VTA1* and *CMR1*. This supports that Wc1 and presumably also the Frq–Frh complex are involved in reducing *VTA1*- and *CMR1*-mediated melanization. The question of by which factor or factors the light-dependent microsclerotia repression is regulated remains elusive.

Sfl1 is another Vta3-regulated transcription factor that positively affects microsclerotia formation and is a potential target of Frq–Frh-mediated light-dependent microsclerotia repression [61]. The reduction of *SFL1* transcript levels by Frq could be a possible explanation for Frq’s negative effect on microsclerotia development. Quantification of *SFL1* transcript levels in the *WC1* deletion strain, however, indicated no significant influence of Wc1 and therefore presumably no influence of Frq on *SFL1* transcript levels (Figure S15). As previously described (Figure 1), deletion of *FRQ* leads to increased melanization in the light, which is due to enhanced microsclerotia formation. In contrast, deletion of *SFL1* resulted in reduced microsclerotia production in the light [61]. Sfl1 might be required for the onset of microsclerotia formation. A strain that produces a GFP–Sfl1 fusion protein was constructed to further investigate the role of Sfl1 in microsclerotia formation (Figure S5). GFP–Sfl1 was present at the initial phase of microsclerotia development, whereas the protein amounts decreased after microsclerotia were formed (Figure S16). Thus, both the inducing Sfl1 and the reducing Frq protein seem to be required early in the control of microsclerotia development.

**Figure 8:**
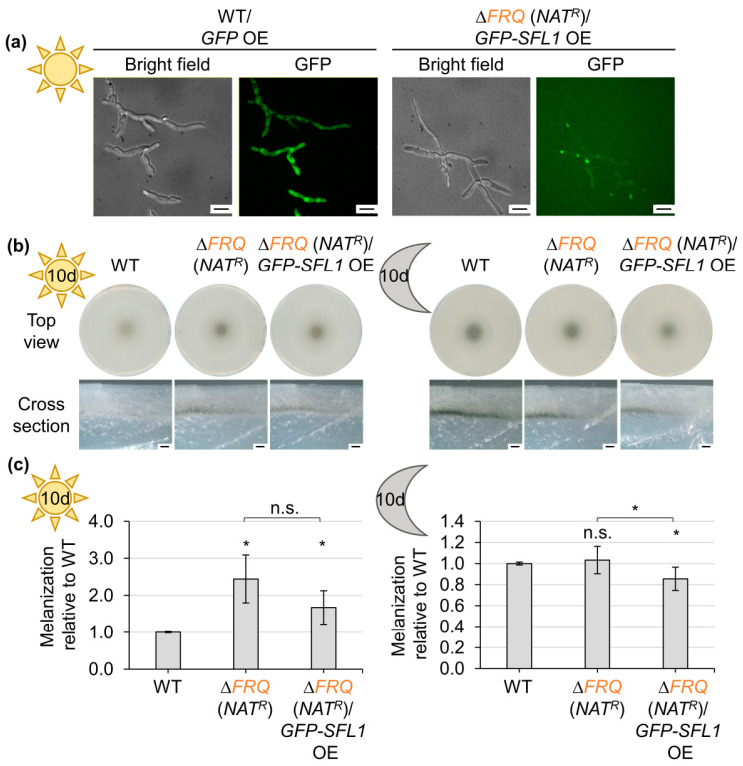
High abundance of the Suppressor of flocculation 1 (Sfl1)–GFP fusion protein in the *FRQ* deletion mutant does not lead to enhanced microsclerotia formation. GFP–Sfl1 protein localization and microsclerotia production of a *FRQ* deletion strain ectopically overexpressing *GFP–SFL1* (∆*FRQ* (*NAT^R^*)/*GFP–SFL1* OE) were analyzed. The *FRQ* deletion strain with nourseothricin resistance cassette (∆*FRQ* (*NAT^R^*)) was used as background strain. (**a**) Spores were inoculated into PDM and grown at 25 °C overnight. Subcellular localization of GFP–Sfl1 was analyzed by fluorescence microscopy. Wild-type ectopically overexpressing *GFP* (WT/*GFP* OE) served as control. GFP–Sfl1 is predominantly localized in nuclei. Scale bars = 10 µm. (**b**,**c**) A total of 50,000 spores of the *V. dahliae* wild-type (WT) and ∆*FRQ* (*NAT^R^*), as well as ∆*FRQ* (*NAT^R^*)*/GFP–SFL1* OE strains, were point-inoculated onto minimal medium (CDM). Plates were incubated at 25 °C in the light (left) or in the dark (right) for ten days. (**b**) Colonies and cross sections of the colony center are depicted. Scale bar = 500 µm. (**c**) Melanization of the colonies was quantified. The experiment was performed three times with one to two independent transformants or cultures as biological replicates (*N* = 4–6). Depicted is the mean of biological replicates with standard deviation. WT melanization was set as one. Significant differences were calculated with *t*-tests (n.s.: not significant, *: *p* < 0.05). Significant differences compared with WT are depicted above the bars. Comparisons between other strains are indicated by connecting lines. Light-incubated cultures of ∆*FRQ* (*NAT^R^*) and ∆*FRQ* (*NAT^R^*)*/GFP–SFL1* OE strains were significantly more melanized than the WT. When grown in the dark, *FRQ* deletion did not affect colony melanization, but *GFP–SFL1* overexpression led to significantly reduced colony melanization compared with WT.

Based on the opposing roles of Frq and Sfl1 in the development of microsclerotia, we tested the effect of a strong and constitutive expression of Sfl1 on the phenotype of a *FRQ* deletion strain. A *GFP–SFL1* overexpression construct was ectopically integrated into a *FRQ* deletion as well as a *SFL1* deletion strain (Figure S5). Strains that harbored the *GFP–SFL1* construct at the endogenous locus grew similar to wild-type, whereas melanization of strains with the ectopically integrated *GFP–SFL1* overexpression construct was slightly reduced in comparison with wild-type (Figure S17). The presence of the fusion protein was confirmed through fluorescence microscopy, in which the protein localized in the nuclei. Increased production of the GFP–Sfl1 fusion protein in the strain harboring the overexpression construct was verified through comparison with fusion protein amounts resulting from endogenously expressed *GFP–SFL1* in a western experiment (Figure S17). Introduction of the *GFP–SFL1* overexpression construct into a *FRQ* deletion strain was confirmed through fluorescence microscopy (Figure 8a) and western experiments (Figure S17). Deletion of *FRQ* resulted in increased melanization in the light, and ectopic reintroduction of the gene allowed for growth similar to wild-type (Figure S18). Ectopic overexpression of *GFP–SFL1* in the *FRQ* deletion strain background also led to increased colony melanization compared with the wild-type (~67% increased) during cultivation in the light (Figure 8b,c). The melanization of the *FRQ* deletion strain was similar to wild-type when cultured in the dark, whereas melanization of the *FRQ* deletion strain containing the *GFP–SFL1* overexpression construct was significantly reduced compared with wild-type as well as the *FRQ* deletion strain (~14–17% reduced; Figure 8b,c).

**Figure 9:**
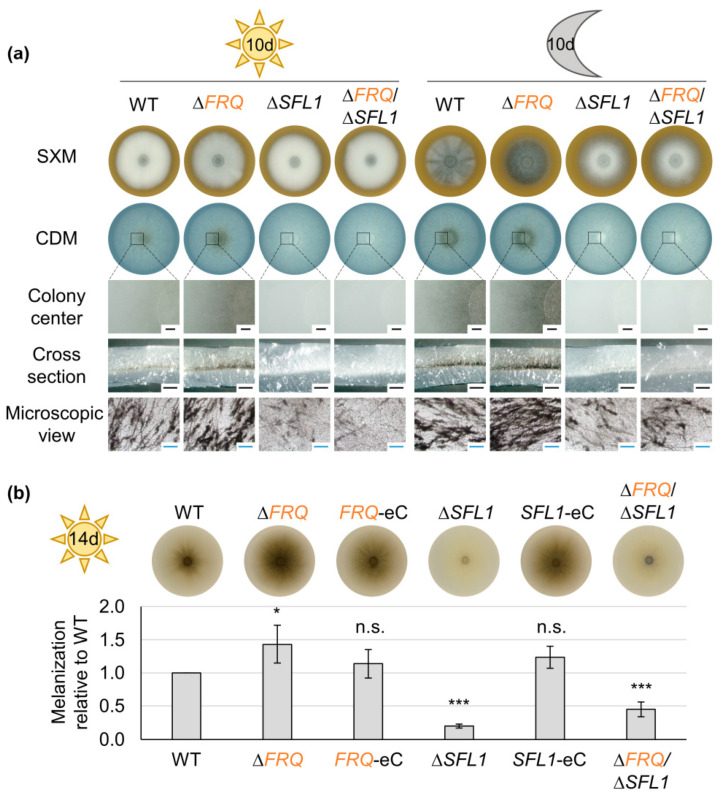
*SFL1* acts epistatically on *FRQ* in the regulatory pathway of microsclerotia formation. Microsclerotia production of *V. dahliae* wild-type (WT), *FRQ* and *SFL1* single-deletion strains (∆*FRQ,* ∆*SFL1*), ectopic complementation strains (*FRQ*-eC, *SFL1*-eC), and double-deletion strain (∆*FRQ*/∆*SFL1*) was analyzed. (**a**) A total of 50,000 spores of the strains were point-inoculated onto SXM or CDM and incubated at 25 °C in the light (left panel) or in the dark (right panel) for ten days. Top-view pictures of the colonies are shown. Of CDM cultures, the colony center without aerial hyphae, cross sections of colony centers, and microscopic views are depicted. The *FRQ* and *SFL1* double-deletion strain grew similar to the *SFL1* single-deletion strain. Black scale bars = 1 mm, blue scale bars = 100 µm. (**b**) A total of 50,000 spores were point-inoculated onto CDM and incubated at 25 °C for 14 days. Melanization of the colonies was quantified and WT was set to one. Two independent transformants (*N* = 2) of the deletion strains were tested, and one transformant (*N* = 1) was used for each of the complementation strains and wild-type. Mean values with standard deviation are depicted (*N* = 3–6). Significant differences compared with WT were calculated with *t*-tests and are indicated above the bars (n.s.: not significant, *: *p* < 0.05, ***: *p* < 0.001). The *SFL1* single-deletion and *FRQ/SFL1* double-deletion strains were significantly less melanized compared with wild-type.

The role of Frq and Sfl1 in the control of microsclerotia formation was further investigated through the construction (Figure S1) and analysis of double mutants. The *FRQ* and *SFL1* double-deletion strain grew like the *SFL1* single-deletion strain, with reduced microsclerotia formation independent of light-conditions and increased aerial hyphae formation in the dark after ten days (Figure 9a). The melanization of 14-day-old light-incubated CDM cultures was quantified (Figure 9b), and it was confirmed that the *FRQ*/*SFL1* double-deletion strain colonies were significantly less melanized compared with wild-type (~55% reduced) and the *FRQ* deletion strain (~69% reduced). The double-deletion strain was still significantly more melanized than the *SFL1* single-deletion strain (~80% reduced compared with wild-type). The respective ectopic complementation strains melanized similar to wild-type. The fact that the *FRQ*/*SFL1* double-deletion growth phenotype was more similar to the *SFL1* single-deletion strain than to the *FRQ* single-deletion strain indicates that Sfl1 is required earlier in the induction of microsclerotia formation than the repressive activity of Frq.

**Figure 10:**
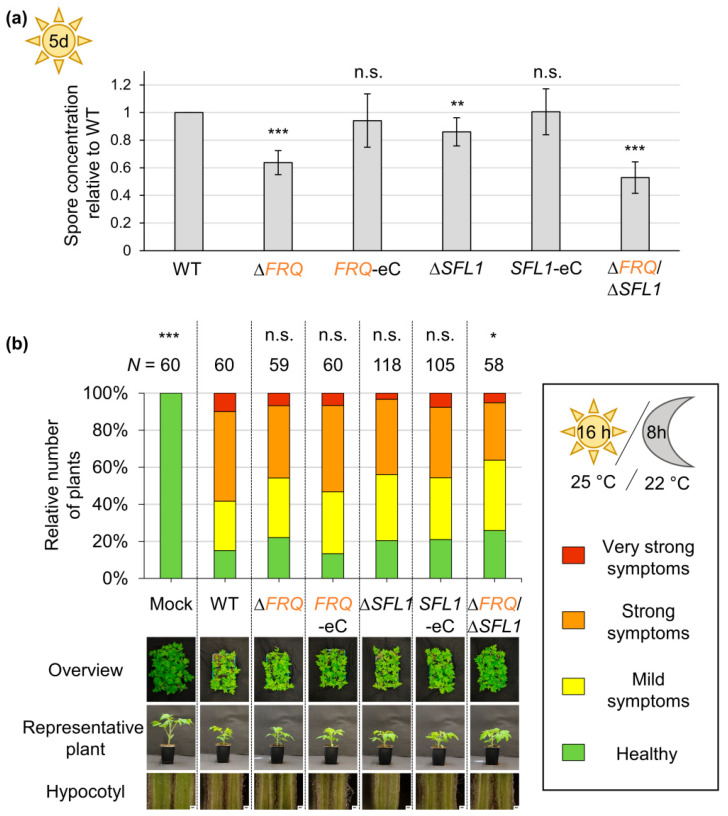
Sfl1 is, to a lesser extent than Frq, involved in induction of *V. dahliae* conidiospore production, and the combined actions of Frq and Sfl1 slightly enhance disease development. The spore production and disease induction of *V. dahliae* wild-type (WT), *FRQ* and *SFL1* single-deletion strains (∆*FRQ*, ∆*SFL1*), ectopic complementation strains (*FRQ*-eC, *SFL1*-eC), and the *FRQ* and *SFL1* double-deletion strain (∆*FRQ*/∆*SFL1*) were analyzed. (**a**) A total of 200,000 spores of the respective strains were inoculated into SXM and incubated at 25 °C for five days under agitation. Conidiospore production was quantified. One WT culture, one transformant of each complementation strain, and two independent transformants of the single- and double-deletion strains were tested. WT conidia concentration was set to one. The experiment was performed three times with all strains (complementation strains: *N* = 3) and a fourth time without the complementation strains (WT: *N* = 4, deletion strains: *N* = 8). Bars represent the mean relative concentration with standard deviation. Significant differences compared with WT were calculated with *t*-tests and are indicated above the bars (n.s.: not significant, **: *p* < 0.01, ***: *p* < 0.001). Conidiation was significantly reduced in the *SFL1* deletion strain and deletion of *FRQ* in presence or absence of *SFL1* resulted in a greater decrease in conidiation compared with WT. (**b**) Tomato plants were treated with spores of indicated strains and grown for 21 days under long-day-conditions. Two independent deletion or complementation transformants served as biological replicates. Water-inoculated plants (mock) served as control. Plant height, length of the longest leaf, and fresh weight were measured and compared with respective mock plant values to calculate the mean disease level of each plant. Thereby, plants were classified as being healthy (mean disease level = 1–1.99, green), or showing mild (mean disease level = 2–2.99, yellow), strong (mean disease level = 3–3.99, orange), or very strong (mean disease level = 4, red) symptoms. Depicted are the results of two (∆*FRQ, FRQ*-eC, ∆*FRQ*/∆*SFL1*) to four independent experiments (mock, WT, ∆*SFL1*, *SFL1*-eC) with 13 to 15 plants per biological replicate. The total number of plants (*N*) is indicated above the bars. Statistical significances compared with WT infection were calculated with Mann–Whitney *U* tests (n.s: not significant, *: *p* < 0.05). Representative pictures of plant trays, individual plants, and hypocotyl cross sections are depicted below the diagram. Only plants treated with the *FRQ*/*SFL1* double-deletion strains were slightly, but significantly less diseased.

The spore production of the *SFL1* single and *FRQ*/*SFL1* double-deletion strains was quantified in comparison with wild-type and the *FRQ* deletion strain to investigate if *SFL1* also affects conidiation (Figure 10a). Deletion of *FRQ* results in reduced conidia production compared with wild-type (~36% reduced). *SFL1* deletion strains also produced significantly less conidia (~14% reduced). This reduction, however, was significantly smaller than the *FRQ*-dependent effect. The mean conidia production of the *FRQ*/*SFL1* double-deletion strain was slightly lower than in the *FRQ* single-deletion strain (47% reduced compared with wild-type). The reduction in the *FRQ* single- and *FRQ*/*SFL1* double-deletion strains, however, did not significantly differ from each other. Altogether, *SFL1* positively affects *V. dahliae* conidiation to a lesser extent than Frq. *SFL1* is dispensable for initial colonization of *A. thaliana* roots [61]; therefore, a potential role for *SFL1* in the later stages of plant colonization was analyzed (Figure 10b). Tomato plants were inoculated with water (mock), spores of the *V. dahliae* wild-type, the *SFL1* single-, or the *FRQ*/*SFL1* double-deletion strains. The treatments with the *FRQ* single-deletion strain and respective complementation strains served as controls. Symptom severity was determined after 21 days [60,68]. Plants that were challenged with fungal spores had brownish discolored hypocotyls and were stunted compared with mock-treated plants. Deletion of *SFL1* alone, like the *FRQ* single-deletion, did not significantly affect the symptom induction. Only the plants treated with spores of the *FRQ*/*SFL1* double-deletion strain were slightly, but significantly less diseased than wild-type infected plants.

Taken together, Sfl1 plays a major role in microsclerotia formation and an additional minor role in conidiation. Through combined actions with Frq, it also contributes to disease development in tomato plants. Our data further suggest that Sfl1 is only required for the onset of microsclerotia formation but is dispensable thereafter, and that the Sfl1-induced microsclerotia formation can be light-dependently delayed by the heteromeric Frq–Frh complex.

## 4. Discussion

**Figure 11:**
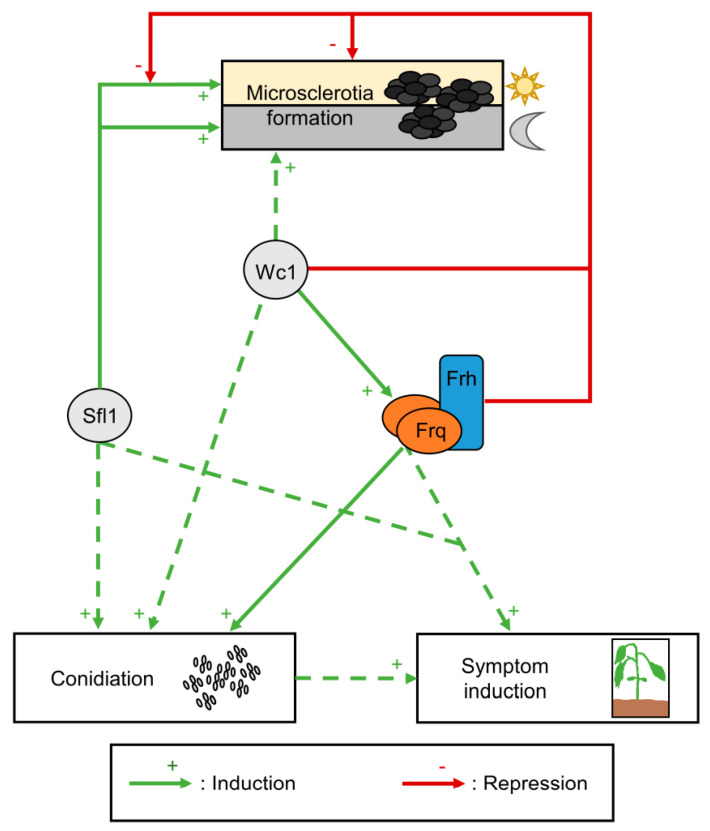
Schematic representation of Frq-, Frh-, Sfl1-, and Wc1-mediated control of *V. dahliae* development. The scheme summarizes the interplay of the investigated proteins and their functions in controlling the development of *V. dahliae*. Inducing functions are indicated by green arrows and a plus (+), whereas repressive functions are indicated by red arrows and a minus (−). Smaller effects are represented by dashed lines. The Frq–Frh complex is involved in enhanced conidiation. This developmental process is, to a lesser extent, also enhanced by Sfl1 and Wc1. Frq, Frh, Wc1, or Sfl1, individually, did not affect the symptom development in tomato plants, but the combined effects of Sfl1 and Frq slightly enhanced symptom induction in planta. This is potentially due to Sfl1- and Frq-mediated increased conidiation and thus enhanced colonization of the host plant. The control of microsclerotia formation was more complex. Sfl1 is required for enhanced microsclerotia formation regardless of the light conditions. In the light, the Frq–Frh complex mainly represses the Sfl1-dependently induced microsclerotia formation, but also the Sfl1-independently enhanced development of microsclerotia. Wc1 enhances transcription of *FRQ* and presumably thereby also represses microsclerotia formation in the light. In darkness, however, Wc1 enhances microsclerotia formation.

The *V. dahliae* JR2 clock proteins Frq and Frh form the Frq–Frh complex, which is important for Frq function. Circadian clocks generally help organisms to adapt to rhythmic changes in the environment. The *V. dahliae* Frq–Frh heteromer enhances conidiation and, during illumination, delays the Sfl1-induced microsclerotia formation for survival in the soil. Cellular Frq amounts or localization are not affected. Figure 11 summarizes these findings for *V. dahliae* Frq and Frh and their interplay with Wc1 and Sfl1 in the fungal life, plant infection, and resting structure formation cycle.

Domains of the clock proteins are well conserved [48] and there are additional similarities between *V. dahliae* Frh and Frq and corresponding *N. crassa* counterparts. *N. crassa* and *V. dahliae* Frq are both largely intrinsically disordered proteins [13,14]. *N. crassa* Frq associates with Frh, which presumably serves as nanny protein by conferring stability and structure to Frq [13,105]. Similarly, both Frh proteins carry a predicted disordered region in their N-termini [14,30]. This disordered region seems to be present only in fungi carrying *FRQ* orthologs [14]. The interaction between Frq and Frh in *N. crassa* depends on a part of the Frh N-terminus that partially overlaps with the disordered region and on a ten-aa region in the C-terminus of Frq [13,18]. We found evidence for Frq–Frh complex formation (Figure 7d and Table S5), although only four of these ten aa are conserved in *V. dahliae* Frq [18,48]. The formation of the Frq–Frh complex was abolished or at least substantially weakened upon point mutation of *FRH*, as Frq was not detected as the interaction partner of the Frh^R806H^–GFP variant (Figure 7e and Table S7).

Both Frq and Frh were localized predominantly in *V. dahliae* JR2 nuclei of hyphal cells under the tested condition (Figure 6). This is similar to *N. crassa*, *B. bassiana*, and *M. robertsii*, where Frh is also primarily a nuclear protein, except a *N. crassa* Frh subpopulation that is localized in cytoplasm [15,33,37]. Cytoplasmic Frh is presumably in complex with Frq homodimers in *N. crassa* [15]. In this fungus, Frq is localized in nuclei as well as the cytoplasm but is mainly found in the latter [15,17]. However, the Frq regulatory function requires nuclear localization [104]. The two Frq proteins of *B. bassiana* exhibit opposing rhythms of nuclear localization, which ensure approximately constant levels of nuclear Frq for promoting continuous, non-rhythmic conidiation. Incubation in light results in nuclear localization of Frq1, whereas Frq2 is localized in nuclei upon cultivation in the dark [33]. *B. bassiana* Frh mediates these localization dynamics by ensuring stable accumulation of Frq proteins. Frq–GFP fusion proteins of *M. robertsii* are mainly in nuclei, and the ratio of nuclear and cytoplasmic localization is partially mediated to a similar extent by Frh, Wc1, and Wc2 [37]. Impairment of the Frq–Frh interaction or hairpin RNA-mediated *FRH* silencing increases relative nuclear localization of *N. crassa* Frq [17]. This is different in *V. dahliae*, where nuclear localization of Frq is independent of the Frh interaction, which is impaired in a genetic background with the Frh R806H aa substitution.

All attempts to generate an *FRH* deletion strain have been unsuccessful, suggesting a potential essential function which can still be fulfilled by the Frh^R806H^ variant. Essential Frh functions have been also described for *Saccharomyces cerevisiae* Mtr4 (54.94% shared aa identity) or *N. crassa* Frh (70.05% shared aa identity) [15,106,107]. In contrast, the homologous Frh-encoding genes of ascomycete entomopathogenic fungi *B. bassiana* (72.82% shared aa identity) or *M. robertsii* (73.39% shared aa identity) are not essential [33,37].

The *FRH^R806H^* mutation had originally been described in *N. crassa*, where it disturbs Frh clock function and still allows a weakened Frq–Frh interaction [29,30]. The *N. crassa FRH* point mutation resulted in elevated Frq protein levels in combination with reduced Wc1 stability [29]. The *V. dahliae* Frh^R806H^ variant neither significantly affected Frq protein nor *FRQ* or *WC1* transcript levels compared with wild-type (Figure 5). However, *FRQ* deletion or *FRH* point mutation resulted in changes in fungal development. Conidiation was reduced and microsclerotia formation was increased in presence of light. A *WC1* deletion also resulted in increased microsclerotia formation in light but in a weaker defect in conidiation and in a reduced microsclerotia formation in the dark in comparison with the *FRQ* deletion or *FRH* codon exchange mutation (Figure 3 and Figure S12). Therefore, a functional Frq–Frh complex is required for controlling conidiation or for delaying microsclerotia formation in presence of light.

The fact that *V. dahliae* Wc1 was less important for conidiation than Frq and Frh (Figure 3) may appear unexpected, since similar phenotypes were observed regarding the repression of microsclerotia formation. A possible explanation is that Wc1 not only controls transcript levels of genes encoding proteins that promote conidiation, such as Frq, but also of those that are involved in suppression of conidiation. Likewise, the *V. dahliae* transcription factor Vta3 promotes the expression of *SFL1* and *VTA1*, which encode two transcription factors that induce microsclerotia formation and melanin production, respectively [61,68]. However, Vta3 also enhances the expression of *VTA2*, which encodes a transcription factor involved in the repression of microsclerotia formation [61,67]. This might reflect a fine-tuned regulatory process. Conidiation was also enhanced by the Frq-, Wc1-, and Wc2-encoding homologs of *B. bassiana* and *M. robertsii*, FgWc1 and FgWc2 of *F. graminearum*, as well as MoFrq, MoFwd1, and to a seemingly lesser extent, MgWc-1 of *M. oryzae* [33,35,36,37,42,45,46]. In *B. cinerea*, BcFrq1 is relevant for macroconidiation [40]. In contrast, the WCC of *B. cinerea* and ZtWco-1 of *Zymoseptoria tritici* suppress production of conidia and micropycnidiospores, respectively [41,43]. The *B. cinerea* WCC-mediated suppression of conidia formation is accompanied by enhanced aerial hyphae formation [41]. Similarly, Wc1 is presumably involved in aerial hyphae development in *V. dahliae* (Figure 2 and Figure S13) and in *F. oxysporum* [47]. In contrast to our observation that both *FRQ* deletion and *FRH* point mutation resulted in similar reductions of *V*. *dahliae* conidiation, the Frq and Frh proteins of *B. bassiana* play different roles in controlling conidiation. *B. bassiana* Frq is required and Frh is dispensable for conidiation [33]. Frq and Frh of *M. robertsii* both enhance conidiation under certain light conditions, with Frh playing a more prominent role than Frq [37]. These comparisons support a broad range and high variability in the regulatory functions for these two proteins between different fungi.

Conidiation is essential for propagation within the plant, whereas formation of microsclerotia is necessary for survival in soil and the next plant infection in the *V. dahliae* life cycle [51,54]. Wc1 is required for light-dependent repression of microsclerotia formation, presumably through transcriptional activation of *FRQ* expression and the resulting Frq–Frh complex (Figure 1, Figure 2 and Figure S11). The Frq–Frh complex is probably dispensable for the control of microsclerotia formation in the dark, when Wc1 contributes to microsclerotia formation. Similarly, *Z. tritici* ZtWco-1 positively regulates melanization and *B. cinerea* BcFrq1 represses the formation of sclerotia in a medium- and light-dependent manner [40,43]. *V. dahliae* JR2 Wc1, and presumably also the Frq–Frh complex reduce *VTA1* and *CMR1* transcript levels to suppress melanin production. In contrast, *V. dahliae* 12253 Frq does not control transcript levels of either *VTA1*, *CMR1*, or their downstream regulated target gene, *PKS1* [48,68,71]. This underlines that the regulatory functions of clock proteins vary even within different isolates of the same fungal species.

The transcription factor Sfl1 induces microsclerotia formation in *V. dahliae* [61]. However, overexpression of *GFP–SFL1* did not enhance microsclerotia formation when compared with wild-type. Elevated levels of Sfl1 might be inefficient in transcriptional activation as not enough cofactors can be recruited [108]. The Frq–Frh complex represses this Sfl1-mediated induction at early time points in light. Furthermore, the Frq–Frh complex also represses Sfl1-independently induced microsclerotia formation, as the *FRQ/SFL1* double-deletion strain melanized more than the *SFL1* single-deletion strain (Figure 9). Thus, the Frq–Frh complex might interfere with the downstream signaling of Sfl1 or its regulated targets, as well as other so far unknown inducer(s) of microsclerotia formation. Further work will be required to shed light on the exact regulatory molecular mechanism.

Only the combined actions of Frq and Sfl1 can significantly enhance symptom induction in *V. dahliae* JR2-infected tomato plants (Figure 10). The reduced conidial production of the double-deletion strain presumably results in a slowed colonization of host plants. The individual clock proteins did not significantly affect symptom induction. Similarly, individual clock proteins are not required for disease induction by other fungi. *B. bassiana* Wc1 and Wc2 are dispensable for the infection cycle and *M. robertsii* Frq does not affect fungal virulence [35,37]. Likewise, *F. oxysporum* and *F. graminearum* Wc1 proteins are dispensable for disease induction in plants [46,47]. However, clock proteins can also enhance virulence of pathogenic fungi. *M. robertsii* Wc1 and Wc2 increase virulence of the insect pathogen during cuticle infection [36]. The *B. bassiana* Frq proteins are important for full virulence, the secretion of cuticle-degrading Pr1 proteases, and the formation of blastospores, which are required for proliferation in the wax moth *G. mellonella* [34]. Deletion of *Z. tritici ZtWCO-1* delays disease progression on wheat, and *M. oryzae* MoFwd1 and MoFrq1 enhance lesion development on leaves of rice or barley [42,43]. *B. cinerea* BcWcl1 increases lesion formation in a light-dependent manner and BcFrq1 mediates the production of macroconidia required for host infection in a medium-dependent manner [40,41]. *B. cinerea* causes a more severe infection on *A. thaliana* if the first contact between fungus and plant occurs at dusk compared with dawn. This time-of-day-dependent infection severity is mediated by BcFrq1. Deletion of *BcFRQ1* results in a strong infection, independent of the time of day, whereas overexpression of *BcFRQ1* leads to reduced lesion size and spread on *A. thaliana* leaves [40]. Thus, BcFrq1 and the circadian clock regulation of *B. cinerea* reduce lesion formation at dawn. Likewise, *M. oryzae* MgWc-1 light-dependently suppresses disease [44]. This indicates a broad range of functions for clock proteins, ranging between promoting, repressing, and being dispensable for disease induction, respectively. *V. dahliae* Frq can even play different roles in the disease induction of two different strawberry-infecting strains. Frq is required for the full symptom induction of a moderately virulent strain, whereas it is dispensable for disease induction by a highly virulent isolate [48]. Thus, in some fungi, the function of an individual clock protein is already required for disease progression, whereas Frq is only important for symptom induction of *V. dahliae* JR2 in combination with Sfl1.

Altogether, we have shed light on the function of clock gene homologs in the development of *V. dahliae* JR2. The Frq–Frh complex is required for enhanced production of conidiospores and controlled formation of Sfl1-induced microsclerotia. These are both important structures in the plant-pathogenic lifestyle of *V. dahliae*. Gaining further knowledge on the regulation of these developmental programs and the role of the clock proteins therein may one day help to find new ways to combat Verticillium wilt disease.

## Data Availability

The mass spectrometry proteomics data have been deposited to the ProteomeXchange Consortium via the PRIDE partner repository with the dataset identifier PXD041716. Other data supporting reported results can be found in the Supplementary Material (Data S1 and Images S1).

## Supplementary Materials

The following supporting information can be downloaded at: https://www.mdpi.com/article/10.3390/jof9070725/s1, Methods S1 [25,26,28,60,61,67,68,69,70,71,75,76,77,79,81,97,109,110]; Figure S1: Verification of *V. dahliae FRQ* deletion, ectopic complementation, and *FRQ*/*SFL1* double-deletion strains; Figure S2: Verification of *V. dahliae FRQ* in locus complementation and *FRQ–GFP*-expressing strains; Figure S3: Verification of *WC1* deletion, complementation, and *WC1–GFP*-expressing strains; Figure S4: Verification of *FRH* point mutation, complementation, and *FRH*–*GFP*-expressing strains; Figure S5: Verification of the *V. dahliae* strains with *GFP–SFL1* in locus or ectopically overexpressed *GFP–SFL1* in either *SFL1* or *FRQ* (*NAT^R^*) deletion strains; Figure S6:* V. dahliae FRQ* and *WC1* deletion as well as *FRH* point mutation strains melanize stonger than wild-type if cultivated in liquid or on solid SXM; Figure S7: Amount of free GFP in the wild-type control is similar to Frh–GFP and Frh^R806H^–GFP protein levels; Figure S8: *V. dahliae FRQ*, *FRH*, and *WC1* genes with deduced protein structures; Figure S9: Alignments of Wc1 and Frq protein sequences from *N. crassa* and *V. dahliae* JR2; Figure S10: *V. dahliae FRQ, FRH*, and *WC1* mutant strains continuously melanize faster than wild-type; Figure S11: *FRQ–GFP* overexpression is prevented independent of Frh amino acid residue arginine 806 and the transcript level of *FRQ* depends partially, but not solely on Wc1; Figure S12: Wc1 slightly enhances microsclerotia formation in the dark and the *WC1–GFP* strain produces the Wc1–GFP fusion protein; Figure S13: Ex planta phenotypes of the *WC1/FRQ* double-deletion or *WC1*/*FRH^R806H^* double mutant strains equal the *WC1* single-deletion strain phenotype; Figure S14: The Frq–GFP protein level depends on the cultivation conditions, which favor different developmental processes; Figure S15: Wc1-, Frq-, and Frh-mediated control of *V. dahliae* development seems independent of the Som1- and Vta3-regulatory networks; Figure S16: Sfl1 is required in the initial phase of microsclerotia production; Figure S17: The GFP–Sfl1 fusion protein is present, functional, and predominantly localized in nuclei; Figure S18: Ectopic *FRQ* complementation in the ∆*FRQ* (*NAT^R^*) background allows for wild-type-like growth; Table S1: Primers used in this study; Table S2: Plasmids used in this study; Table S3: List of bacterial and fungal strains used in this work; Table S4: Oligonucleotides for qRT-PCR; Table S5: Significantly enriched proteins with LFQ intensities, MS/MS counts, sequence coverage, and unique peptides in all three replicates of Frh–GFP in comparison with the wild-type control; Table S6: Predicted protein domains or family types of proteins significantly enriched with Frh–GFP; Table S7: Significantly enriched protein with LFQ intensities, MS/MS counts, sequence coverage, and unique peptides in all three replicates of Frh^R806H^–GFP in comparison with the wild-type control; Data S1. and Images S1.
