# Supplementary material for: The Frq–Frh Complex Light-Dependently Delays Sfl1-Induced Microsclerotia Formation in Verticillium dahliae
Source: J Fungi (Basel). 2023 Jul 4;9(7):725. doi: 10.3390/jof9070725 (PMC10381341; doi:10.3390/jof9070725)
Supplement: Supplementary file 1 [file jof-09-00725-s001.zip › Images_S1.pdf]

Supplementary material

Supplementary images S1

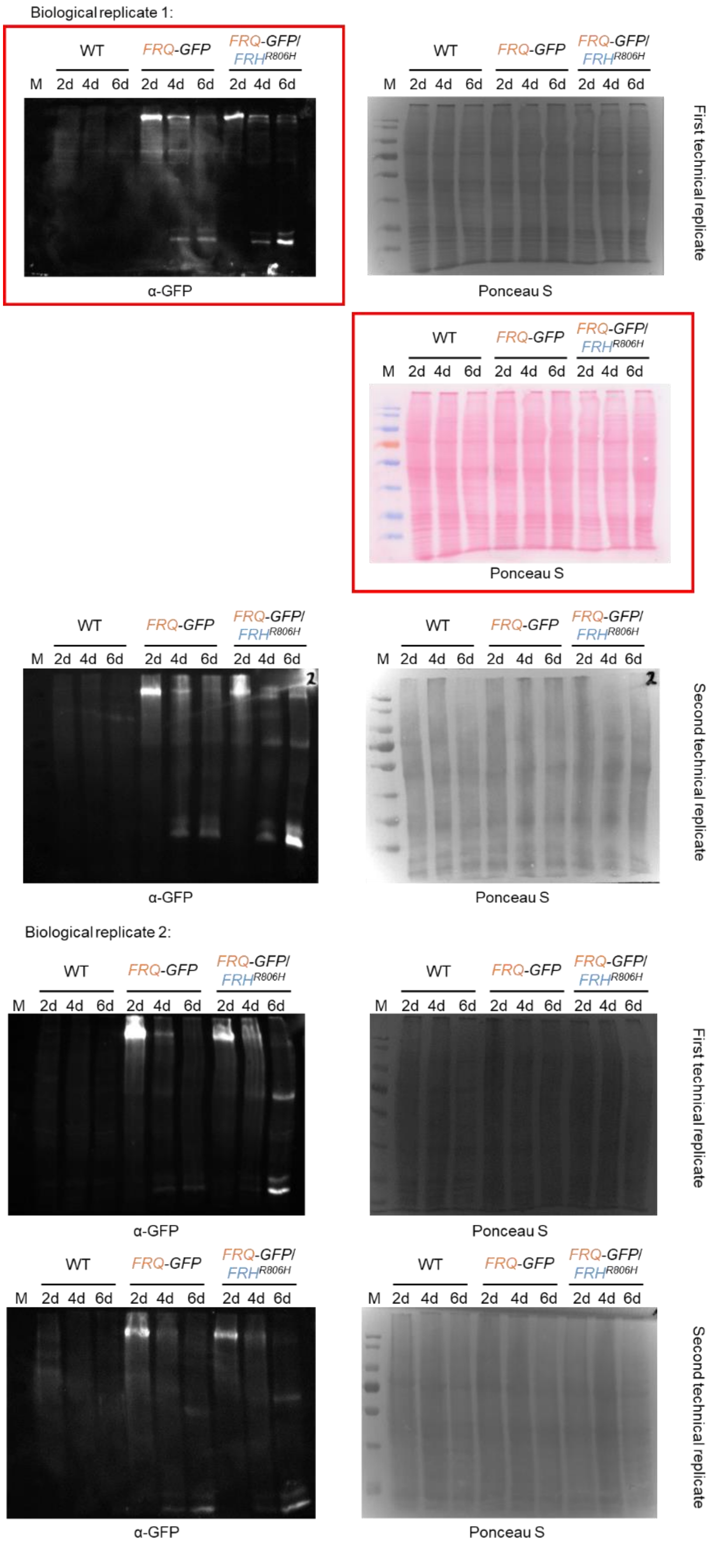

Biological replicate 3:

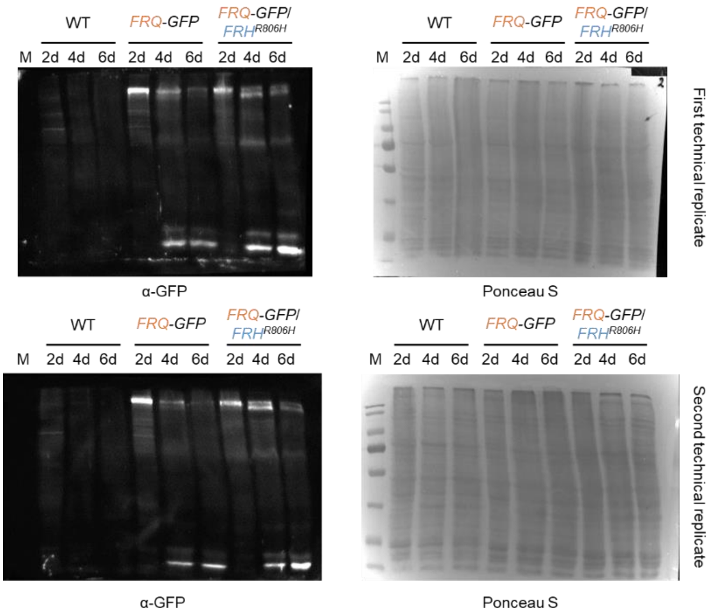

Images Figure 5c. Immunoblot of *V. dahliae* strains expressing *FRQ-GFP* at the endogenous locus in presence of wild-type *FRH* (*FRQ-GFP*) or point mutated *FRH* (*FRQ-GFP*/*FRH*^{R806H}).

V. dahliae wild-type (WT) served as negative control. Strains were cultivated in liquid SXM for two, four and six days (2d, 4d, 6d) in the light. M: PageRuler Prestained Protein Ladder 10–180 kDa (Thermo Fisher Scientific). Depicted are the three biological replicates with the respective two technical replicates used for the quantification. The grey Ponceau S images were used for normalization and the colored Ponceau S image was used for the figure. The red boxes mark the images used for the figure.

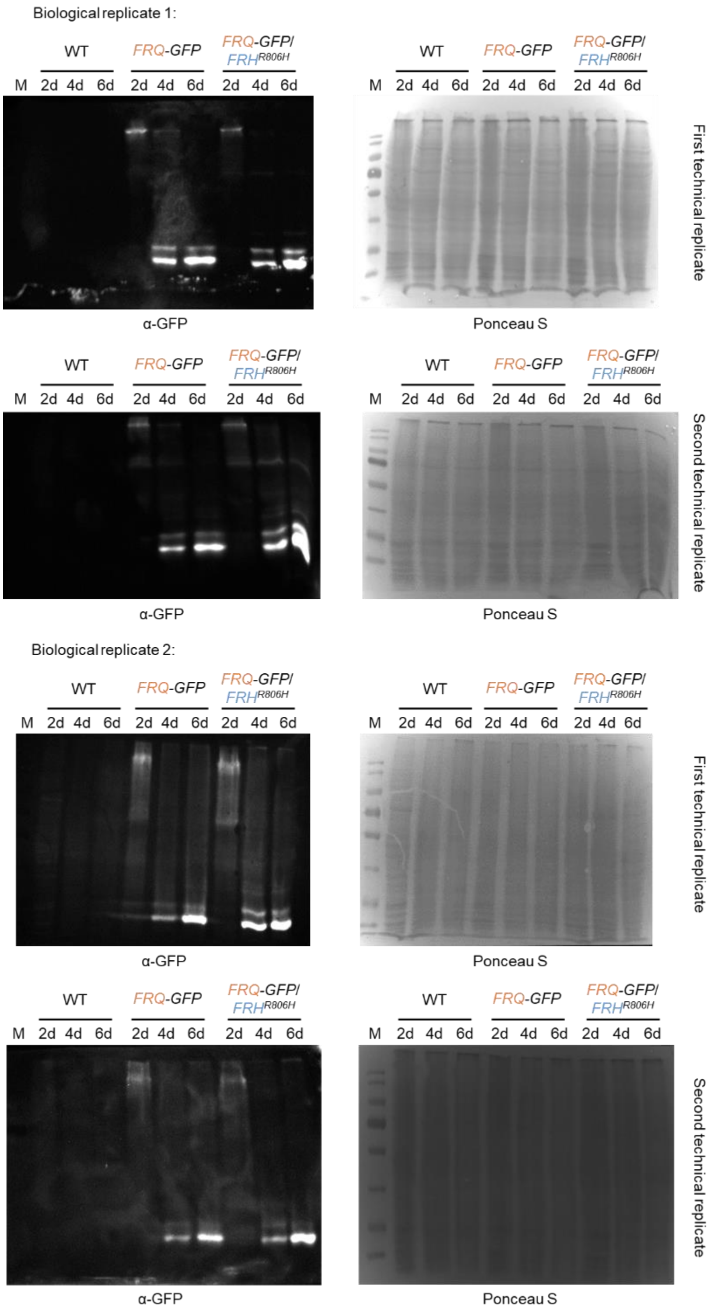

Biological replicate 1:
WT
FRQ-GFP
FRQ-GFP/ FRH^{R806H}
M 2d 4d 6d 2d 4d 6d 2d 4d 6d
α-GFP
Ponceau S
First technical replicate
Second technical replicate
Biological replicate 2:

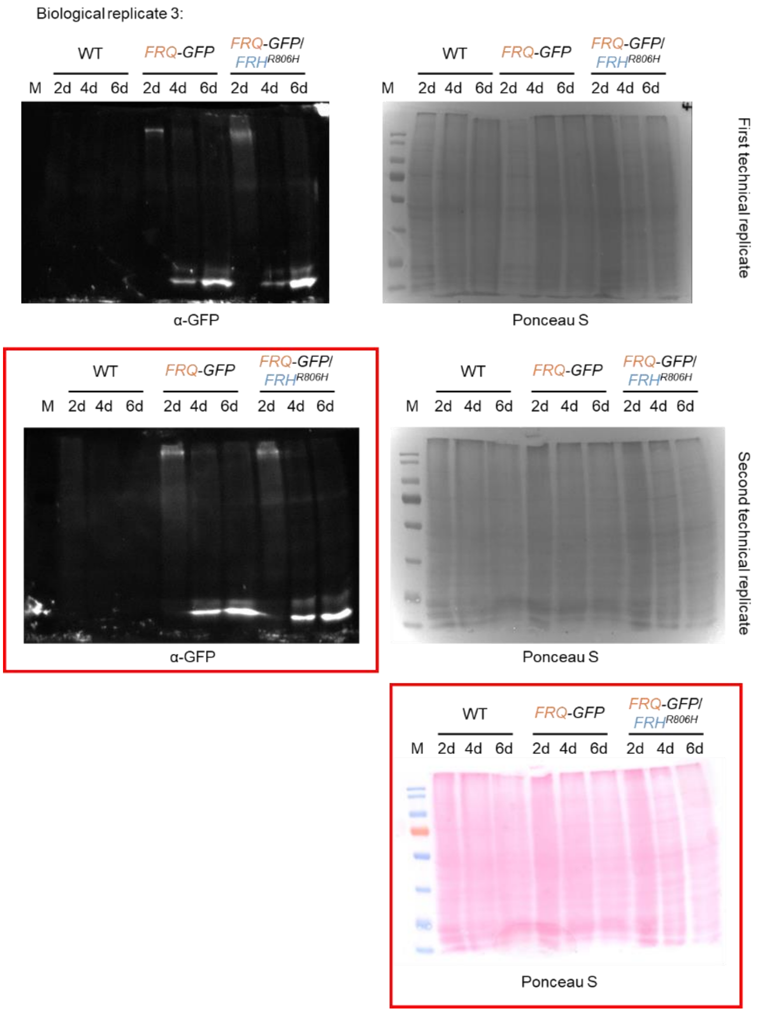

Images Figure 5d. Immunoblot of *V. dahliae* strains expressing *FRQ-GFP* at the endogenous locus in presence of wild-type *FRH* (*FRQ-GFP*) or point mutated *FRH* (*FRQ-GFP/FRH^{R806H}*).

V. dahliae wild-type (WT) served as negative control. Strains were cultivated on SXM agar covered with a nylon membrane for two, four and six days (2d, 4d, 6d) in the light. M: PageRuler Prestained Protein Ladder 10–180 kDa (Thermo Fisher Scientific). Depicted are the three biological replicates with the respective two technical replicates used for the quantification. The grey Ponceau S images were used for normalization and the colored Ponceau S image was used for the figure. The red boxes mark the images used for the figure.

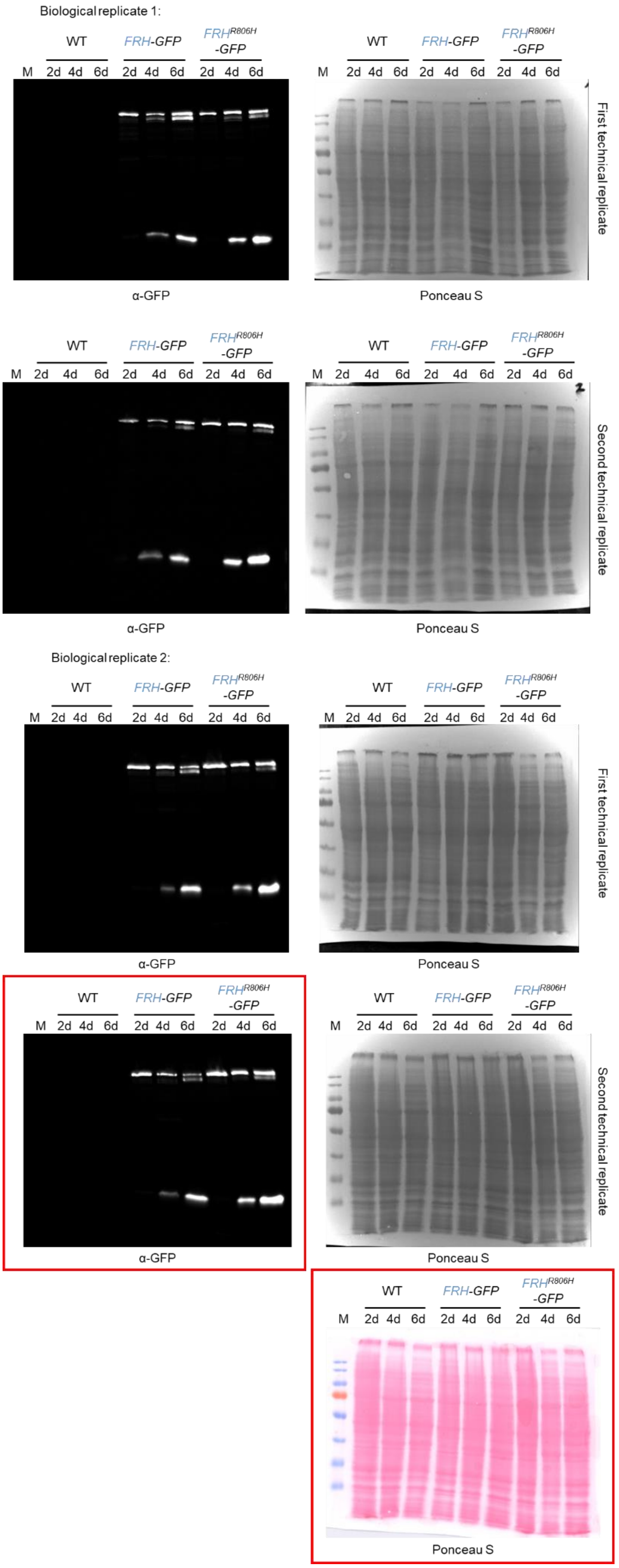

Biological replicate 1:
WT
FRH-GFP
FRH R806H -GFP
M 2d 4d 6d 2d 4d 6d 2d 4d 6d
α-GFP
Ponceau S
First technical replicate
Second technical replicate
Biological replicate 2:

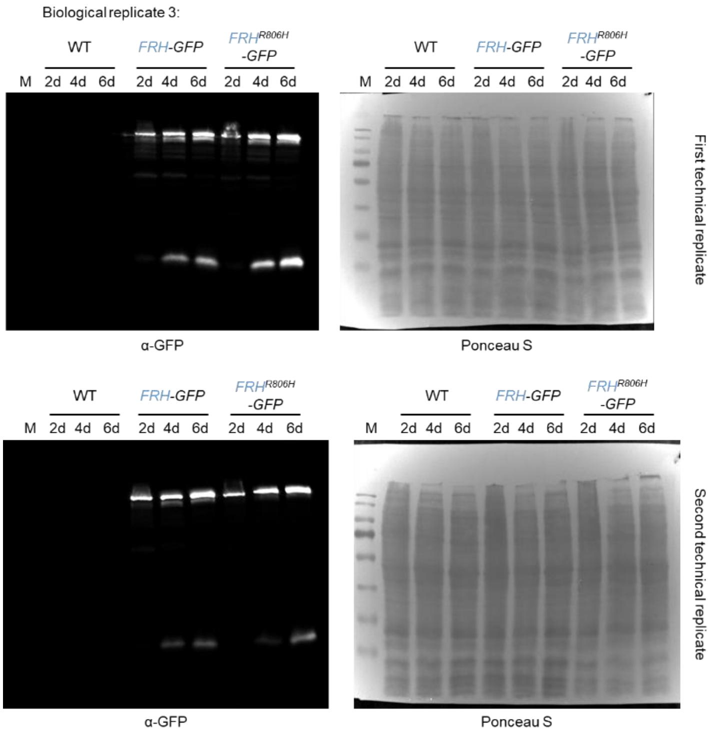

Images Figure 7b. Immunoblot of *V. dahliae* strains expressing *FRH-GFP* or point mutated *FRH*^{R806H}*-GFP* at the endogenous locus. *V. dahliae* wild-type (WT) served as negative control. Strains were cultivated in liquid SXM for two, four and six days (2d, 4d, 6d) in the light. M: PageRuler Prestained Protein Ladder 10–180 kDa (Thermo Fisher Scientific). Depicted are the three biological replicates with the respective two technical replicates used for the quantification. The grey Ponceau S images were used for normalization and the colored Ponceau S image was used for the figure. The red boxes mark the images used for the figure.

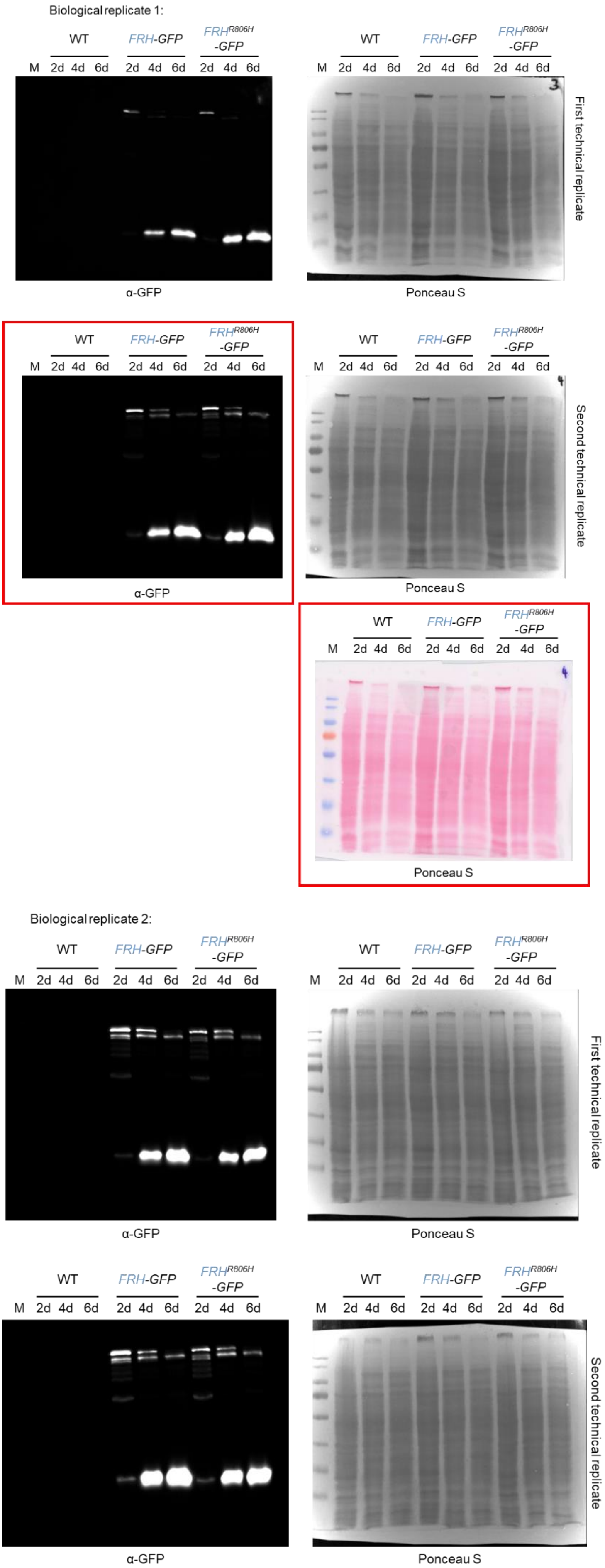

Biological replicate 1:
WT
FRH-GFP
FRH^{R806H}-GFP
M 2d 4d 6d 2d 4d 6d 2d 4d 6d
α-GFP
Ponceau S
First technical replicate
Second technical replicate
Biological replicate 2:
WT
FRH-GFP
FRH^{R806H}-GFP
M 2d 4d 6d 2d 4d 6d 2d 4d 6d
α-GFP
Ponceau S
First technical replicate
Second technical replicate

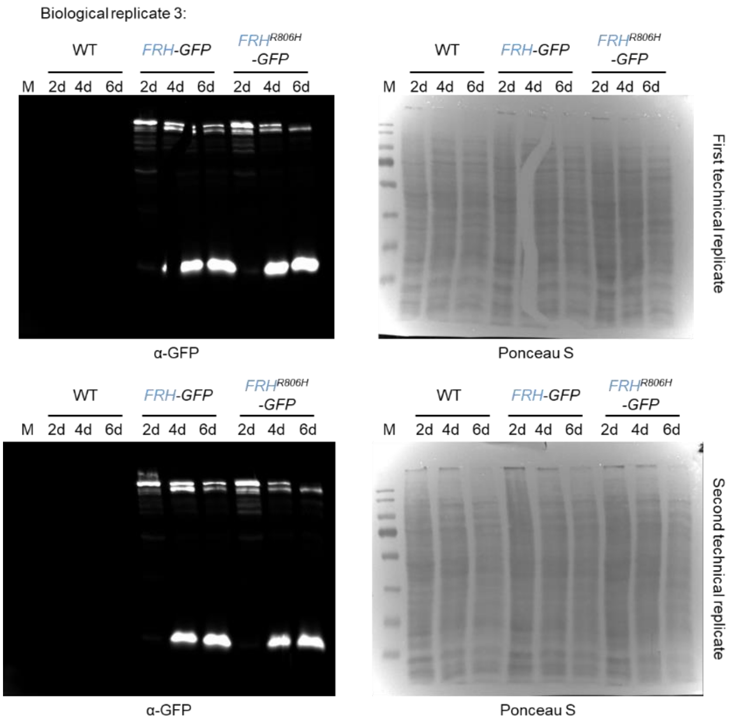

Images Figure 7c. Immunoblot of *V. dahliae* strains expressing *FRH-GFP* or point mutated *FRH*^{R806H}*-GFP* at the endogenous locus. *V. dahliae* wild-type (WT) served as negative control. Strains were cultivated on SXM agar covered with a nylon membrane for two, four and six days (2d, 4d, 6d) in the light. M: PageRuler Prestained Protein Ladder 10–180 kDa (Thermo Fisher Scientific). Depicted are the three biological replicates with the respective two technical replicates used for the quantification. The grey Ponceau S images were used for normalization and the colored Ponceau S image was used for the figure. The red boxes mark the images used for the figure.

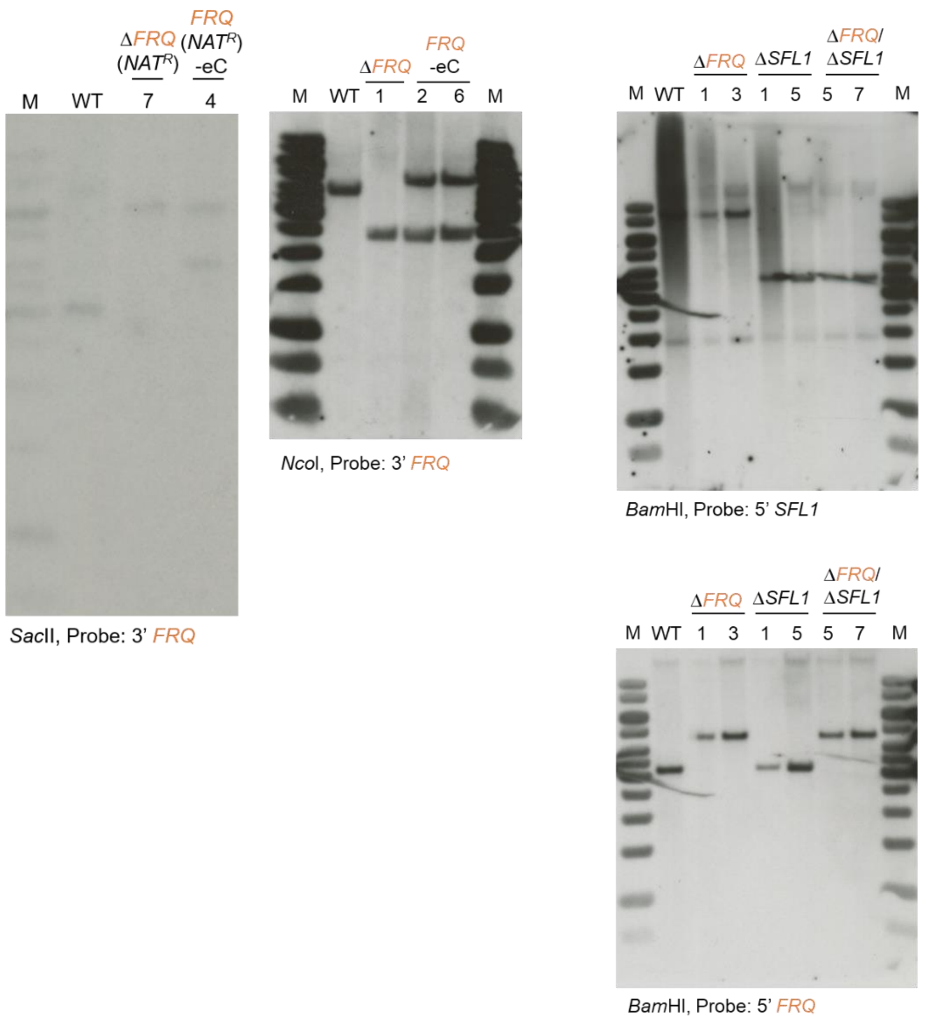

Images Figure S1d. Southern hybridizations of *V. dahliae* wild-type (WT) and *FRQ* and *SFL1* single (Δ*FRQ*, Δ*SFL1*) and double deletion strains (Δ*FRQ*/Δ*SFL1*) as well as complementation strains. M: GeneRuler 1 kb DNA Ladder (Thermo Scientific).

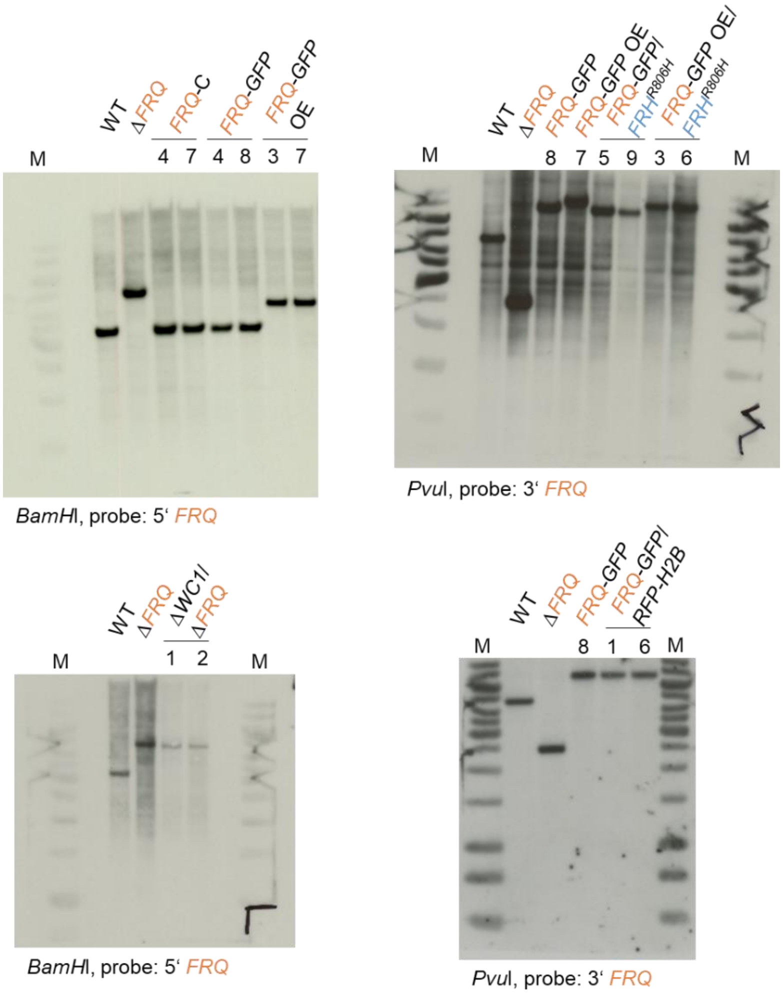

Images Figure S2b-e. Southern hybridizations of *V. dahliae* wild-type (WT) and *FRQ* mutant strains. M: GeneRuler 1 kb DNA Ladder (Thermo Scientific).

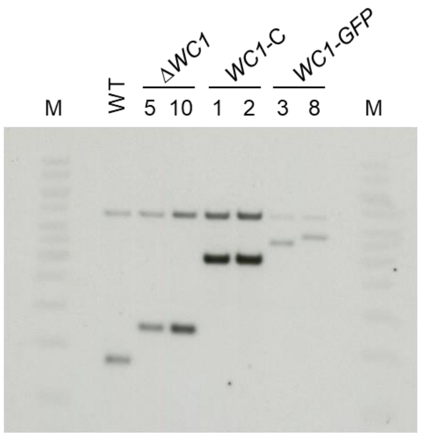

*Pst*I, probe: 3‘ *WC1*

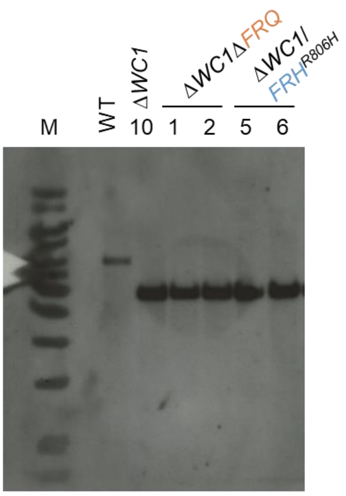

*Nco*I, probe: 3‘ *WC1*

Images Figure S3b and c. Southern hybridizations of *V. dahliae* wild-type (WT) and *WC1* mutant strains. M: GeneRuler 1 kb DNA Ladder (Thermo Scientific).

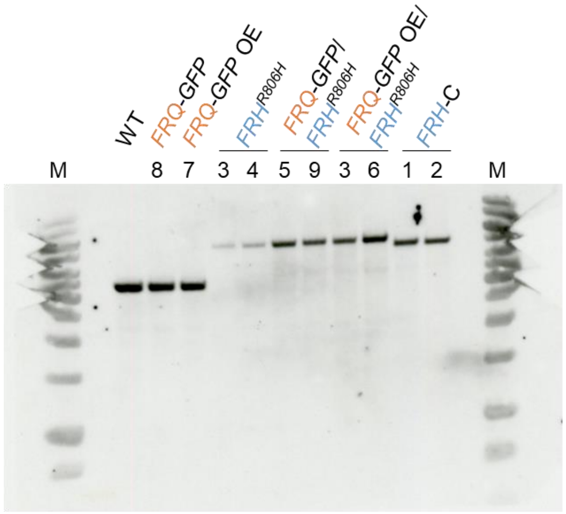

*Xho*I, probe: 3‘ *FRH*

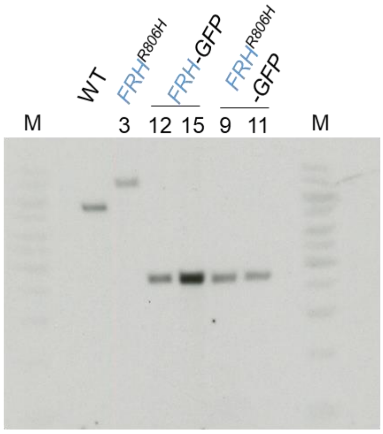

*Sma*I, probe: 3‘ *FRH*

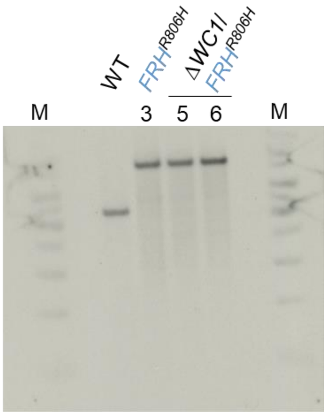

*Xho*I, probe: 3‘ *FRH*

Images Figure S4b-d. Southern hybridizations of *V. dahliae* wild-type (WT) and *FRH* mutant strains. M: GeneRuler 1 kb DNA Ladder (Thermo Scientific).

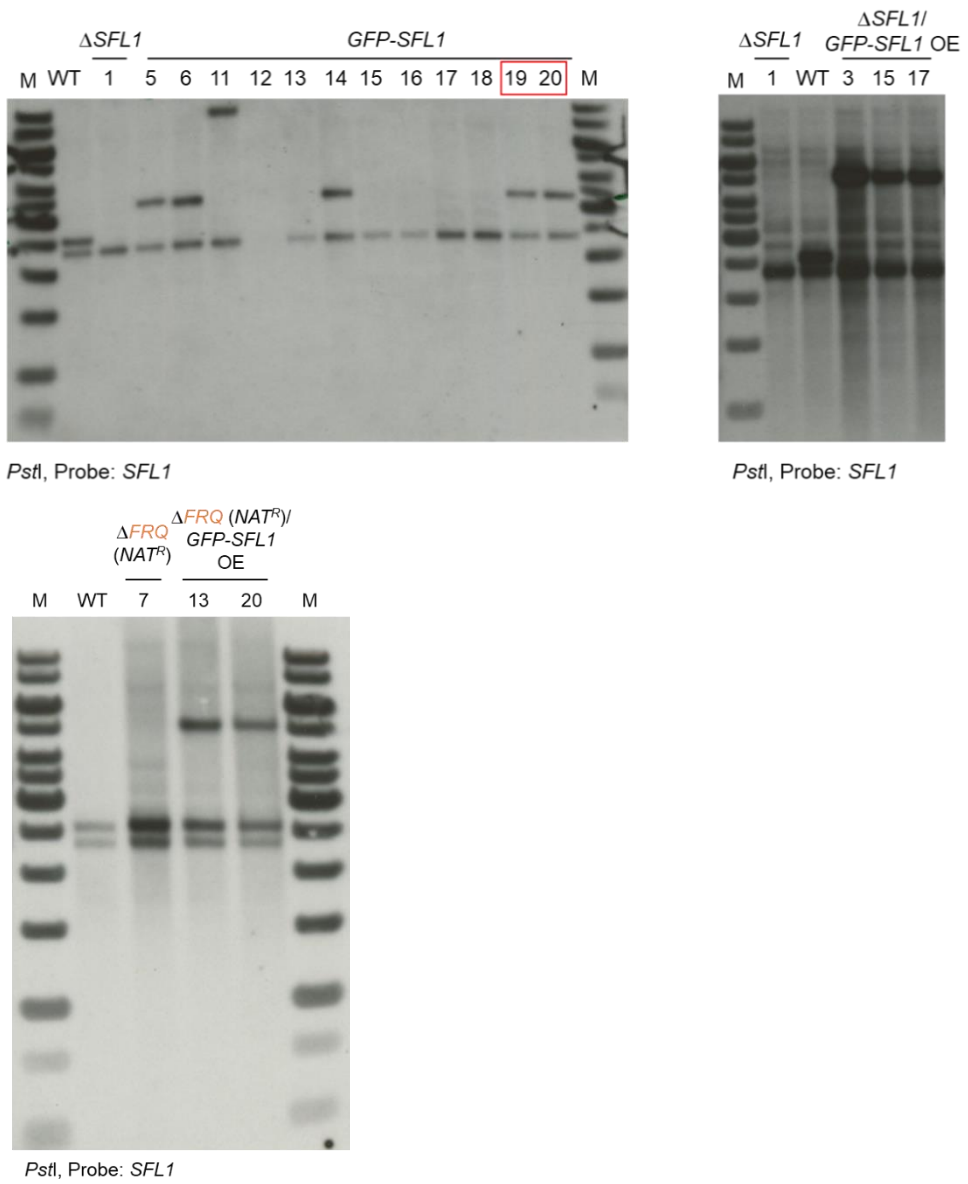

Images Figure S5b. Southern hybridizations of the *V. dahliae* wild-type (WT) and *SFL1* and *FRQ* mutant strains. Red box: verified *GFP-SFL1* transformants further worked with. M: GeneRuler 1 kb DNA Ladder (Thermo Scientific).

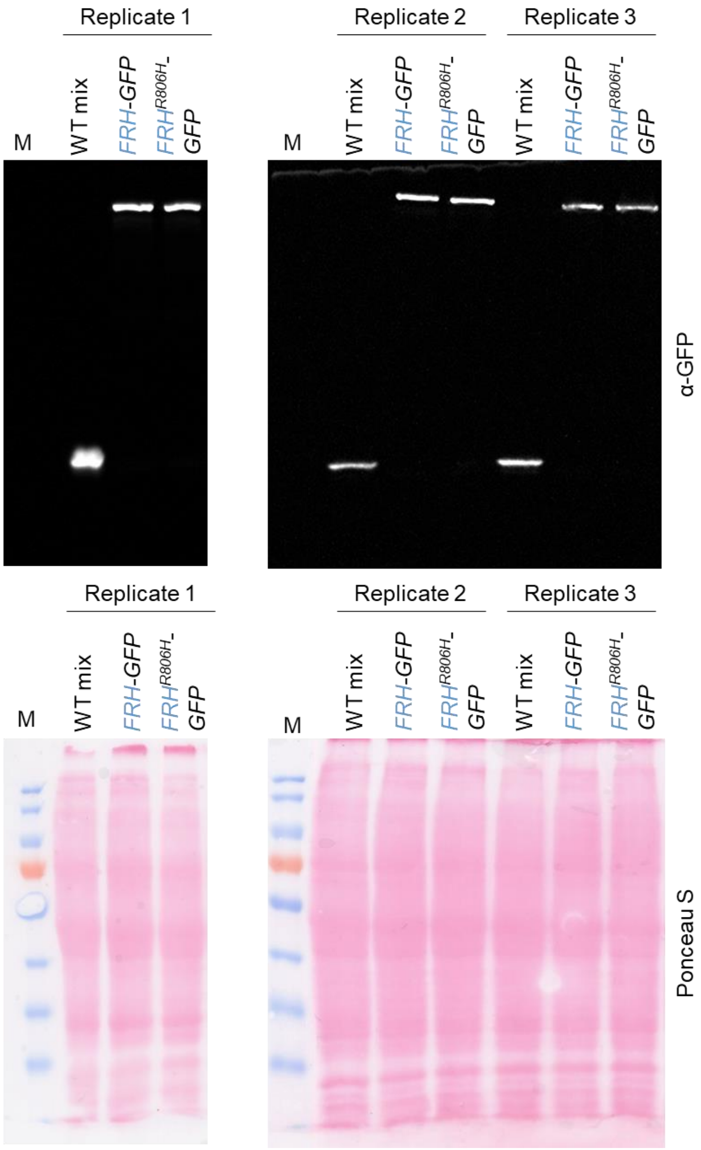

Images Figure S7. Immunoblot of *V. dahliae* wild-type (WT) mix (WT mix: 1/60 WT with free GFP, 59/60 WT) and strains expressing either *FRH-GFP* or *FRH*^{R806H}*-GFP* at the endogenous locus. Proteins were extracted after two days of cultivation in liquid SXM in the light. M: PageRuler Prestained Protein Ladder 10–180 kDa (Thermo Fisher Scientific).

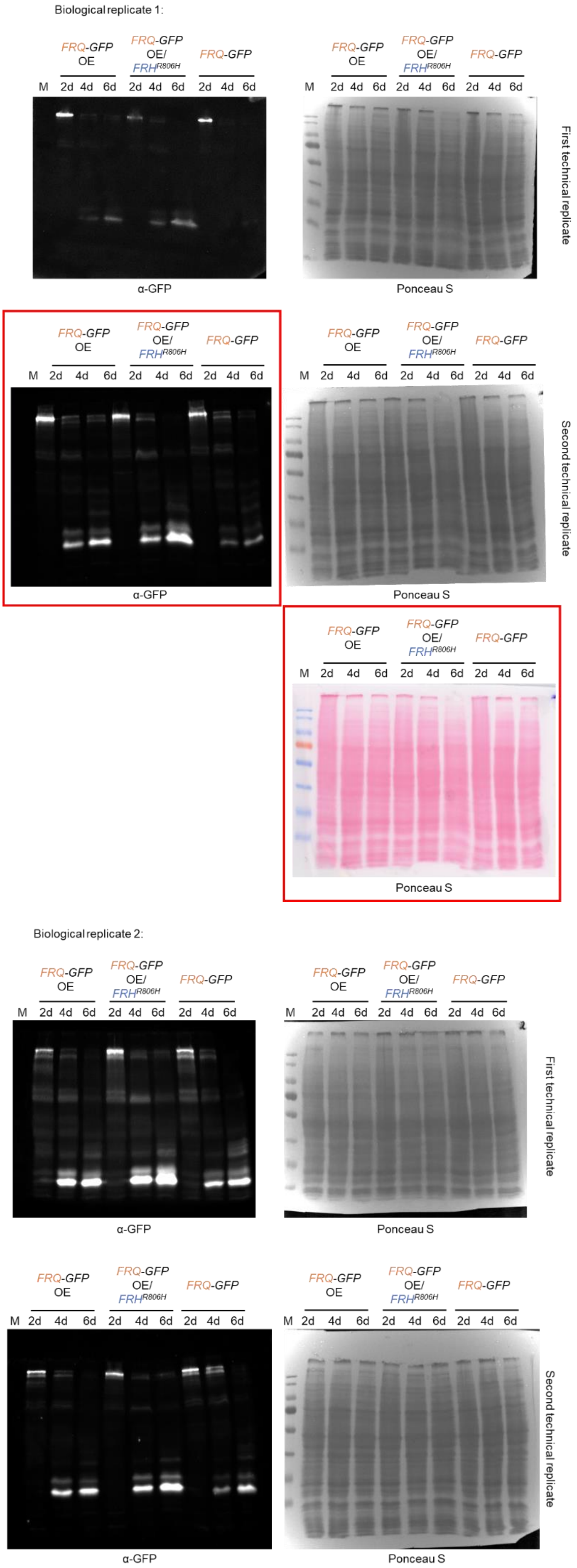

Biological replicate 1:
FRQ-GFP OE
FRQ-GFP OE/ FRH^R806H
FRQ-GFP
M 2d 4d 6d 2d 4d 6d 2d 4d 6d
α-GFP
Ponceau S
First technical replicate
Second technical replicate
Biological replicate 2:

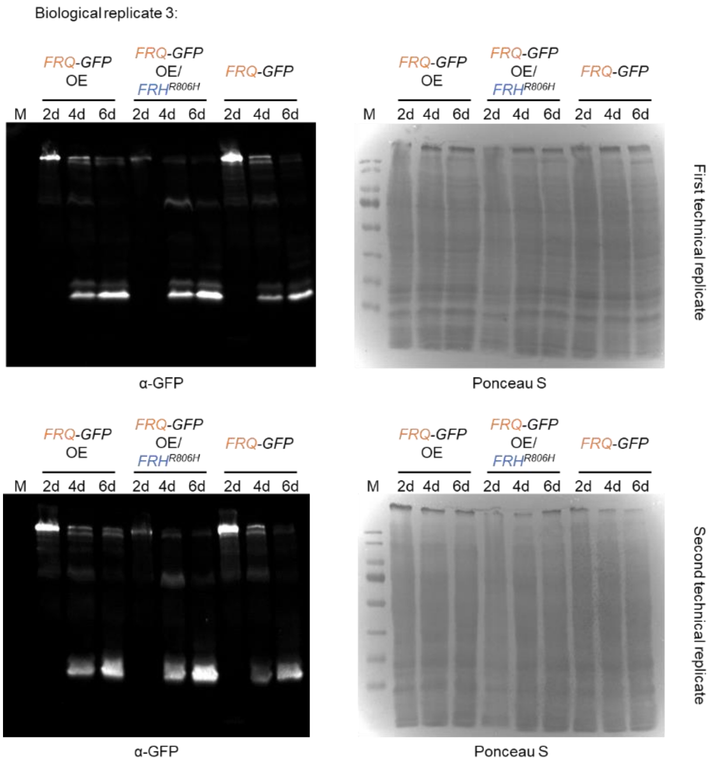

Images Figure S11c. Immunoblot of *V. dahliae* strains expressing *FRQ-GFP* at the endogenous locus under control of the native promoter (*FRQ-GFP*) or under control of the *gpdA* promoter either in presence of wild-type *FRH* (*FRQ-GFP* OE) or point mutated *FRH* (*FRQ-GFP* OE/*FRH*^{R806H}). *V. dahliae* wild-type (WT) served as negative control. Strains were cultivated in liquid SXM for two, four and six days (2d, 4d, 6d) in the light. M: PageRuler Prestained Protein Ladder 10–180 kDa (Thermo Fisher Scientific). Depicted are the three biological replicates with the respective two technical replicates used for the quantification. The grey Ponceau S images were used for normalization and the colored Ponceau S image was used for the figure. The red boxes mark the images used for the figure.

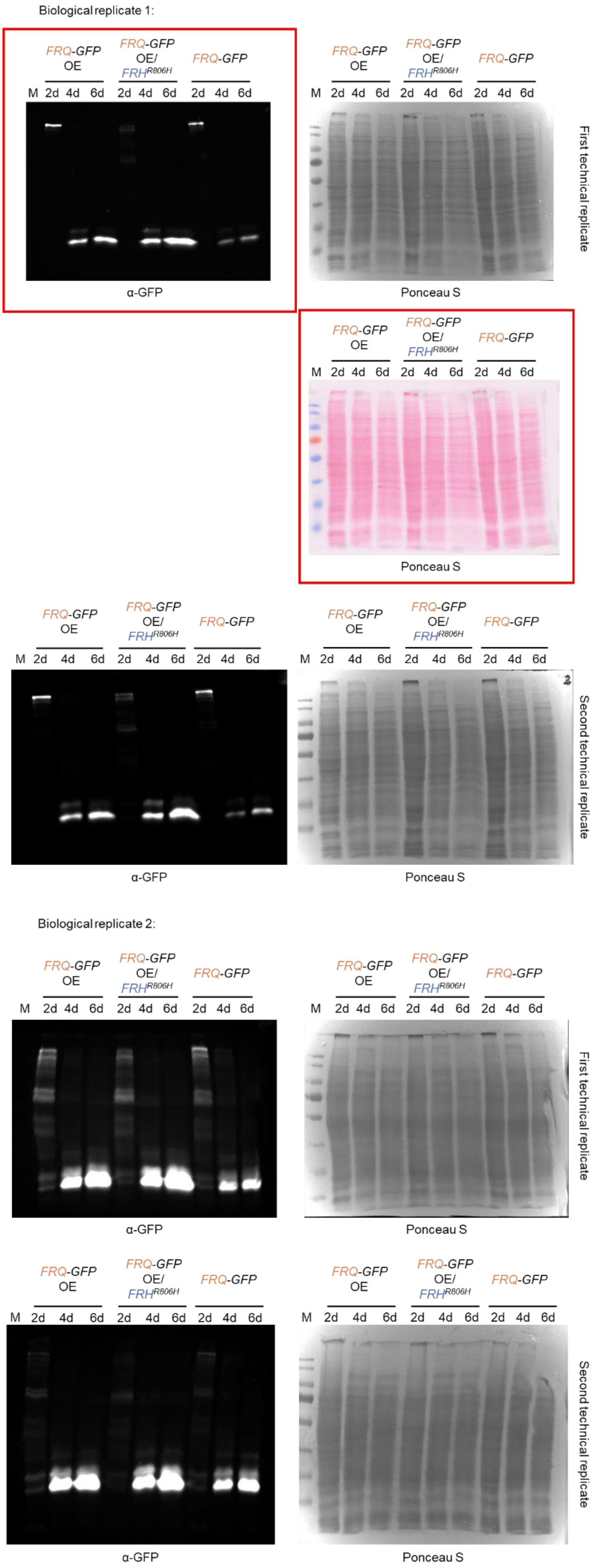
Biological replicate 1:
FRQ-GFP OE
FRQ-GFP OE/ FRH^R806H
FRQ-GFP
M 2d 4d 6d 2d 4d 6d 2d 4d 6d
α-GFP
Ponceau S
First technical replicate
Second technical replicate
Biological replicate 2:
First technical replicate
Second technical replicate

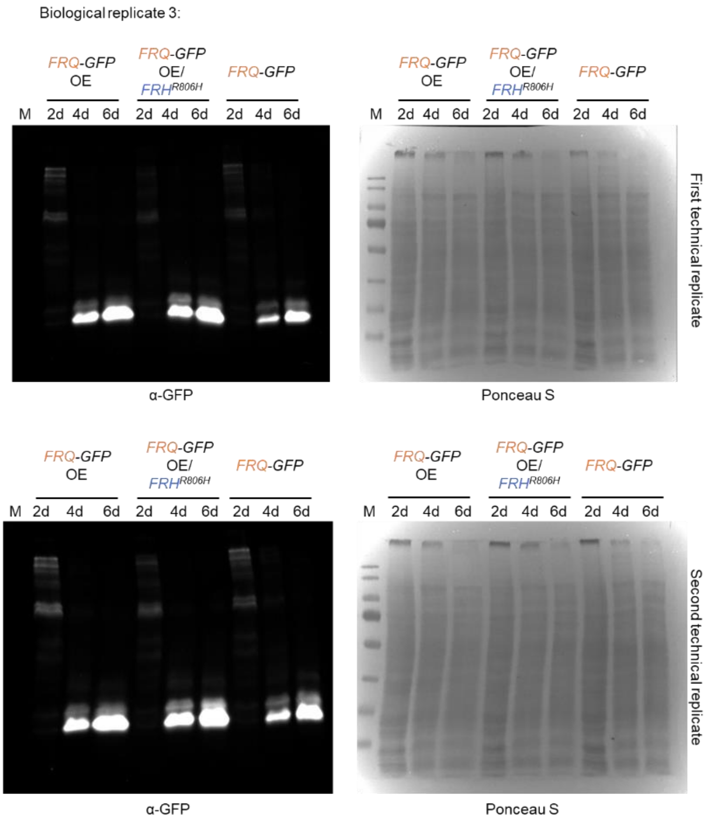

Images Figure S11d. Immunoblot of *V. dahliae* strains expressing *FRQ-GFP* at the endogenous locus under control of the native promoter (*FRQ-GFP*) or under control of the *gpdA* promoter either in presence of wild-type *FRH* (*FRQ-GFP* OE) or point mutated *FRH* (*FRQ-GFP* OE/*FRH*^{R806H}). *V. dahliae* wild-type (WT) served as negative control. Strains were cultivated on SXM agar covered with a nylon membrane for two, four and six days (2d, 4d, 6d) in the light. M: PageRuler Prestained Protein Ladder 10–180 kDa (Thermo Fisher Scientific). Depicted are the three biological replicates with the respective two technical replicates used for the quantification. The grey Ponceau S images were used for normalization and the colored Ponceau S image was used for the figure. The red boxes mark the images used for the figure.

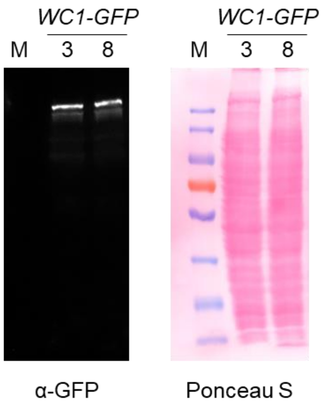

Images Figure S12d. Immunoblot of *V. dahliae* strains expressing *WC1-GFP* at the endogenous locus. Proteins were extracted after three days of cultivation in liquid PDM in the light. The construct for fusion protein expression was not correctly integrated in transformant number three. It was further worked with transformant eight. M: PageRuler Prestained Protein Ladder 10–180 kDa (Thermo Fisher Scientific).

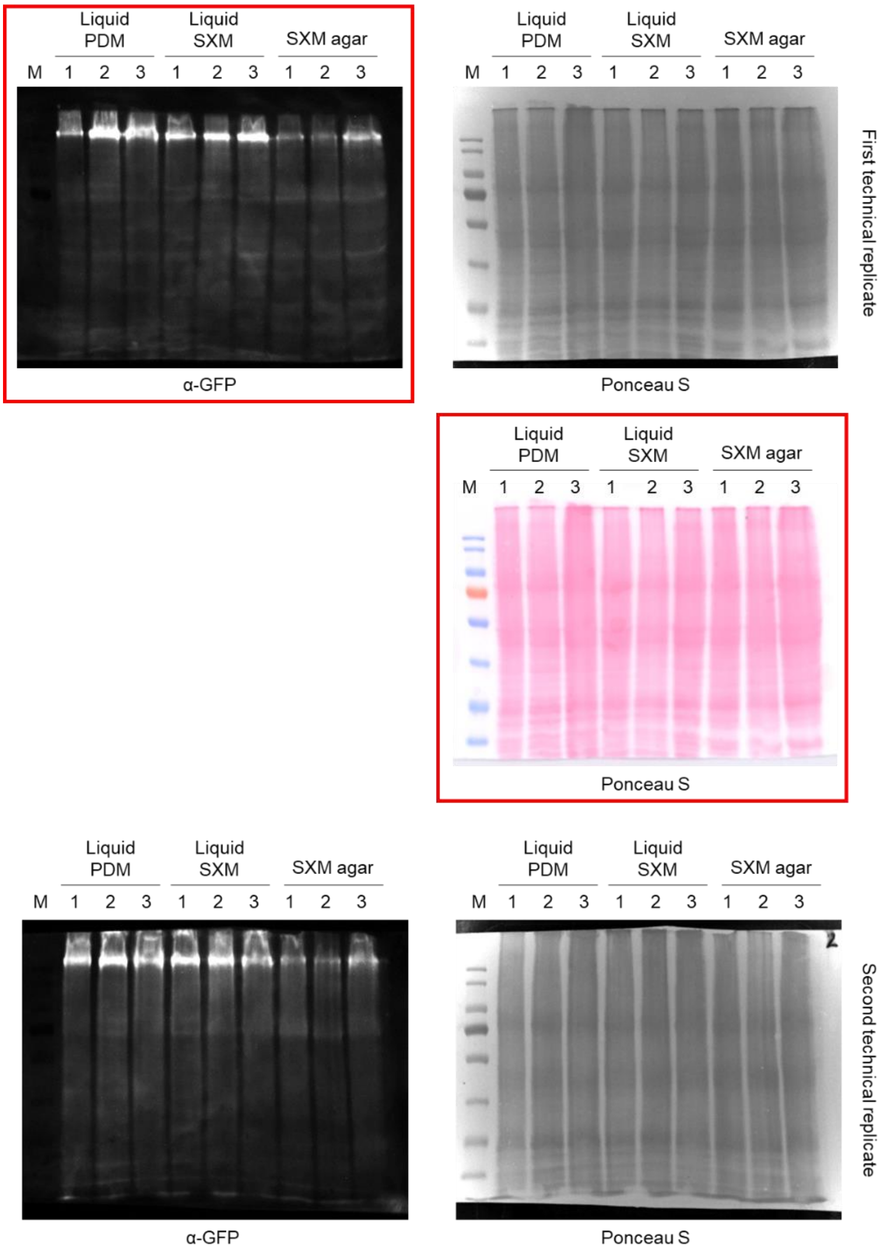

Images Figure S14a. Immunoblot of a *V. dahliae* strain expressing a *FRQ-GFP* fusion construct (*FRQ-GFP*). Strains were either cultivated in liquid SXM or on SXM agar covered with a nylon membrane for two days in the light. Cultivation in liquid PDM served as a control. Three biological replicates of each condition were analyzed (1 – 3). The grey Ponceau S images were used for normalization and the colored Ponceau S image was used for the figure. The red boxes mark the images used for the figure. M: PageRuler Prestained Protein Ladder 10–180 kDa (Thermo Fisher Scientific).

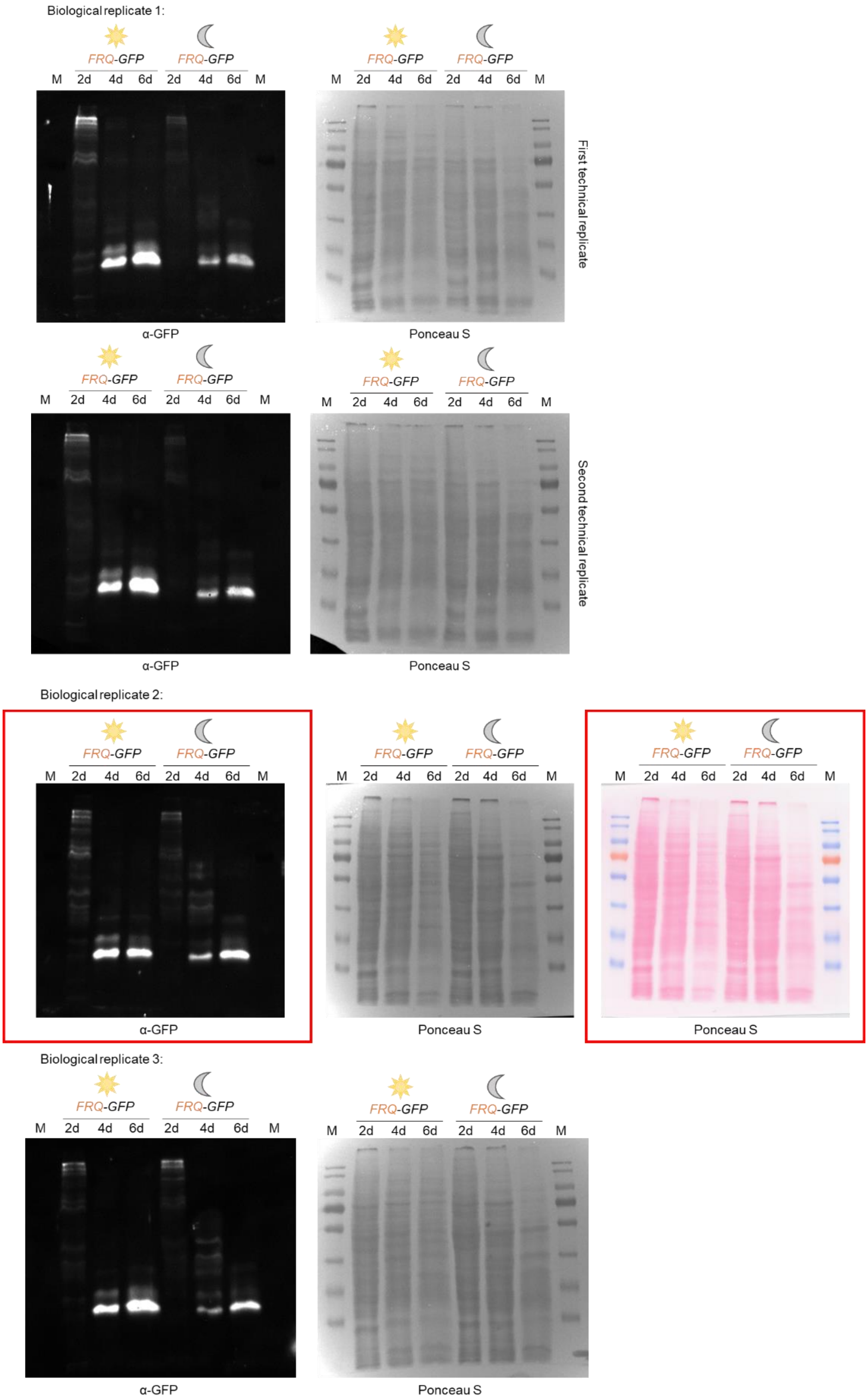

Images Figure S14c. Immunoblot of a *V. dahliae* strain with the *FRQ-GFP* construct at the endogenous locus (*FRQ-GFP*). Strains were grown for two, four, and six days (2d, 4d, 6d) on SXM agar covered with a nylon membrane incubated either in light (sun) or darkness (moon). Three biological replicates were analyzed. The grey Ponceau S images were used for normalization and the colored Ponceau S image was used for the figure. The red boxes mark the images used for the figure. M: PageRuler Prestained Protein Ladder 10–180 kDa (Thermo Fisher Scientific).

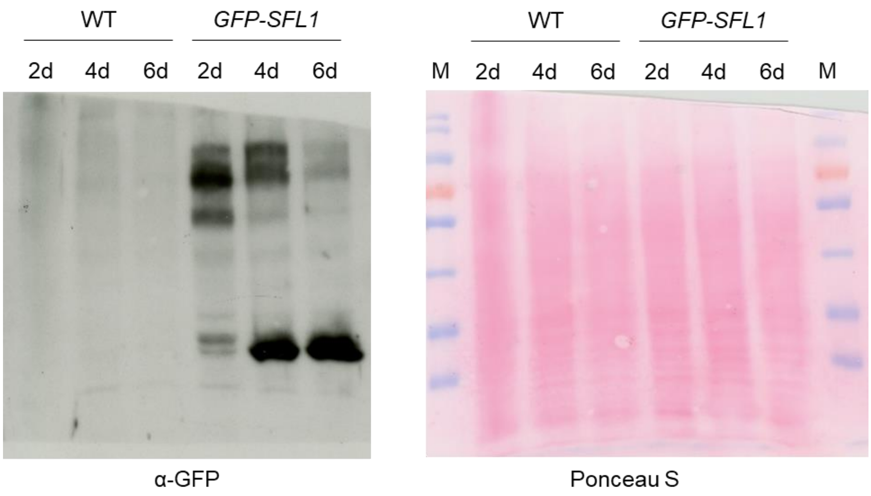

Images Figure S16b. Immunoblot of a *V. dahliae* strain expressing the *GFP-SFL1* construct at the endogenous locus (*GFP-SFL1*). Proteins were extracted after two, four, and six days (2d, 4d, 6d) of cultivation on SXM agar covered with a nylon membrane. *V. dahliae* wild-type (WT) served as control. Cultures were incubated in the light. M: PageRuler Prestained Protein Ladder 10–180 kDa (Thermo Fisher Scientific).

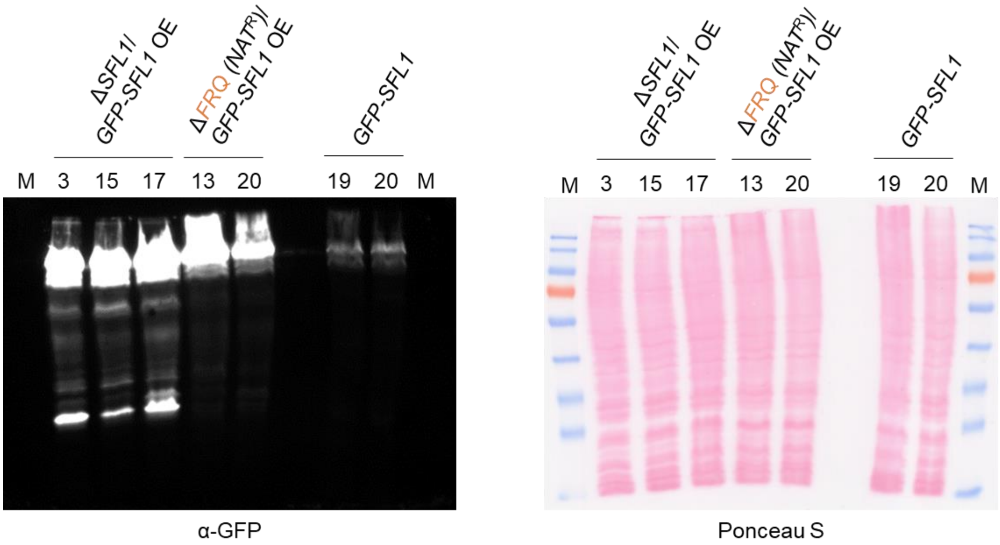

Images Figure S17e. Immunoblot of *V. dahliae SFL1* and *FRQ* deletion strains ectopically overexpressing the *GFP-SFL1* construct (Δ*SFL1*/*GFP-SFL1* OE; Δ*FRQ*/*GFP-SFL1* OE) and a stain expressing the *GFP-SFL1* fusion construct at the endogenous locus (*GFP-SFL1*). Strains were cultivated in liquid PDM for three days in the light. M: PageRuler Prestained Protein Ladder 10–180 kDa (Thermo Fisher Scientific).
