# Supplementary material for: The Frq–Frh Complex Light-Dependently Delays Sfl1-Induced Microsclerotia Formation in Verticillium dahliae
Source: J Fungi (Basel). 2023 Jul 4;9(7):725. doi: 10.3390/jof9070725 (PMC10381341; doi:10.3390/jof9070725)
Supplement: Supplementary file 1 [file jof-09-00725-s001.zip › Supplementary_material.pdf]

Supplementary materials

Supplementary methods

Methods S1

Construction of plasmids with *trpC* promoter-controlled resistance cassettes

The *trpC* promoter-controlled resistance cassettes were initially constructed for deletion of different genes (*VDAG_JR2_Chr7g03380a* and *VDAG_JR2_Chr4g09270a*). A fragment containing a nourseothricin resistance marker (NAT^R) with *trpC* terminator (1,311 bp) was amplified from pME4815 [60] using AN46/AN47 for the construction of a plasmid with a NAT^R cassette controlled by the *trpC* promoter. The *trpC* promoter (371 bp) was obtained from pME4564 [60] with AN16/AN45. The primer AN16 was used to introduce a *Stu*I restriction site (AGGCCT) upstream of the *trpC* promoter. The 3' flanking region of *VDAG_JR2_Chr7g03380a* (1,629 bp) was amplified from *V. dahliae* JR2 wild-type (WT) gDNA with AN43/AN44. Ligation of these three fragments into pME4548 [61] cut with *Eco*RV and *Stu*I (6,804 bp) resulted in pME5489.

A plasmid containing a hygromycin resistance marker (HYG^R) cassette controlled by the *trpC* promoter was constructed by amplification of the *trpC* promoter followed by HYG^R (1,397 bp) from pME4564 [60] using AN16/AN17. The *trpC* terminator (741 bp) was obtained by amplification from pME4815 [60] with AN15/ML8. Together with the 3' flanking region of *VDAG_JR2_Chr4g09270a* (1,176 bp), amplified from WT gDNA using AN5/AN6, these fragments were ligated with pME4548 [61] cut with *Eco*RV and *Stu*I (6,804 bp). The resulting plasmid was named pME5490.

Construction of the *FRQ* deletion (NAT^R) and ectopic complementation strains

A plasmid containing a *FRQ* (*VDAG_JR2_Chr1g01960a*) deletion cassette with NAT^R was constructed. The NAT^R cassette (2,194 bp) was amplified from pME4815 [60] using ML8/ML9. *FRQ* 5' and 3' flanking regions were obtained through amplification from WT gDNA with RH631/RH632 (1,425 bp) and RH633/RH634 (866 bp), respectively. Restriction enzymes *Stu*I and *Eco*RV were used to cut pME4564 [60] and thereby receive the vector backbone (6,804 bp), to which the three fragments were ligated. The resulting plasmid was named pME4879. The *FRQ* deletion (NAT^R) strain VGB296 was obtained through transformation of the WT with this plasmid. Correct transformation was verified via Southern hybridization of gDNA cut with *Sac*II and the 3' flanking region of *FRQ* as probe (Figure S1). The *FRQ* deletion strain with NAT^R cassette is referred to as Δ*FRQ* (NAT^R).

To achieve an ectopic complementation, the *FRQ* ORF as well as the 5' and 3' flanking regions were amplified from WT gDNA with RH631/ML67 (5,296 bp). The pME4880 plasmid was constructed through ligation of this fragment into pPK2 (10,751 bp; [109]), which was linearized with *Eco*RV. Transformation of the *FRQ* deletion strain VGB296 (Δ*FRQ* (NAT^R)) with pME4880 resulted in the ectopic complementation strain VGB411. The ectopic integration of the construct was verified by Southern hybridization with the same enzyme and probe as for the deletion transformant (Figure S1).

Construction of *FRQ* deletion (HYG^R) and ectopic complementation strains

A *FRQ* deletion strain with HYG^R cassette was constructed. The HYG^R cassette was amplified from pPK2 [109] with RO3/ML8 (3,942 bp), and the *FRQ* 5' and 3' flanking regions were amplified from *V. dahliae* WT gDNA using RH631/ML79 (1,425 bp) and RH633/RH634 (866 bp), respectively. The three fragments were ligated with the vector backbone pME4564

[60], cut with *Stu*I and *Eco*RV (6,804 bp). The resulting plasmid pME4881 was used for transformation of the *V. dahliae* WT. The obtained *FRQ* deletion strains VGB402 and VGB403 were verified via Southern hybridization (Figure S1). For this, gDNA was cut with *Bam*HI, and the 5' flanking region of *FRQ* was used as probe (1,425 bp amplified with RH631/ML79). The *FRQ* deletion strains with HYG^R cassette are referred to as Δ*FRQ*.

A *FRQ* complementation strain was constructed through ectopic reintegration of *FRQ*. Plasmid pME4815 (8,928 bp; [60]) was linearized using *Eco*RV and ligated with a fragment consisting of the *FRQ* ORF with 5' and 3' flanking regions, amplified with ML114/ML115 (5,296 bp) from WT gDNA. The resulting plasmid pME4882 was used for transformation of VGB402 and the obtained *FRQ* ectopic complementation transformants were named VGB441 and VGB442. Southern hybridization was performed to verify transformants (Figure S1) using gDNA cut with *Nco*I and the 3' flanking region of *FRQ* as probe (866 bp amplified with RH633/RH634)

<u>Construction of a strain expressing *FRQ–GFP* under *gpdA* promoter control at the endogenous locus</u>

The construct containing *FRQ–GFP* gDNA under control of the *gpdA* promoter was, together with the NAT^R cassette, introduced into the *FRQ* deletion strain VGB402, to allow production of Frq protein that is C-terminally tagged with GFP. For this, the vector backbone containing the 3' and 5' flanking regions of *FRQ* and a *gpdA* promoter (9,978 bp) was amplified from pME4879 with RH633/ML31. The NAT^R cassette (1,682 bp) was amplified from pME5489 using AN56/ML8, and the gene encoding *FRQ* (3,005 bp) was amplified from *V. dahliae* WT gDNA using AN55/ML61. The fragments were ligated, and the resulting plasmid was named pME5491. Next, pME5491 was used as the vector backbone for the final *FRQ–GFP* overexpression plasmid. For this, the whole sequence of pME5491 (14,662 bp) was amplified with AN30/AN58. *GFP* (without start codon) with a flexible linker (protein sequence GGSGG) was amplified with AO165/AN23 (732 bp) from pME5072 [70]. Both fragments were ligated resulting in the final *FRQ–GFP* overexpression plasmid pME5492, which was used for transformation of the *FRQ* deletion strain VGB402. The resulting transformants VGB516 and VGB517 were verified via Southern hybridization (Figure S2) of gDNA cut with *Bam*HI and the 5' flanking region of *FRQ* as probe (1,425 bp amplified with RH631/RH632).

<u>Construction of an in locus *FRQ* complementation strain</u>

For the construction of the in locus *FRQ* complementation strain, a fragment consisting of the NAT^R cassette, 3' and 5' flanking regions of *FRQ* and vector backbone was amplified from pME5491 with AN56/AN57 (10,777 bp). The *FRQ* gene (3,005 bp) was amplified from WT gDNA using ML60/ML61. Ligation of these two fragments resulted in pME5493. Transformation of the *FRQ* deletion (VGB402) with this plasmid resulted in the *FRQ* complementation strains VGB507 and VGB508. Correct integration was verified through Southern hybridization with the same enzyme and probe as described for the *FRQ–GFP* overexpression strain (Figure S2).

<u>Construction of strains with *FRQ–GFP* at the endogenous locus with and without *RFP–H2B*</u>

A *FRQ–GFP*-expressing strain was constructed by amplification of the whole sequence of pME5493 (NAT^R cassette, 3' and 5' flanking regions of *FRQ*, *FRQ* and vector backbone; 13,782 bp) using AN30/AN58. *GFP* (without start codon) with a flexible linker was amplified with AO165/AN23 (732 bp) from pME5072 [70]. Both fragments were ligated, and the resulting plasmid was named pME5494. *V. dahliae* strains VGB353 and VGB354 were obtained by transformation of this plasmid into VGB402. Correct integration of the construct was verified via Southern hybridization using the 5' flanking region as probe (1,425 bp amplified with RH631/RH632) on gDNA cut with *Bam*HI (Figure S2).

In order to analyze subcellular localization of the Frq–GFP fusion protein, a strain expressing *FRQ–GFP* as well as *RFP* fused to histone *H2B* (*VDAG_JR2_Chr2g01720a*) was constructed. *RFP–H2B* expression allows detection of RFP fluorescence in nuclei. Transformation of the *FRQ–GFP*-expressing strain VGB354 with pME4976 [69] resulted in VGB539 and VGB540. Red fluorescence of the transformants was analyzed through microscopy. Correct *FRQ–GFP* strain background was verified through Southern hybridization of gDNA cut with *Pvu*I and the 3' flanking region of *FRQ* (866 bp amplified with RH633/RH634) as probe (Figure S2).

Construction of *WC1* deletion and complementation strains

For the construction of a *WC1* (*VDAG_JR2_Chr2g01990a*) deletion strain, pME4548 [61] was cut with *Eco*RV and *Stu*I to serve as vector backbone (6,804 bp). The 5' flanking region (1,500 bp) was amplified from gDNA with AN80/AN81, the geneticin resistance marker (*GEN*^{R}) cassette was obtained from pCOM [110] as previously described and the 3' flanking region (1,000 bp) was amplified from gDNA using AN82/AN83. Ligation of the three fragments and the vector backbone resulted in pME5501. VGB630 and VGB631 were obtained through transformation of WT using pME5501. Southern hybridization of gDNA cut with *Pst*I and the 3' flanking region as probe was conducted to verify the deletion transformants (Figure S3).

A *WC1* complementation strain was obtained by using the same vector backbone as described above for pME5501. The 5' flanking region and *WC1* gDNA (5,091 bp) were amplified from gDNA using AN80/AN77, and primers AN91/AN83 were used to amplify the 3' flanking region (1,000 bp) from WT gDNA. AN93/ML8 were used for amplification of the *NAT*^{R} cassette (1,682 bp) from pME5494. These three fragments were ligated with the vector backbone to obtain pME5502. The *WC1* deletion strain VGB630 was transformed using this plasmid resulting in complementation transformants VGB664 and VGB665. Verification via Southern hybridization was conducted using the same restriction enzyme and probe as described for the *WC1* deletion strain (Figure S3).

Construction of a strain with *WC1–GFP* at the endogenous locus

A strain producing the Wc1–GFP fusion protein was constructed using pME4548 [61] cut with *Eco*RV and *Stu*I as vector backbone (6,804 bp). The 5' flanking region and *WC1* gDNA without the stop codon (5,088 bp) were amplified from gDNA with AN80/AN111. A fragment including *GFP* (with linker and without start codon) and the *NAT*^{R} cassette (2,417 bp) was amplified from pME5494 using AO165/AN47. The *WC1* 3' flanking region (1,000 bp) was amplified from gDNA using AN91/AN83. Ligation of the three fragments and the vector backbone resulted in pME5503. This plasmid was used for transformation of the *WC1* deletion strain VGB630 to obtain transformant VGB667. The correct integration of the *WC1–GFP* construct was confirmed via Southern hybridization of gDNA cut with *Pst*I and the 3' flanking region as probe (Figure S3; transformant #8). Fluorescence microscopy and a western experiment were performed to check for fusion protein production (Figure S12c and d).

Construction of *FRH*^{R806H} point mutation (*FRH*^{R806H}) and complementation strains

The plasmid needed for the generation of an *FRH* (*VDAG_JR2_Chr4g00070aa*) point mutation strain (*FRH*^{R806H}) was constructed in two steps. First, the *FRH* 3' flanking region (1,350 bp) was amplified from WT gDNA using AN59/AN60 and ligated with pME4548 [61] cut with *Eco*RV and *Stu*I (6,804 bp), resulting in pME5495. Next, the 5' flanking region and a part of *FRH* gDNA (base 1-2,480) were amplified with primers AN61/AN62 (2,706 bp). The point mutation at position 2,481 in the *FRH* gDNA, incorporation of an adenine (A) instead of guanine (G), was created by amplification of the remaining *FRH* gDNA (base 2,482 to end; 953 bp) with AN63/AN64. Both fragments were amplified from WT gDNA. The *HYG*^{R} cassette (2,138 bp) was amplified from pME5490 using AN65/AN66. The three fragments were ligated with

pME5495 (8,160 bp), which was amplified using AN69/AN68. The obtained plasmid was named pME5496. Strains resulting from transformation of the *V. dahliae* JR2 WT, *FRQ–GFP*-expressing (VGB354), or *FRQ–GFP*-overexpressing strain (VGB517) with pME5496 were named VGB541 and VGB542 (*FRH*^{R806H}), VGB543 and VGB544 (*FRQ–GFP*/*FRH*^{R806H}), and VGB545 and VGB546 (*FRQ–GFP* OE/*FRH*^{R806H}), respectively. These transformants were verified via Southern hybridization of gDNA cut with *Xho*I and the *FRH* 3' flanking region as probe (Figure S4). The correct background was also verified for the double mutant strains using gDNA cut with *Pvu*I and the *FRQ* 3' flanking region as probe (866 bp amplified with RH633/RH634) in Southern hybridization (Figure S2). Presence of the *FRH*^{R806H} point mutation in *V. dahliae* transformants was confirmed through sequencing of the point mutated region, which was amplified from mutant gDNA with AN70/71.
The vector backbone for the *FRH* complementation including the *FRH* 3' flanking region was amplified from pME5495 (8,160 bp) using AN73/AN68. Amplification of *FRH* 5' flanking region and *FRH* gDNA (3,660 bp) from gDNA was conducted with AN61/AN74, and the *GEN*^{R} cassette (1,648 bp) was amplified from pCOM [110] with JST253/JST254. Ligation of these three fragments resulted in pME5497. The *FRH* gDNA contained a silent base exchange (C to T) at base 1,240. Transformation of the *FRH*^{R806H} strain (VGB541) using pME5497 resulted in complementation transformants VGB577 and VGB578. Correct integration of the construct was verified via Southern hybridization using the same enzyme and probe as for the *FRH*^{R806H} point mutation strains (Figure S4).

Construction of a strain with *FRH–GFP* at the endogenous locus

To construct a strain producing an Frh–GFP fusion protein, AN114/AN115 were used to amplify the vector backbone including the *FRH* 5' flanking region, *FRH* gDNA without the stop codon and *FRH* 3' flanking region (11,814 bp) from pME5497. The insert consisting of *GFP* (with linker, without start codon) and the *NAT*^{R} cassette (2,417 bp) were amplified from pME5494 using AO165/AN47. Ligation of this fragment with the vector backbone resulted in pME5498. This plasmid was used for transformation of VGB541, and the resulting transformants were named VGB695 and VGB696. The *FRH* gDNA contained a silent base exchange (C to T) at base 1,240. Southern hybridization of gDNA cut with *Sma*I and the 3' flanking region as probe (1,350 bp amplified using AN59/AN60) was conducted to verify the new strains (Figure S4).

Construction of a strain with *FRH*^{R806H}*–GFP* at the endogenous locus

A strain with GFP fused to the C-terminus of Frh^{R806H} was constructed by amplification of *GFP* (with linker, without start codon) and the *NAT*^{R} cassette (2,417 bp) from pME5494 with AO165/AN47. This fragment was ligated with the vector backbone obtained through amplification of the *FRH* 5' flanking region, *FRH*^{R806H} gDNA without the stop codon and *FRH* 3' flanking region (11,814 bp) from pME5496 with AN114/AN115, resulting in pME5499. The strains obtained through transformation of VGB541 with pME5499 were named VGB697and VGB698. Correct integration of the construct was verified through Southern hybridization as described for the *FRH–GFP* strain (Figure S4).

Construction of an *FRH* deletion plasmid

It was attempted to construct an *FRH* deletion strain by amplification of the 5' flanking region (1,158 bp) from gDNA with AN116/AN117. A fragment consisting of the *HYG*^{R} cassette and 3' flanking region (3,491 bp) was amplified from pME5496 with AN118/AN60. The two fragments were ligated to pME4548 [61] cut with *Eco*RV and *Stu*I (6,804 bp). The resulting plasmid was named pME5500. Transformation of the WT with this plasmid has so far been unsuccessful.

Construction of the ectopic *GFP–SFL1* overexpression strains

The construct allowing the production of N-terminally GFP-tagged Sfl1 (VDAG_JR2_Chr4g02790a) was ectopically overexpressed in the *SFL1* deletion strain (VGB324). A fragment containing the *gpdA* promoter and *GFP* (without the stop codon) was amplified from pGreen2 [67] using RH635/ZQY11 (1,609 bp). The primer ZQY11 includes a sequence coding for a flexible linker (protein sequence GGSGG). The primers ML73/RH665 were used for amplification of the *SFL1* ORF (2,069 bp) from WT gDNA, and the *trpC* terminator was amplified from pGreen2 [67] with ML74/RH636 (772 bp). Both fragments, the *SFL1* ORF and the *trpC* terminator, were fused by PCR using ML73/RH636. The resulting two fragments were ligated to pPK2 [109] (linearized with *Eco*RV, 10,751 bp), producing pME4876. This plasmid was used for transformation of VGB324 [61], resulting in *GFP–SFL1*-overexpressing transformants VGB266, VGB348, and VGB349. Ectopic integration of the construct was verified by Southern hybridization (Figure S5) using genomic DNA cut by *Pst*I and a part of the *SFL1* gene (664 bp, amplified with RH664/RH668) as probe.

Construction of a *GFP–SFL1* complementation strain

The *SFL1* deletion strain VGB324 [61] served as background strain for *GFP–SFL1* integration at the endogenous locus. The vector backbone including the 5' and 3' flanking regions of *SFL1* was amplified from pME4727 (9,101 bp; [61]) using RH660/ML77, the *GFP–SFL1* fusion construct was amplified from pME4876 with ZQY10/ML78 (3,942 bp), and the HYG^R cassette was amplified from pPK2 [109] using RO3/ML8 (3,942 bp). Ligation of all fragments resulted in pME4877. Transformation of VGB324 [61] with this plasmid resulted in the strains VGB433 and VGB434. Correct integration of the construct was verified by Southern hybridization (Figure S5) using gDNA cut with *Pst*I and the beginning of the *SFL1* ORF as probe (664 bp amplified with RH664/RH668).

Construction of a *WC1* and *FRQ* double-deletion strain

The Δ*WC1*/Δ*FRQ* strain was obtained through transformation of the *FRQ* deletion strain VGB402 using pME5501. The two double-deletion transformants were named VGB699 and VGB700. Southern hybridization with *Nco*I digested gDNA and the *WC1* 3' flanking region as probe (1,000 bp amplified with AN82/AN83) was performed to verify *WC1* deletion (Figure S3).The *FRQ* deletion was confirmed through Southern hybridization using the 5' flanking region of *FRQ* (1,425 bp amplified with RH631/RH632) and gDNA cut with *Bam*HI (Figure S2).

Construction of Δ*WC1*/FRH^{R806H} double mutant strain

Double mutant strains with *WC1* deletion and FRH^{R806H} point mutation were constructed through transformation of the FRH^{R806H} strain VGB541 with pME5501. The resulting transformants were named VGB701 and VGB702. Deletion of *WC1* was verified via Southern hybridization as previously described for the Δ*WC1*/Δ*FRQ* strain (Figure S3). The FRH^{R806H} background was confirmed (Figure S4) using gDNA cut with *Xho*I and 3' flanking region of *FRH* as probe (1,350 bp amplified with AN59/AN60).

Construction of the *FRQ* deletion strain ectopically overexpressing *GFP–SFL1*

The fragment encoding for *GFP–SFL1* under control of the constitutively active *gpdA* promoter was ectopically integrated into VGB296 through transformation with plasmid pME4876. The obtained strains were named VGB435 and VGB436. Ectopic integration was verified by Southern hybridization using gDNA cut by *Pst*I and a part of the *SFL1* gene as probe (664 bp amplified with RH664/RH668, Figure S5).

Construction of Δ*FRQ*/Δ*SFL1* double mutant strains

In a double-deletion mutant, both the *SFL1* and *FRQ* ORFs were deleted. The plasmid pME4881, which contains the *FRQ* deletion cassette (with HYG^R) as described above, was used for transformation of the *SFL1* deletion strain VGB324. The resulting *FRQ/SFL1* double-deletion transformants VGB404 and VGB405 were verified by Southern hybridization of gDNA cut with *Bam*HI and the 5' flanking regions of *FRQ* (1,425 bp amplified with RH631/ML79) and *SFL1* (699 bp amplified with RH667/RH659) as probes (Figure S1).

Supplementary figures

Figure S1

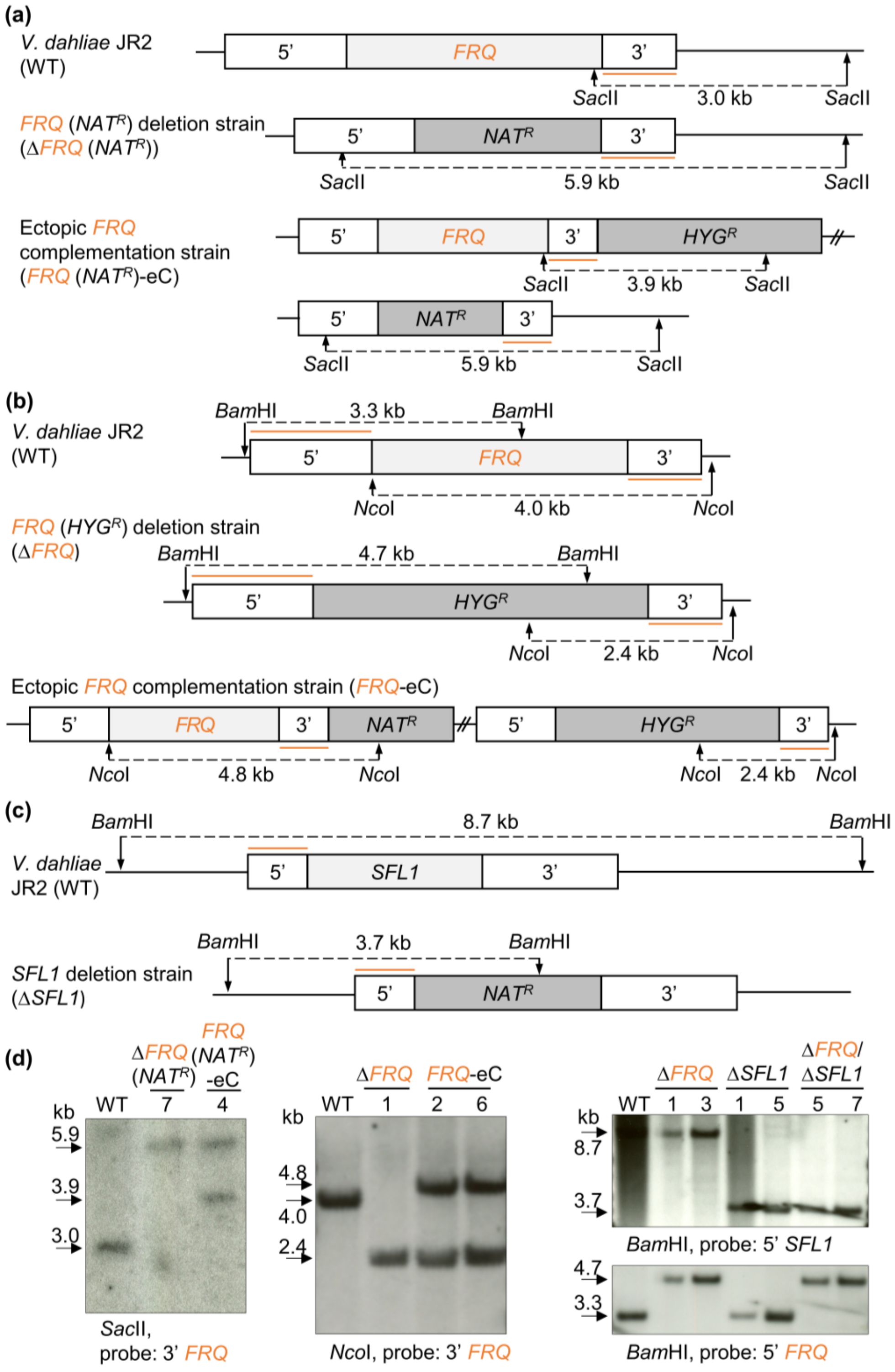

The figure legend is on the next page.

Figure S1. Verification of *V. dahliae FRQ* deletion, ectopic complementation, and *FRQ*/*SFL1* double-deletion strains. **(a-c)** Schemes of *Sac*II, *Nco*I, and *Bam*HI restriction sites for Southern hybridization of *V. dahliae* wild-type (WT) and indicated mutant strains. The *FRQ* open reading frame in the *V. dahliae* JR2 wild-type (WT) was replaced by two different deletion constructs ((Δ*FRQ* (*NAT*^{R}), Δ*FRQ*) via homologous recombination. The *FRQ*/*SFL1* double-deletion (Δ*FRQ*/Δ*SFL1*) was generated through introduction of the *FRQ* deletion construct into the *SFL1* deletion strain (VGB324). The complementation strains (*FRQ (NAT*^{R}*)*-eC, *FRQ*-eC) were obtained by ectopic integration (indicated by //) of the complementation construct into the deletion strains (VGB296, VGB402). The constructs contain either a hygromycin (*HYG*^{R}) or nourseothricin (*NAT*^{R}) resistance cassette under control of the *gpdA* promoter and *trpC* terminator. Restriction sites (arrows) and probes (orange lines) used for Southern hybridizations are depicted. **(a, b)** *FRQ* deletion and ectopic complementation strains were confirmed using the *FRQ* 3' flanking region as probe with gDNA cut by the enzyme **(a)** *Sac*II or **(b)** *Nco*I. **(b, c)** The enzyme *Bam*HI was used with the *FRQ* 5' flanking region or *SFL1* 5' flanking region as probe to confirm the *FRQ*/*SFL1* double-deletion strain. **(d)** Southern hybridizations verify the Δ*FRQ* (*NAT*^{R}) transformant seven (VGB296), *FRQ* (*NAT*^{R})-eC transformant four (VGB411), Δ*FRQ* transformants one and three (VGB402, VGB403), *FRQ*-eC transformants two and six (VGB441, VGB442), and the Δ*FRQ*/Δ*SFL1* transformants five and seven (VGB404, VGB405). Genomic DNA of WT and the Δ*SFL1* single-deletion transformants served as control. Restriction enzymes, probes, and sizes of expected fragments are indicated.

Figure S2

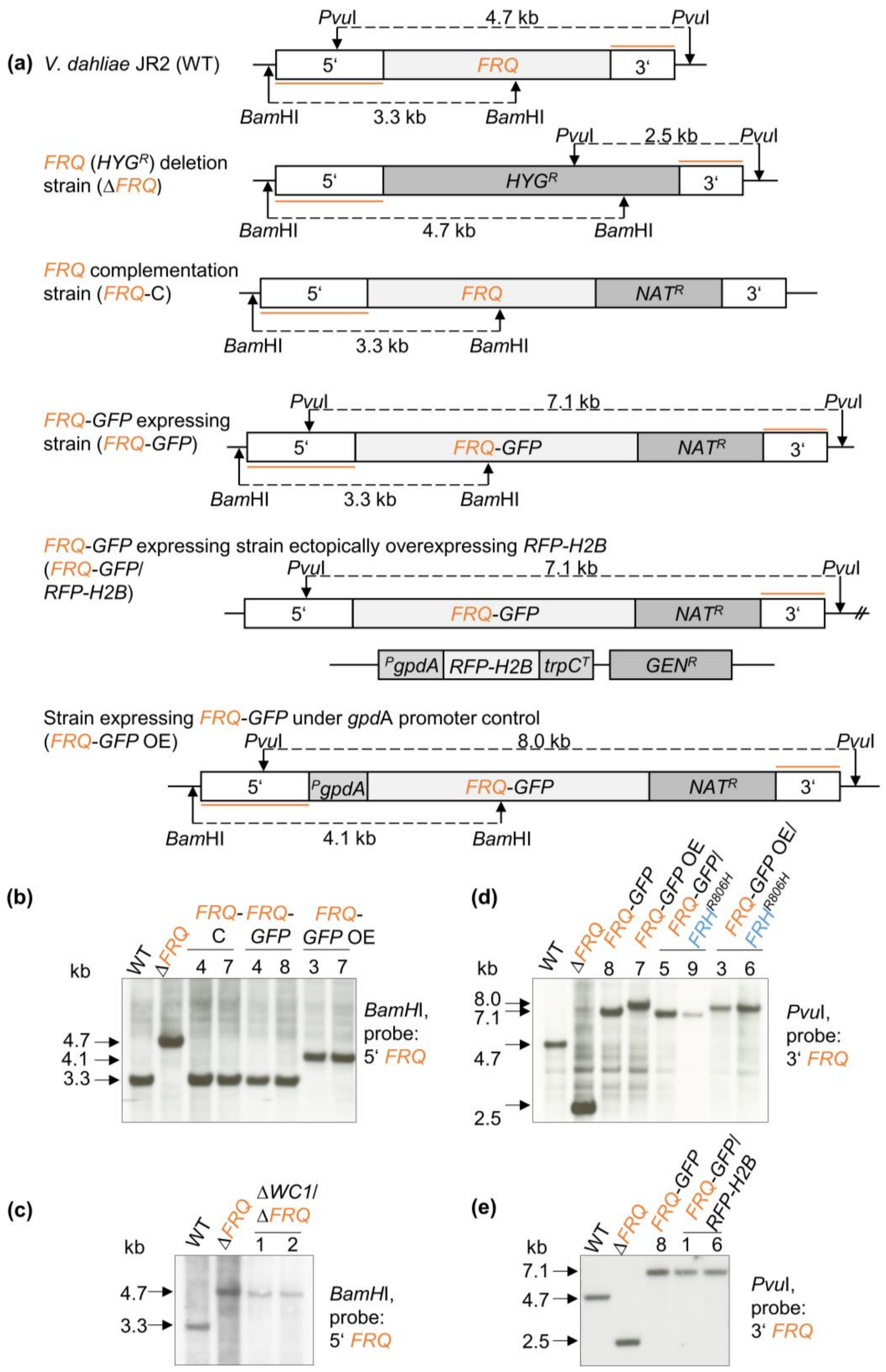

The figure legend is on the next page.

Figure S2. Verification of *V. dahliae FRQ* in locus complementation and *FRQ–GFP*-expressing strains. **(a)** Schemes of *Bam*HI and *Pvu*I restriction sites for Southern hybridization of *V. dahliae* wild-type (WT) and indicated *FRQ* mutant strains. The *FRQ* complementation and *FRQ–GFP* constructs were integrated into the *FRQ* deletion mutant background (Δ*FRQ*) through homologous recombination. The *gpdA* promoter- and *trpC* terminator-controlled hygromycin B resistance marker (HYG^R) of the deletion strain was replaced by *FRQ* followed by the nourseothricin resistance marker (NAT^R) under control of *trpC* promoter and terminator to generate a *FRQ* complementation strain (*FRQ*-C). The constructs for *FRQ–GFP* expression under control of the native promoter (*FRQ–GFP*) or under *gpdA* promoter control for overexpression (*FRQ–GFP* OE) also confer nourseothricin resistance. An *RFP–H2B* fusion construct followed by a *trpC* promoter- and terminator-controlled geneticin G418 resistance cassette (GEN^R) was ectopically integrated (//) into the *FRQ–GFP* strain (*FRQ–GFP*/*RFP–H2B*) for visualization of nuclei. Arrows indicate *Bam*HI and *Pvu*I restriction sites, and fragment sizes resulting from hybridization with either labeled 5‘ or 3‘ flanking region as a probe (orange line) are depicted. **(b-e)** Constructed strains were verified via Southern hybridization of *Bam*HI or *Pvu*I digested DNA using the 5‘ or 3' flanking region of *FRQ* as probe, respectively. Sizes of expected fragments are indicated. The WT and Δ*FRQ* strain (VGB402) served as controls. **(b)** *FRQ*-C transformants four and seven (VGB507, VGB508), *FRQ–GFP* transformants four and eight (VGB353, VGB354), and *FRQ–GFP* OE transformants three and seven (VGB516, VGB517) were correct. **(c)** Correct integration of the *WC1* deletion construct in the *FRQ* deletion background was confirmed for Δ*WC1/*Δ*FRQ* transformants one and two (VGB699, VGB700). **(d)** The *FRQ–GFP* mutant background in double mutants with point mutated *FRH* (*FRQ–GFP*/FRH^{R806H}, *FRQ–GFP* OE/FRH^{R806H}) was verified. *FRQ–GFP* transformant eight and *FRQ–GFP* OE transformant seven served as controls. *FRQ–GFP*/FRH^{R806H} transformants five and nine (VGB543, VGB544) as well as *FRQ–GFP* OE/FRH^{R806H} transformants three and six (VGB545, VGB546) were correct. **(e)** *FRQ–GFP/RFP–H2B* transformants one and six (VGB539, VGB540) were verified. *FRQ–GFP* transformant eight served as a control.

Figure S3

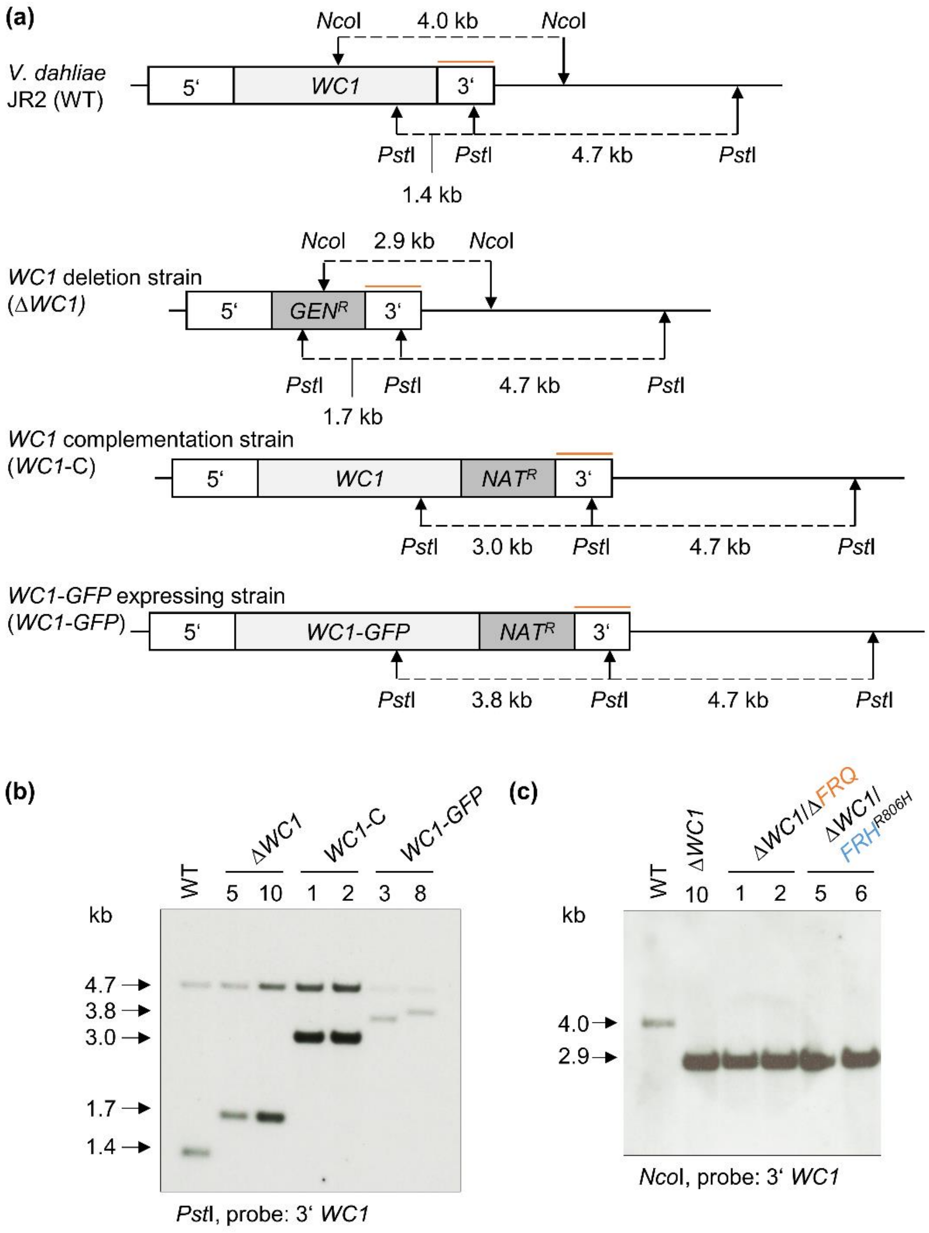

The figure legend is on the next page.

Figure S3. Verification of *WC1* deletion, complementation, and *WC1–GFP*-expressing strains. **(a)** Schematic depiction of *Pst*I and *Nco*I restriction sites for Southern hybridization of *V. dahliae* wild-type (WT), *WC1* deletion (Δ*WC1*), complementation (*WC1*-C), and *WC1–GFP*-expressing strains (*WC1–GFP*). For deletion, the *WC1* gene locus was replaced by a geneticin G418 resistance marker (GEN^{R}) controlled by *trpC* promoter and terminator. *WC1* or *WC1–GFP* were reintroduced together with a nourseothricin resistance marker (NAT^{R}) under *trpC* promoter and terminator control to construct the *WC1* complementation and *WC1–GFP*-expressing strain. Arrows indicate restriction sites of *Pst*I and *Nco*I, and expected fragments by usage of a *WC1* 3'flanking region probe (orange line) are depicted. **(b, c)** Correct construct integration in respective mutant strains was verified through Southern hybridization. Enzymes and probe as well as expected fragment sizes are indicated. *V. dahliae* WT was included as a control. **(b)** For all strains one individual and a common 4.7 kb fragment were detected. Δ*WC1* transformants five and ten (VGB630, VGB631) as well as *WC1*-C transformants one and two (VGB664, VGB665) were correct. The correct signal at 3.8 kb was only detected for the *WC1–GFP* transformant eight (VGB667). The *WC1–GFP* construct in transformant three was not correctly integrated. **(c)** Double mutants with *WC1* deletion and *FRQ* deletion (Δ*WC1*/Δ*FRQ*) or *FRH* point mutation (Δ*WC1*/FRH^{R806H}) were verified through Southern hybridization. Δ*WC1* transformant ten was included as control. Δ*WC1*/Δ*FRQ* transformants one and two (VGB699, VGB700) and Δ*WC1*/FRH^{R806H} transformants five and six (VGB701, VGB702) were correct.

Figure S4

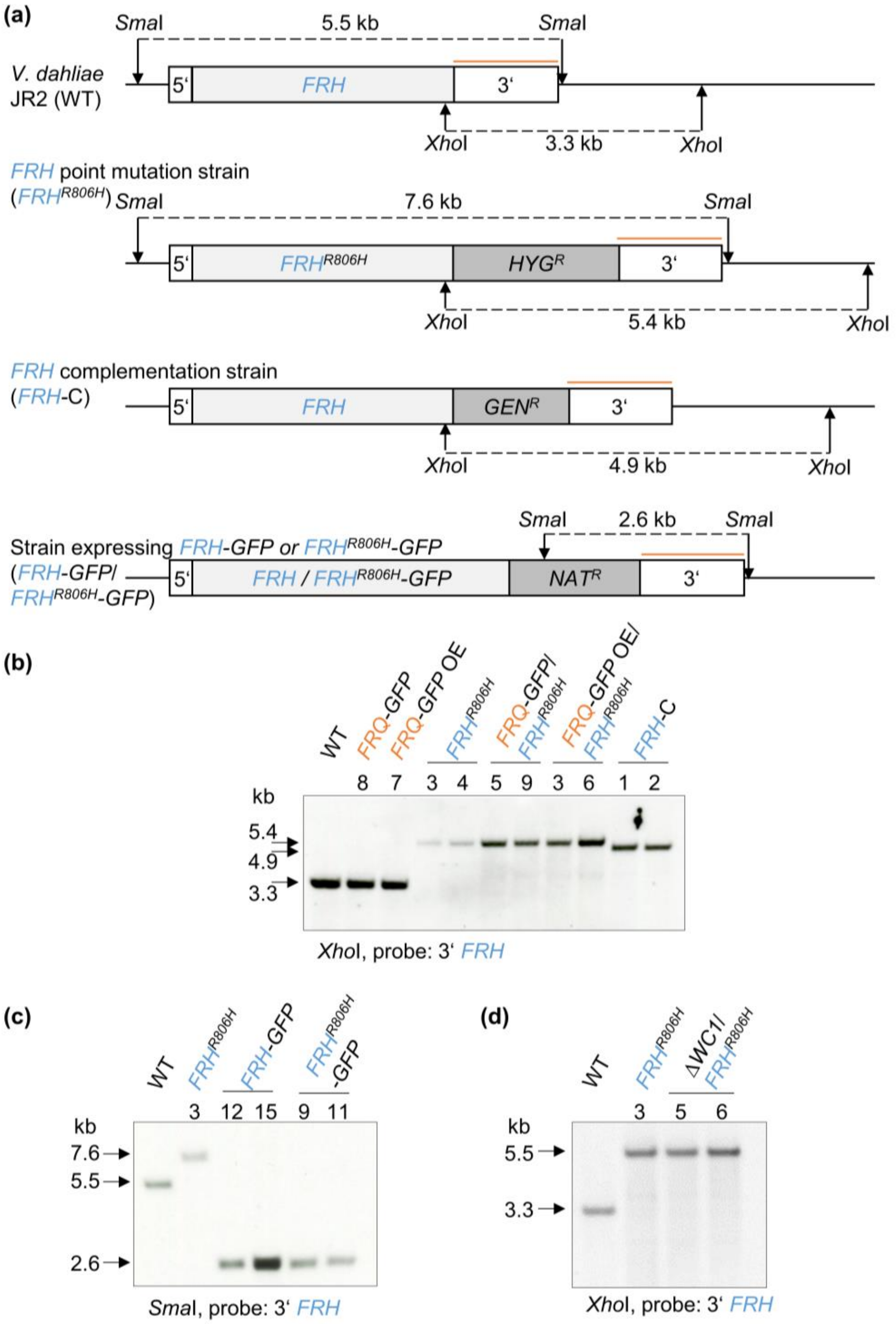

The figure legend is on the next page.

Figure S4. Verification of *FRH* point mutation, complementation, and *FRH–GFP*-expressing strains. **(a)** Scheme of *Xho*I and *Sma*I restriction sites for Southern hybridization of *V. dahliae* wild-type (WT) and indicated *FRH* mutant strains. The resistance markers were controlled by the *trpC* promoter and terminator. The construct for introduction of the *FRH* point mutation additionally contained a hygromycin B resistance marker (*HYG*^{R}). This construct was introduced into *V. dahliae* wild-type (*FRH*^{R806H}) and mutant strains expressing the *FRQ–GFP* construct under native (*FRQ–GFP*/*FRH*^{R806H}) or *gpdA* promoter control (*FRQ–GFP* OE/*FRH*^{R806H}) through homologous recombination. Wild-type *FRH* was reintroduced into the *FRH* point mutation strain (*FRH*^{R806H}) resulting in the *FRH* complementation strain (*FRH*-C) with a geneticin G418 resistance cassette (*GEN*^{R}). *FRH–GFP* and *FRH*^{R806H}*–GFP* fusion constructs with nourseothricin resistance marker (*NAT*^{R}) were integrated into the *FRH*^{R806H} mutant strain (*FRH–GFP*, *FRH*^{R806H}*–GFP*). Restriction sites of *Sma*I and *Xho*I (arrows), as well as resulting fragment sizes (dashed lines) by usage of the *FRH* 3‘ flanking region as probe (orange line) are indicated. **(b-d)** Results of Southern hybridizations are depicted. Restriction enzymes, the probe, and resulting fragment sizes are indicated. **(b)** Constructed *FRH*^{R806H} mutant transformants three and four in WT background (VGB541, VGB542), transformants five and nine in *FRQ–GFP* background (VGB543, VGB544), transformants three and six in *FRQ–GFP* OE background (VGB545, VGB546), and *FRH*-C transformants one and two (VGB577, VGB578) were verified via Southern hybridization. *V. dahliae* WT, *FRQ–GFP* transformant eight (VGB354), and *FRQ–GFP* OE transformant seven (VGB517) were included as controls. **(c)** *FRH–GFP* transformants 12 and 15 (VGB695, VGB696) and *FRH*^{R806H}*–GFP* transformants nine and 11 (VGB697, VGB698) were verified through Southern hybridization. As controls, *V. dahliae* WT and *FRH*^{R806H} transformant three (VGB541) were included. **(d)** Constructed *WC1* deletion and *FRH*^{R806H} double mutant strains (Δ*WC1*/*FRH*^{R806H}) were verified via Southern hybridization of gDNA cut with *Xho*I and 3‘ flanking region of *FRH* as probe. The expected 5.5 kb fragment was detected for Δ*WC1/FRH*^{R806H} transformants five and six (VGB701, VGB702).

Figure S5

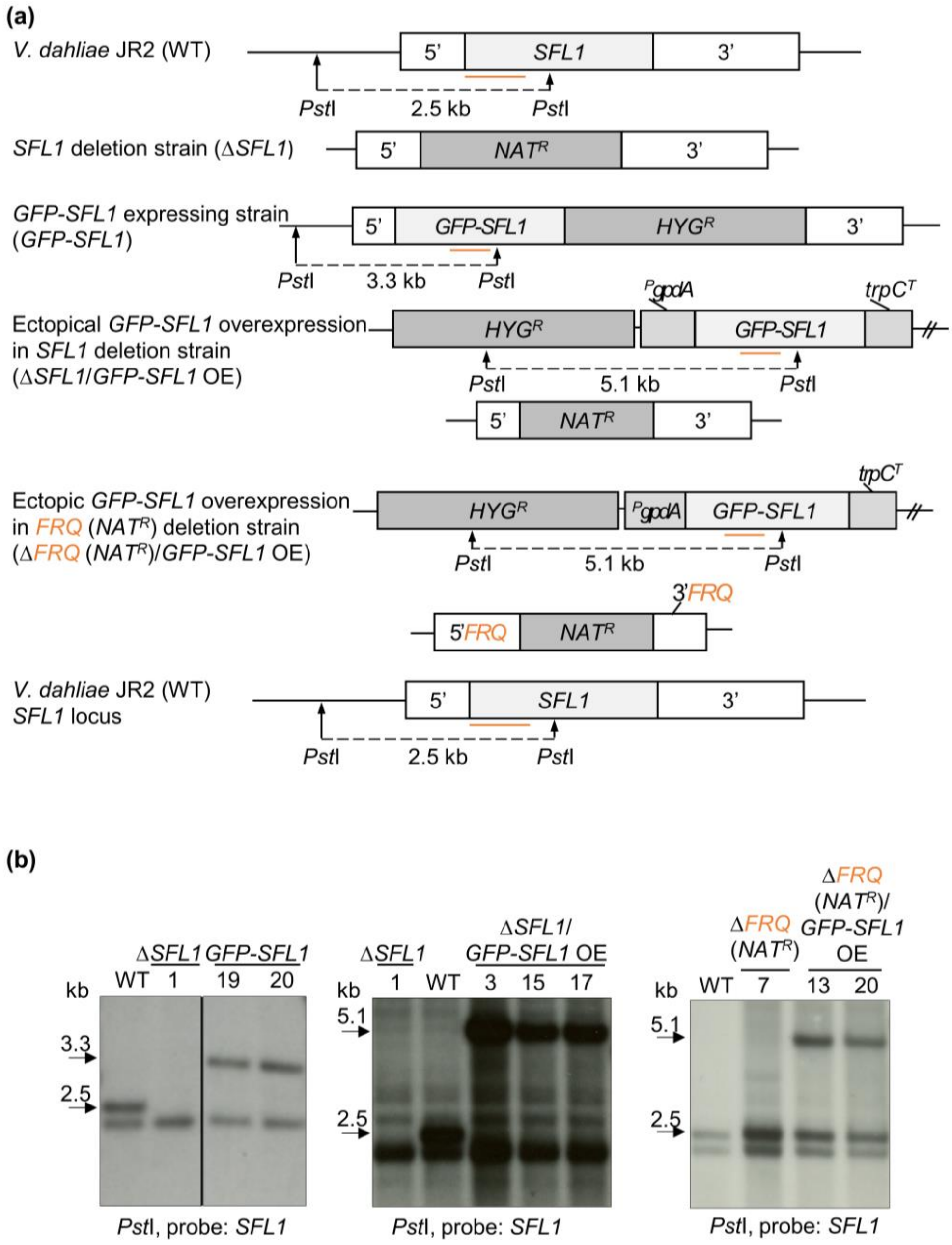

The figure legend is on the next page.

Figure S5. Verification of the *V. dahliae* strains with *GFP–SFL1* in locus or ectopically overexpressed *GFP–SFL1* in either *SFL1* or *FRQ* (*NAT*^{R}) deletion strains. **(a)** Schemes of *Pst*I restriction sites in the *V. dahliae* JR2 wild-type (WT) and indicated mutant strains. Restriction sites (arrows) and probes (orange lines) used for Southern hybridizations are depicted. The *GFP–SFL1* construct (driven by the native promoter and terminator) was integrated into the *SFL1* deletion strain VGB324 (Δ*SFL1*) at the endogenous locus via homologous recombination to generate the *GFP–SFL1*-expressing strain (*GFP–SFL1*). The Δ*SFL1* strain overexpressing *GFP–SFL1* (Δ*SFL1*/*GFP–SFL1* OE) was obtained by ectopical integration (indicated by //) of the construct (controlled by *gpdA* promoter (^{P}*gpdA*) and *trpC* terminator (*trpC*^{T})) into the deletion strain. The construct allowing *GFP–SFL1* overexpression was also ectopically integrated into the *FRQ* deletion strain (Δ*FRQ* (*NAT*^{R})/*GFP–SFL1* OE). The *SFL1* and *FRQ* (*NAT*^{R}) deletion constructs contain a nourseothricin resistance cassette (*NAT*^{R}), whereas the *GFP–SFL1* constructs include a hygromycin resistance cassette (*HYG*^{R}). Both resistance markers are under control of the *gpdA* promoter and *trpC* terminator. The beginning of *SFL1* was used as probe on DNA cut with *Pst*I to verify correct integration of the constructs. **(b)** Results of the Southern hybridizations are depicted. Restriction enzyme, probe, and sizes of expected fragments are indicated. *GFP–SFL1* transformants 19 (VGB433) and 20 (VGB434), Δ*SFL1*/*GFP–SFL1* OE transformants three (VGB266), 15 (VGB348), and 17 (VGB349) as well as Δ*FRQ* (*NAT*^{R})/*GFP–SFL1* OE transformants 13 (VGB435) and 20 (VGB436) were correct. Genomic DNA of WT, Δ*SFL1,* and Δ*FRQ* (*NAT*^{R}) served as controls.

Figure S6

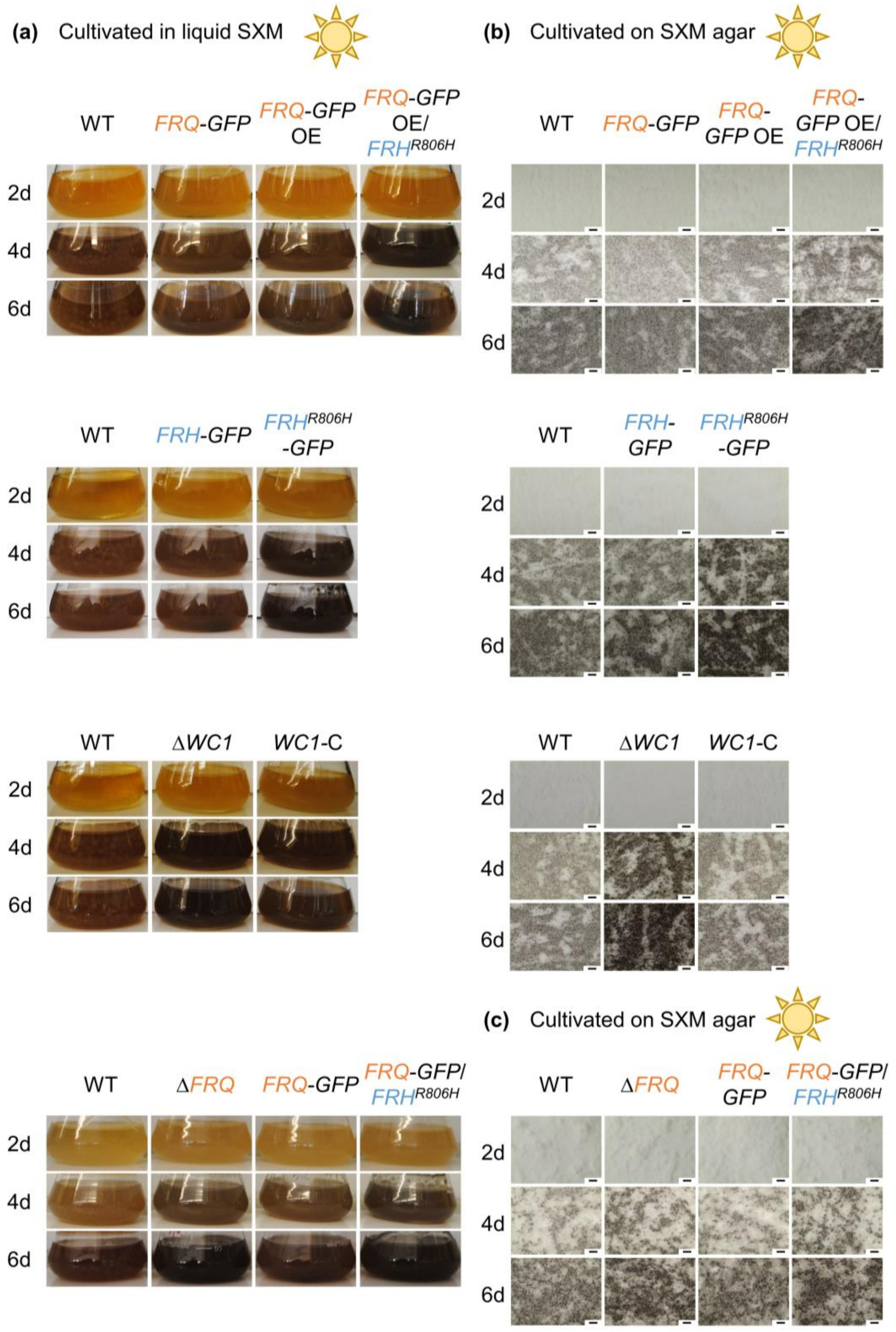

The figure legend is on the next page.

Figure S6. *V. dahliae FRQ* and *WC1* deletion as well as *FRH* point mutation strains melanize stonger than wild-type if cultivated in liquid or on solid SXM. Spores of *V. dahliae* strains were inoculated into **(a)** liquid SXM or **(b, c)** on SXM agar covered with a nylon membrane using **(a, c)** 1×10^6 **(b)** 4×10^6 spores. The following strains were included: Wild-type strain (WT), strains expressing *FRQ–GFP* under control of the *gpdA* promoter with wild-type *FRH* (*FRQ–GFP* OE) or point mutated *FRH* (*FRQ–GFP* OE/*FRH*^{R806H}), *FRQ* deletion strain (Δ*FRQ*), strains expressing *FRQ–GFP* under control of the native promoter with wild-type *FRH* (*FRQ–GFP*) or with point mutated *FRH* (*FRQ–GFP*/*FRH*^{R806H}), strains expressing *FRH–GFP* (*FRH–GFP)* or *FRH*^{R806H}*–GFP* (*FRH*^{R806H}*–GFP*) as well as *WC1* deletion (Δ*WC1*) and complementation (*WC1*-C) strains. Representative pictures of cultures after two (2d), four (4d), and six (6d) days of incubation are depicted. Stronger melanization of *FRQ* deletion, *FRH* point mutation, and *WC1* deletion strains was observed compared with WT under both cultivation conditions. Scale bar: 500 µm.

Figure S7

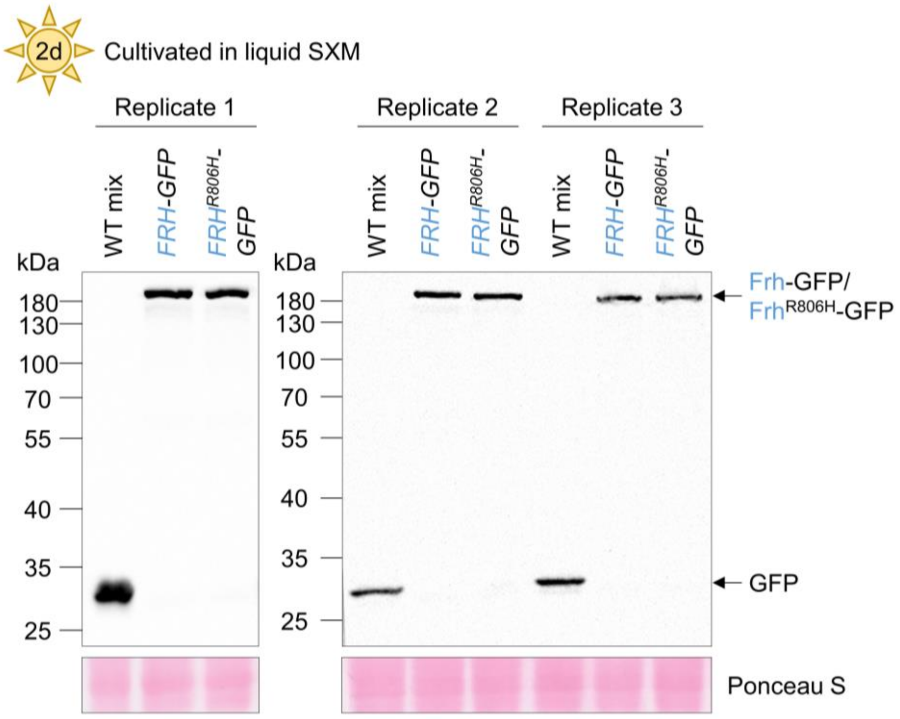

Figure S7. Amount of free GFP in the wild-type control is similar to Frh–GFP and Frh^{R806H}–GFP protein levels. 5 × 10^9 spores of *V. dahliae* wild-type (WT), wild-type ectopically overexpressing *GFP* (WT/*GFP* OE), and strains expressing endogenous levels of the *FRH–GFP* or *FRH^{R806H}–GFP* fusion construct (*FRH–GFP, FRH^{R806H}–GFP*) were inoculated into 500 ml liquid SXM and incubated for two days in the light. Ground mycelium of both wild-type strains was mixed (WT mix; 59/60 WT, 1/60 WT/*GFP* OE). Proteins were extracted and subjected to western experiments with a GFP antibody. Ponceau S staining of the membrane served as loading control. The results of three biological replicates are depicted. Similar amounts of free GFP, Frh–GFP, or Frh^{R806H}–GFP fusion proteins were detected in the WT mix, *FRH–GFP*, or *FRH^{R806H}–GFP* extracts, respectively.

Figure S8

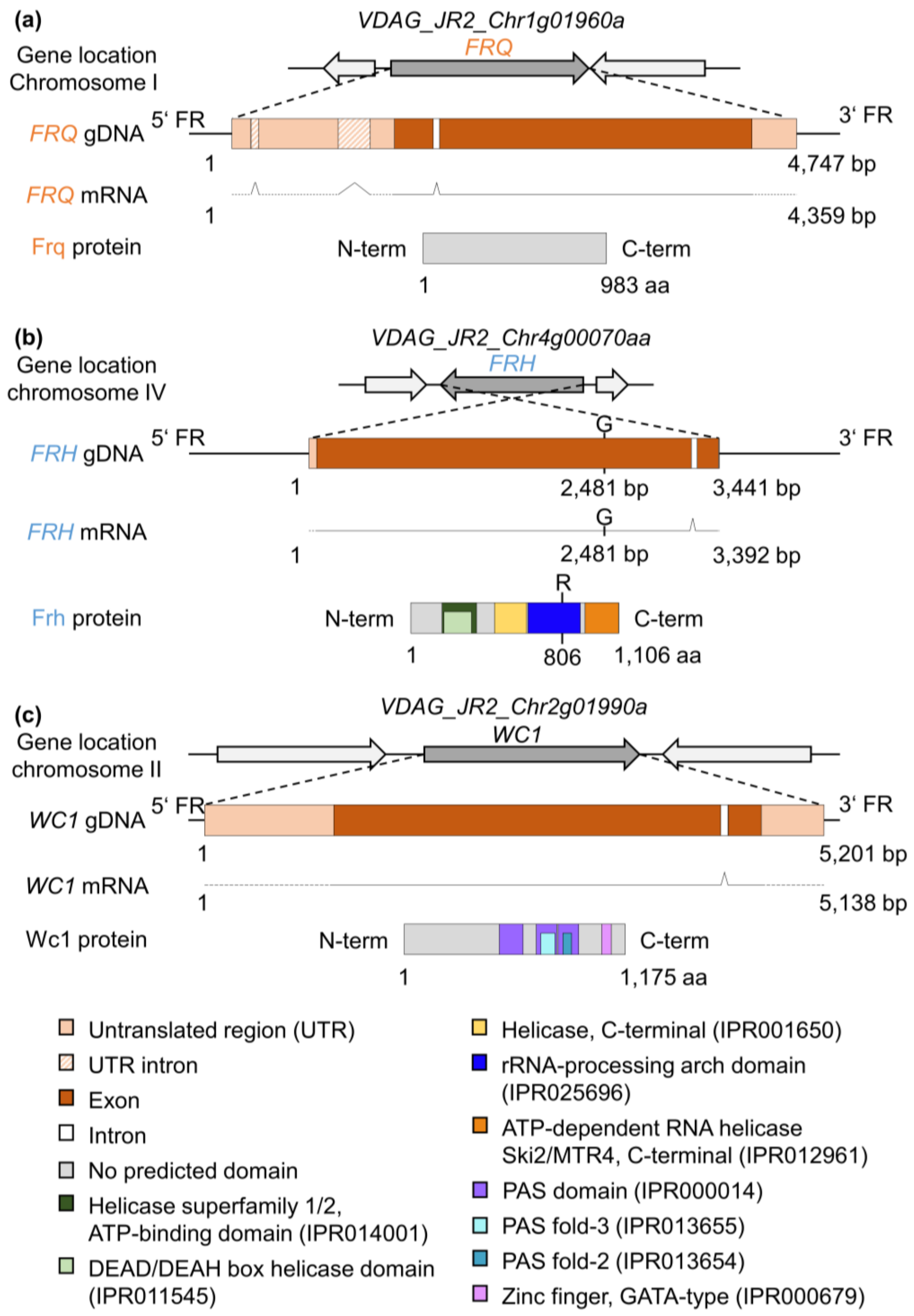

The figure legend is on the next page.

Figure S8. *V. dahliae FRQ*, *FRH*, and *WC1* genes with deduced protein structures. The genomic loci and gene annotations were retrieved from Ensembl Fungi [76] and protein domains predicted using InterPro [79]. Transcriptional directions of *V. dahliae* JR2 *FRQ*, *FRH*, *WC1*, and respective surrounding genes are indicated by arrows. **(a)** The Frq encoding gene (*VDAG_JR2_Chr1g01960a*) consists of two exons and one intron. The deduced protein is composed of 983 amino acids (aa). No domains were predicted, but aa 15 to 981 were assigned to belong to the frequency clock protein family (IPR018554). **(b)** The Frh encoding gene (*VDAG_JR2_Chr4g00070aa*) consists of two exons and one intron and encodes for the 1,106 aa Frh protein. For *FRH* point mutation, the guanine (G) at position 2,481 of the gene locus was changed into an adenine. This transition mutation causes a conservative amino acid exchange by changing the codon ‘CGC‘, translating to an arginine (R), to ‘CAC‘, which translates to a histidine (H). **(c)** *WC1* (*VDAG_JR2_Chr2g01990a*) consists of two exons and one intron. The deduced protein is 1,175 aa in length.

Figure S9

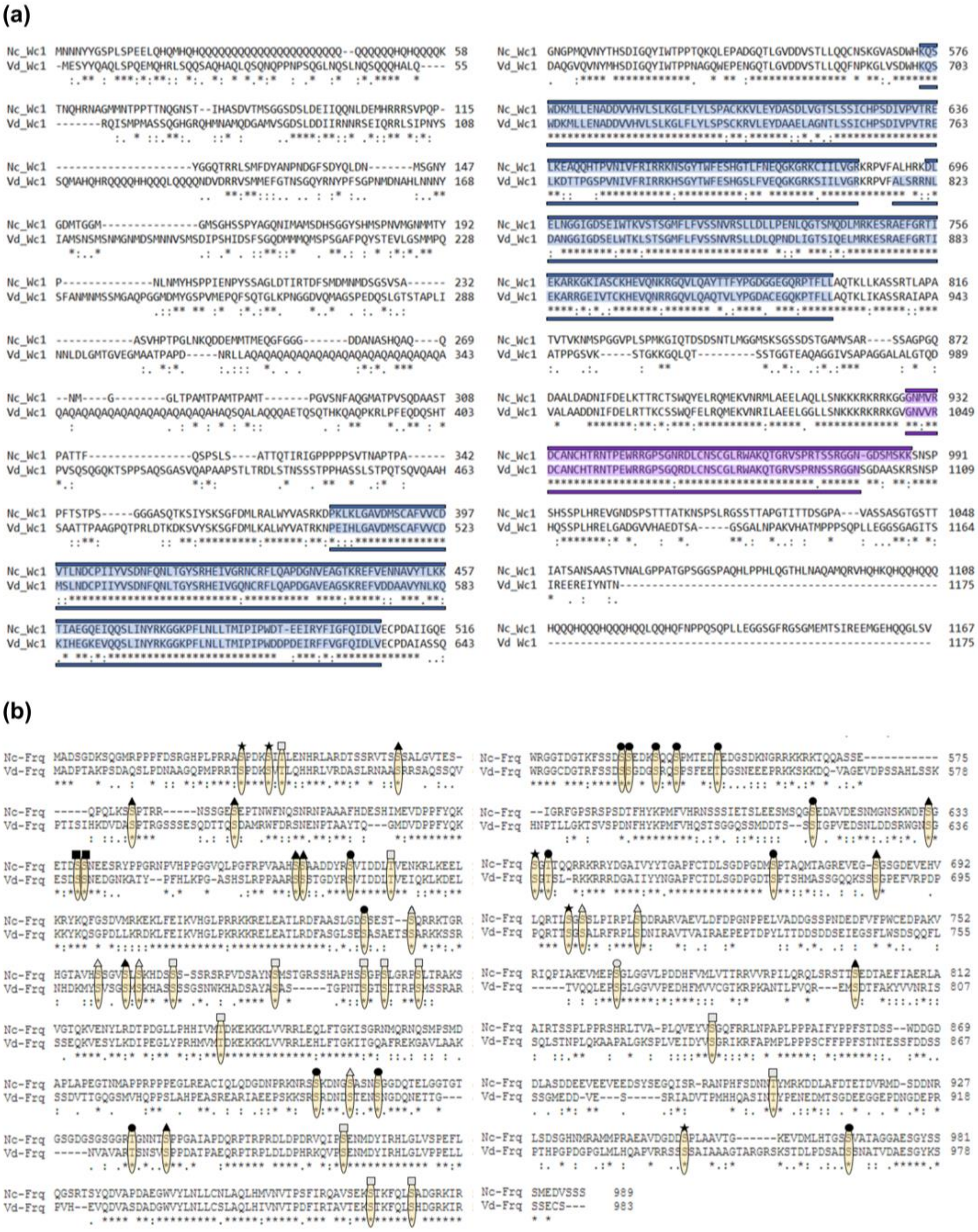

Figure S9. Alignments of Wc1 and Frq protein sequences from *N. crassa* and *V. dahliae* JR2. The protein sequences of *N. crassa* and *V. dahliae* JR2 were retrieved from FungiDB [77] and Ensembl Fungi [76], respectively. Sequence alignments were performed using Clustal Omega [81]. **(a)** Alignment of *N. crassa* Wc-1 (NCU02356, Nc_Wc1) and *V. dahliae* Wc1 (VDAG_JR2_Chr2g01990a, Vd_Wc1). Blue: PAS domain (IPR000014); purple: Zinc finger, GATA-type (IPR000679). **(b)** Alignment of *N. crassa* Frq (NCU02265, Nc-Frq) and *V. dahliae* Frq (VDAG_JR2_Chr1g01960a, Vd-Frq). Phosphorylation sites that were specifically and with high confidence identified in *N. crassa* [25,26,28] and are conserved in *V. dahliae* Frq are labeled. Symbols indicate how and in which study they were identified: Black star: identified in vivo in all three studies [25,26,28], black triangle: identified in in vivo studies [25,26], grey triangle: identified in vitro [26] and in vivo [25], black square: only identified in vivo in [26], grey square: only identified in vitro in [26], black circle: only identified in vivo in [25], grey circle: only identified in vivo in [28].

Figure S10

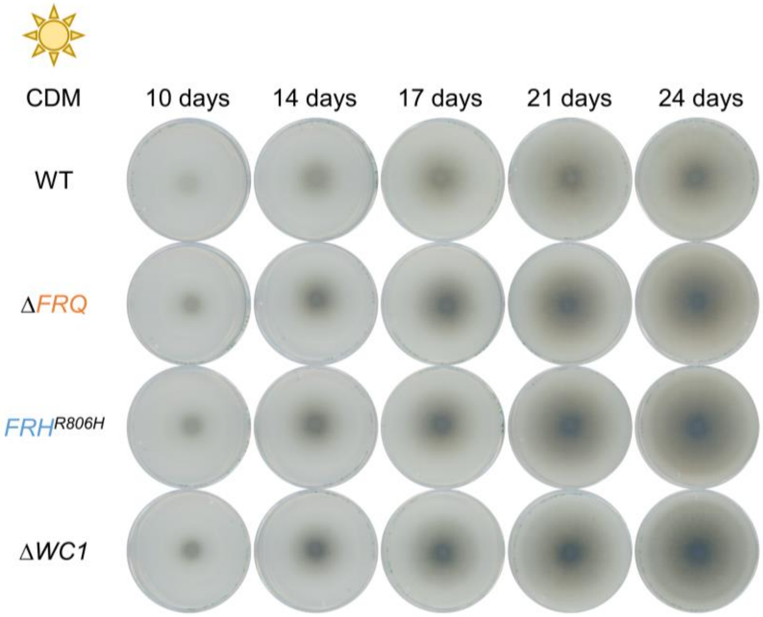

Figure S10. *V. dahliae FRQ, FRH*, and *WC1* mutant strains continuously melanize faster than wild-type. 50,000 spores of the *V. dahliae* wild-type (WT), *FRQ* deletion (Δ*FRQ*), *FRH* point mutation (*FRH*^{R806H}), and *WC1* deletion (Δ*WC1*) strains were point-inoculated onto CDM plates and incubated at 25 °C in the light for 24 days. Melanization was documented after ten, 14, 17, 21, and 24 days of incubation. The bottom of the plates is depicted. The colonies of the mutant strains were more melanized than the WT at the respective time points.

Figure S11

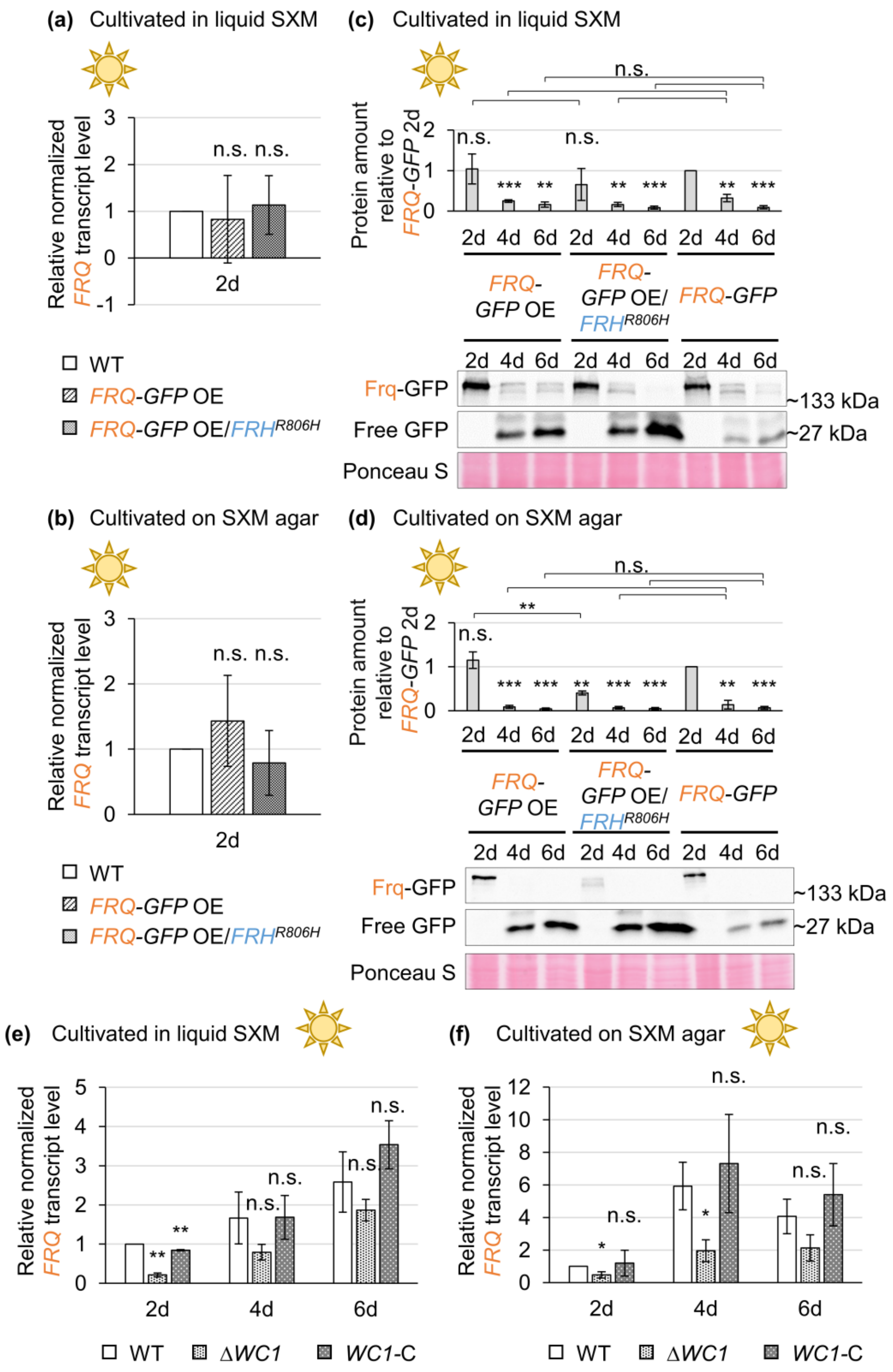

The figure legend is on the next page.

Figure S11. *FRQ–GFP* overexpression is prevented independent of Frh amino acid residue arginine 806 and the transcript level of *FRQ* depends partially, but not solely on Wc1. Transcript and protein levels of Frq were analyzed for the indicated strains. Cultures were inoculated with 1 × 10^6 spores into liquid SXM and with 4 × 10^6 spores on SXM agar covered with a nylon membrane. Flasks and plates were incubated at 25 °C in light for two (2d), four (4d), and six days (6d). *FRQ* transcript levels were investigated using quantitative reverse transcription PCR (qRT-PCR). The reference genes *H2A* and *EIF2B* were used for normalization. Protein levels of the Frq–GFP fusion protein were analyzed in western experiments using a GFP antibody. Ponceau S staining served as a control and for normalization. Depicted are the means of three biological replicates with respective standard deviation. **(a-d)** Transcript and protein levels were analyzed. The *V. dahliae* wild-type (WT) and mutant strains expressing Frq–GFP either under native promoter control (*FRQ–GFP*) or *gpdA* promoter control with wild-type *FRH* (*FRQ–GFP* OE) or point mutated *FRH* (*FRQ–GFP* OE/FRH^{R806H}) were included. **(a, b)** *FRQ* transcript levels were analyzed after 2d cultivation **(a)** in liquid SXM or **(b)** on SXM agar. WT levels were set to one. The *FRQ* transcript level was not significantly affected by *gpdA* promoter driven expression independent of the *FRH* point mutation (calculated with *t*-tests, n.s.: not significant). **(c, d)** Frq–GFP fusion protein amounts were quantified for extracts of mycelia grown **(c)** in liquid SXM or **(d)** on SXM agar for 2d, 4d, and 6d. Images of representative replicates are depicted below the graphs. The 2d Frq–GFP level of the *FRQ–GFP* strain was set as one. Statistical significance of differences compared with the fusion protein level of the *FRQ–GFP* strain after 2d was determined with *t*-tests and is indicated on top of the bars (n.s.: not significant, **: $p < 0.01$, ***: $p < 0.001$). As labeled above connecting lines, differences between respective 4d or 6d time points were not significant. Frq–GFP levels were not increased in the *FRQ–GFP* OE strain. The amino acid exchange in Frh did not result in increased fusion protein levels, but after 2d cultivation on SXM agar even led to significantly reduced Frq–GFP amounts. **(e-f)** Transcript levels of *FRQ* were analyzed for the WT, *WC1* deletion (Δ*WC1*), and complementation (*WC1*-C) strain, which were cultivated **(e)** in liquid SXM or **(f)** on SXM agar for 2d, 4d, and 6d. WT transcript levels after two days of incubation were set as one. Statistical analysis was conducted using *t*-tests (n.s.: not significant, *: $p < 0.05$, **: $p < 0.01$). Significances of differences compared with wild-type *FRQ* transcript levels at the same time point are depicted on top of the bars. *FRQ* transcript levels were significantly reduced upon deletion of *WC1* at early time points.

Figure S12

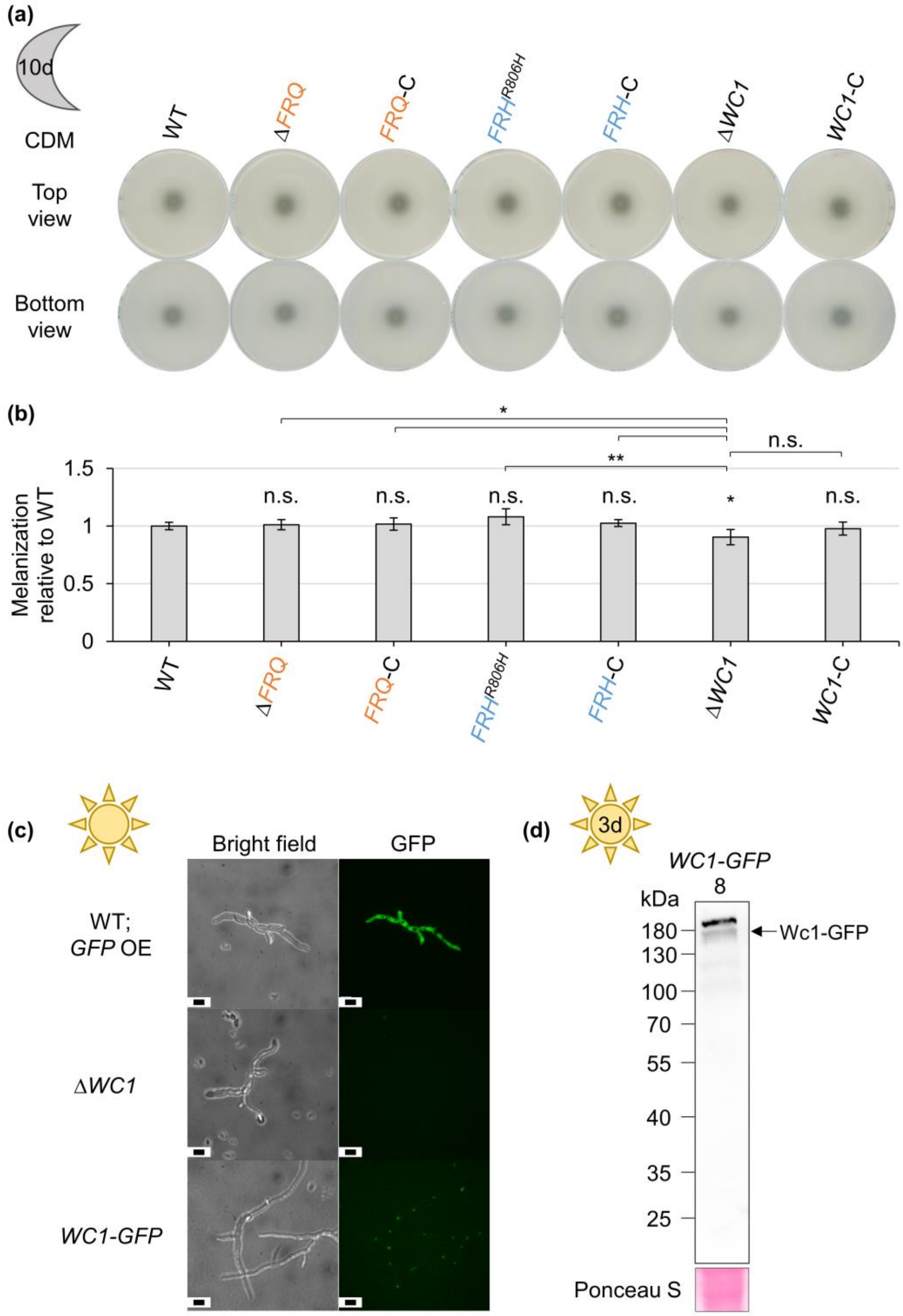

The figure legend is on the next page.

Figure S12. Wc1 slightly enhances microsclerotia formation in the dark and the *WC1–GFP* strain produces the Wc1–GFP fusion protein. **(a, b)** 50,000 spores of the *V. dahliae* wild-type (WT), *FRQ* deletion (Δ*FRQ*), *FRH* point mutation (FRH^{R806H}), *WC1* deletion (Δ*WC1*), and respective complementation strains (*FRQ*-C, *FRH*-C, *WC1*-C) were point-inoculated onto CDM agar and incubated in the dark for ten days. **(a)** Representative plates are depicted. **(b)** Melanization of the colony center was quantified in two independent experiments with two biological replicates each (N = 4). Statistical significances were calculated with *t*-tests (n.s.: not significant, *: $p < 0.05$, **: $p < 0.01$). Wc1 positively affected melanization in the dark, whereas Frq and Frh^{R806} were dispensable. This indicates that Wc1 enhances microsclerotia formation in the dark. **(c, d)** Production of the Wc1–GFP fusion protein by a *WC1–GFP*-expressing strain (*WC1–GFP*) was verified through fluorescence microscopy and a western experiment. **(c)** For fluorescence microscopy, spores of the *WC1–GFP* strain were inoculated into PDM and incubated overnight at 25 °C in the light. The *V. dahliae* WT ectopically overexpressing *GFP* (WT/*GFP* OE) and the *WC1* deletion strain (Δ*WC1*) served as controls. Wc1–GFP presumably localized in the nuclei. Scale bar: 10 µm. **(d)** The protein extract of *WC1–GFP* mutant transformant eight (VGB667) was obtained after cultivation in PDM in the light for three days. Depicted is the result of a western experiment using a GFP antibody. The Wc1–GFP fusion protein was detected at approximately 155 kDa as predicted. An additional signal was detected at slightly above 180 kDa. The signal above 180 kDa corresponds most likely to post-translationally modified Wc1–GFP.

Figure S13

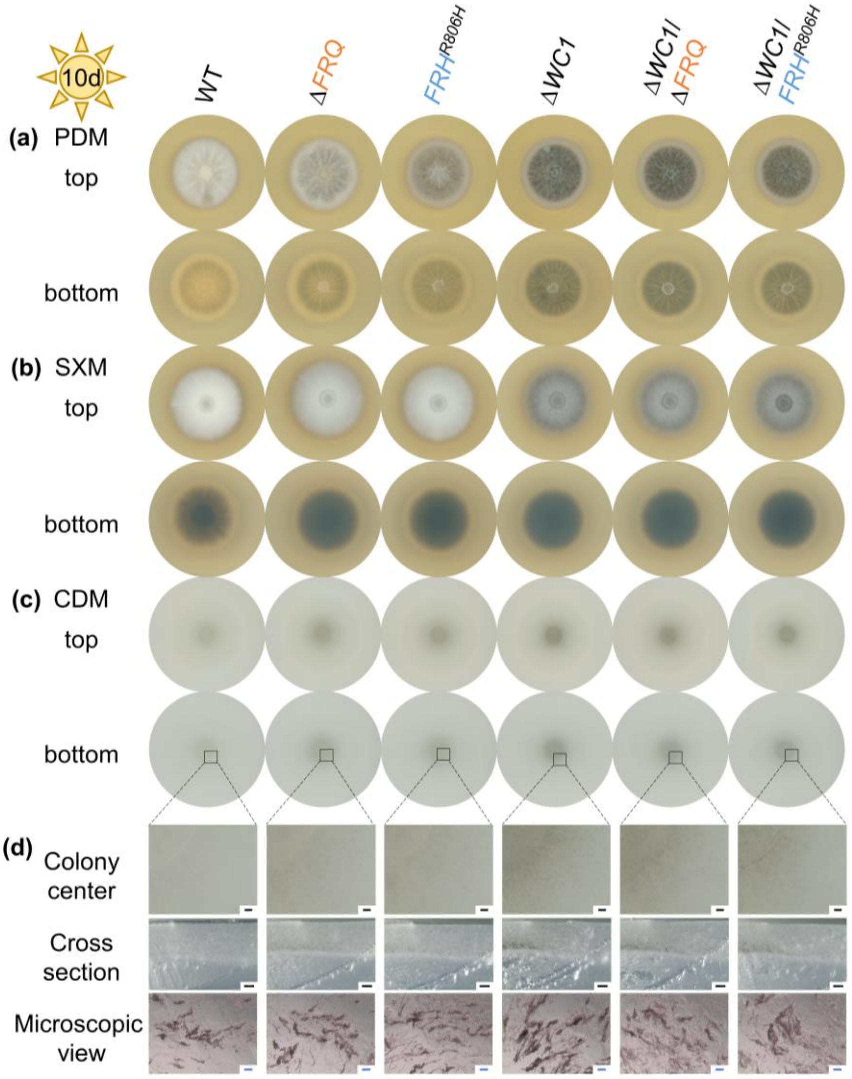

Figure S13. Ex planta phenotypes of the *WC1*/*FRQ* double-deletion or *WC1*/*FRH*^{R806H} double mutant strains equal the *WC1* single-deletion strain phenotype. 50,000 spores of the *V. dahliae* WT, *FRQ* deletion strain (Δ*FRQ*), *FRH* point mutation strain (*FRH*^{R806H}), *WC1* deletion strain (Δ*WC1*), and respective double mutant strains (Δ*WC1*/Δ*FRQ* and Δ*WC1*/ *FRH*^{R806H}) were point-inoculated onto **(a)** PDM, **(b)** SXM, and **(c)** CDM. The phenotype was investigated after ten days of incubation in the light. **(d)** Close-up images of the colony center, colony cross-section, and microsclerotia of colonies grown on CDM are depicted. *WC1* single-deletion and double mutant strains had the same ex planta phenotype. Deletion of *WC1*, like *FRQ* deletion and *FRH* point mutation, led to stronger melanization than observed in the WT. *WC1* deletion mutant strains produced fewer aerial hyphae than the other strains on PDM and SXM. Black scale: 500 µm, blue scale: 50 µm.

Figure S14

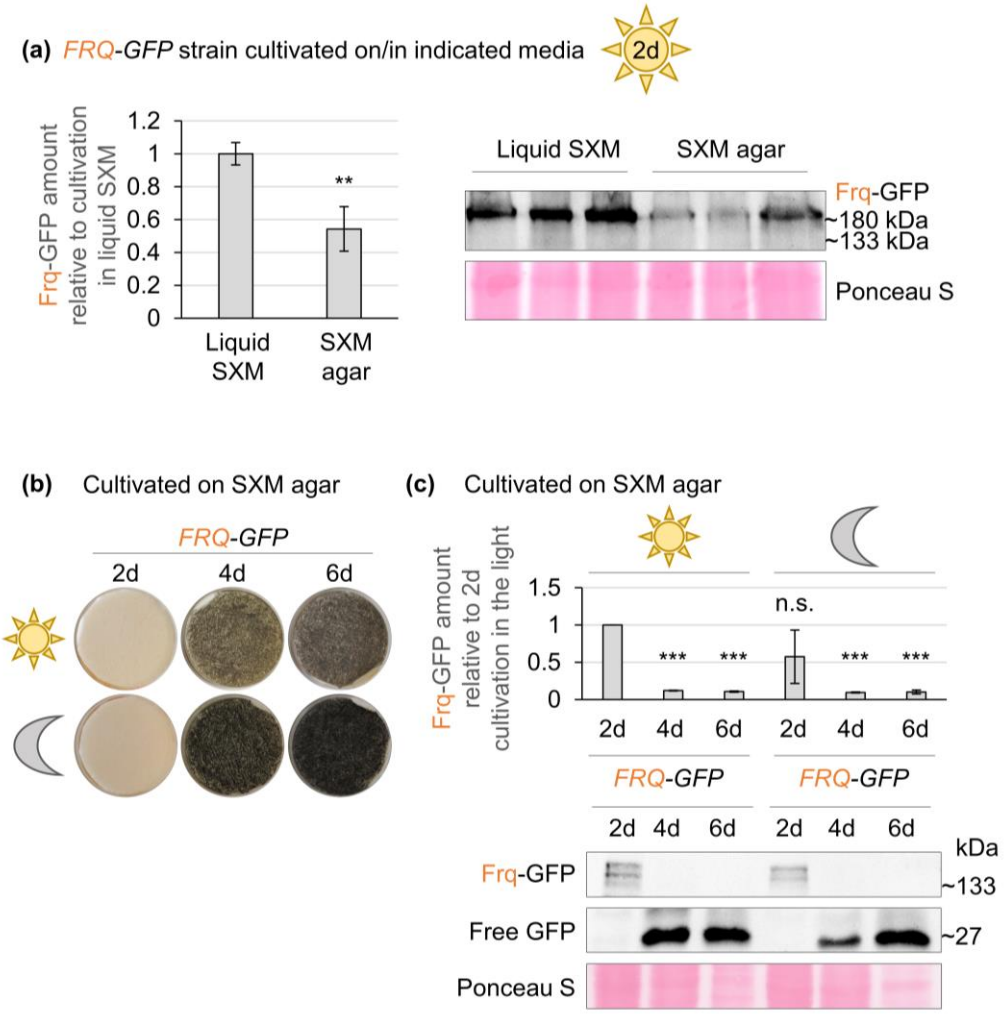

Figure S14. The Frq–GFP protein level depends on the cultivation conditions, which favor different developmental processes. The influence of different media and light conditions on Frq–GFP protein levels was investigated. Protein extracts of the *V. dahliae* strain expressing Frq–GFP under control of the native promoter (*FRQ–GFP)* were subjected to western experiments using a GFP antibody. Ponceau S staining was used for normalization. **(a)** 1 × 10^6 spores of the *FRQ–GFP* strain were inoculated into liquid SXM or onto SXM agar covered with a nylon membrane and grown at 25 °C in the light with agitation for two days. The Frq–GFP amount from cultivation in liquid SXM was set as one. Depicted is the mean of three biological replicates with respective standard deviation (left). Representative images of the western experiments are depicted (right). Statistical significance was determined using a *t*-test (**: $p < 0.01$). When wild-type was cultivated on SXM agar, favoring microsclerotia formation, Frq–GFP protein levels were significantly reduced compared with cultivation in liquid medium, which favors conidiation. **(b, c)** 4 × 10^6 spores of the *FRQ–GFP* strain were spread on SXM agar covered with a nylon membrane and grown at 25 °C in the light (sun) or darkness (moon) for two (2d), four (4d), and six (6d) days. **(b)** Representative cultures are depicted from top. Cultures that were incubated in darkness were more melanized. **(c)** In western experiments, comparable levels of Frq–GFP were detected for cultures incubated in the light or in the dark. One representative replicate is depicted below the graph. Statistical significance was determined with *t*-tests (n.s.: not significant, ***: $p < 0.001$).

Figure S15

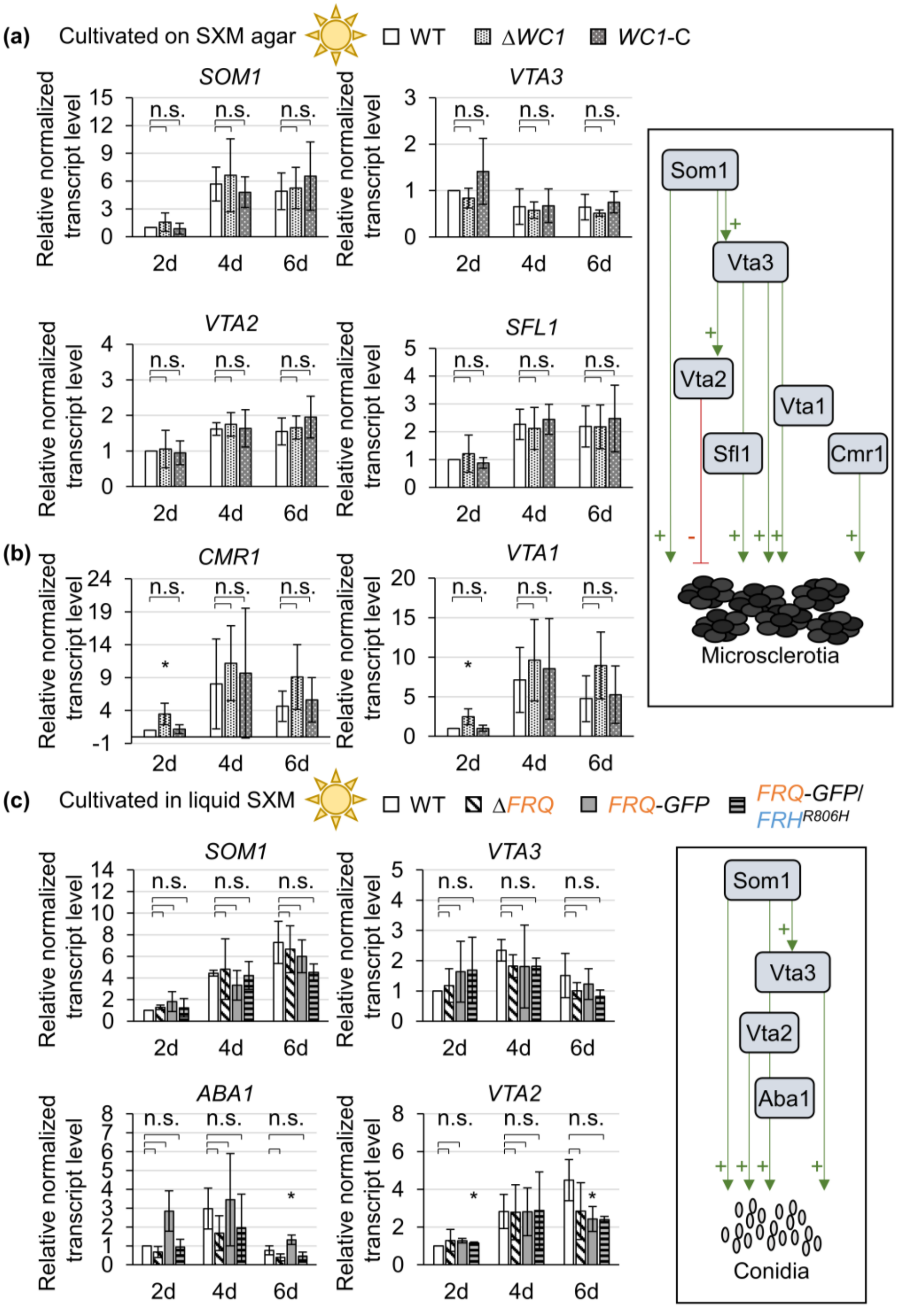

The figure legend is on the next page.

Figure S15. Wc1-, Frq-, and Frh-mediated control of *V. dahliae* development seems independent of the Som1- and Vta3-regulatory networks. Fresh spores of the *V. dahliae* wild-type (WT), *WC1* deletion and complementation strains (Δ*WC1*, *WC1*-C), *FRQ* deletion strain (Δ*FRQ*), and strains expressing an *FRQ–GFP* fusion construct either with wild-type or point mutated *FRH* (*FRQ–GFP*, *FRQ–GFP*/*FRH*^{R806H}) were **(a, b)** spread on SXM agar covered with a nylon membrane or **(c)** inoculated into liquid SXM. RNAs were extracted after cultivation at 25 °C for two (2d), four (4d), and six days (6d). Resulting cDNA was subjected to quantitative reverse transcription PCR. The schemes to the right display the regulatory effects of the tested transcription factors toward each other and microsclerotia or conidia development (green arrows/+: positive regulation, red lines/-: negative regulation). **(a, b)** Transcript levels of transcription factor encoding genes *SOM1*, *VTA3*, *VTA2*, *SFL1*, *CMR1*, and *VTA1,* involved in regulation of microsclerotia formation and/or melanin biosynthesis [61,67,68,71], were analyzed in WT, Δ*WC1,* and *WC1*-C at indicated time points. Bars represent the mean relative normalized transcript level of three biological replicates (N = 3) with standard deviation. The mean transcript level of *CMR1* and *VTA1* after 2d was calculated from six biological replicates (N = 6). Statistical significances were calculated using t-tests (n.s.: not significant, *: $p < 0.05$). Significance of differences compared with WT levels of the same time point are indicated through connecting lines. In the presence of an intact *WC1* gene, *CMR1* and *VTA1* transcript levels were reduced after 2d of cultivation on SXM agar, but not affected after four and six days. **(c)** Transcript levels of transcription factor encoding genes *SOM1*, *VTA3*, *ABA1*, and *VTA2,* involved in positive regulation of conidiation [61,67], were analyzed in WT, Δ*FRQ, FRQ–GFP*, and *FRQ–GFP*/*FRH*^{R806H} strains*.* Depicted are the means of three independent biological replicates (N = 3) with respective standard deviation. Statistical significances of differences to corresponding WT transcript levels were calculated with t-tests (n.s.: not significant, *: $p < 0.05$). None of the investigated transcription factors seem to be Frq- and Frh-dependently controlled on the transcript level.

Figure S16

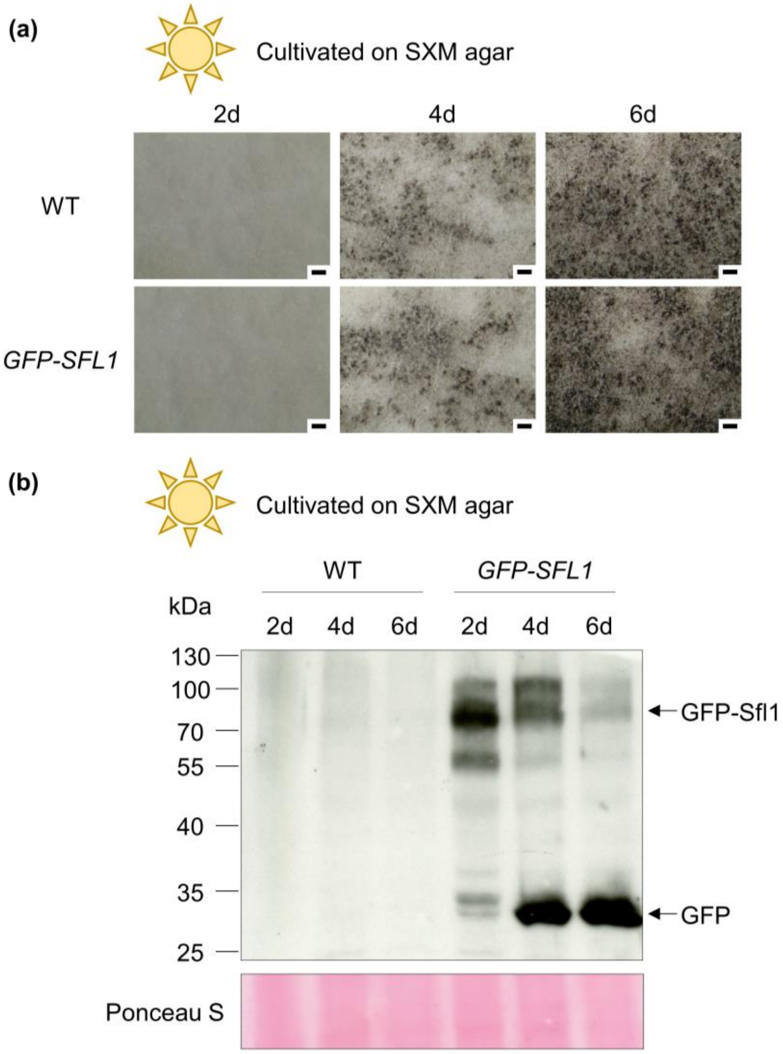

Figure S16. Sfl1 is required in the initial phase of microsclerotia production. **(a)** 1 × 10^6 spores of the *V. dahliae* wild-type (WT) and *SFL1* deletion strain endogenously expressing *GFP–SFL1* (*GFP–SFL1*) were spread on SXM agar covered with a nylon membrane and incubated at 25 °C in the light for two (2d), four (4d), or six (6d) days. Pictures of the cultures are depicted. Scale bar: 200 µm. **(b)** Protein extracts of cultures were subjected to a western experiment with a GFP antibody. WT served as negative control and Ponceau S staining was conducted as loading control. The GFP–Sfl1 fusion protein was detected at the predicted size of ~93 kDa and above. The strongest signal for GFP–Sfl1 was detected after two days and then it decreased over time. Free GFP as degradation product was increasingly detected after four and six days.

Figure S17

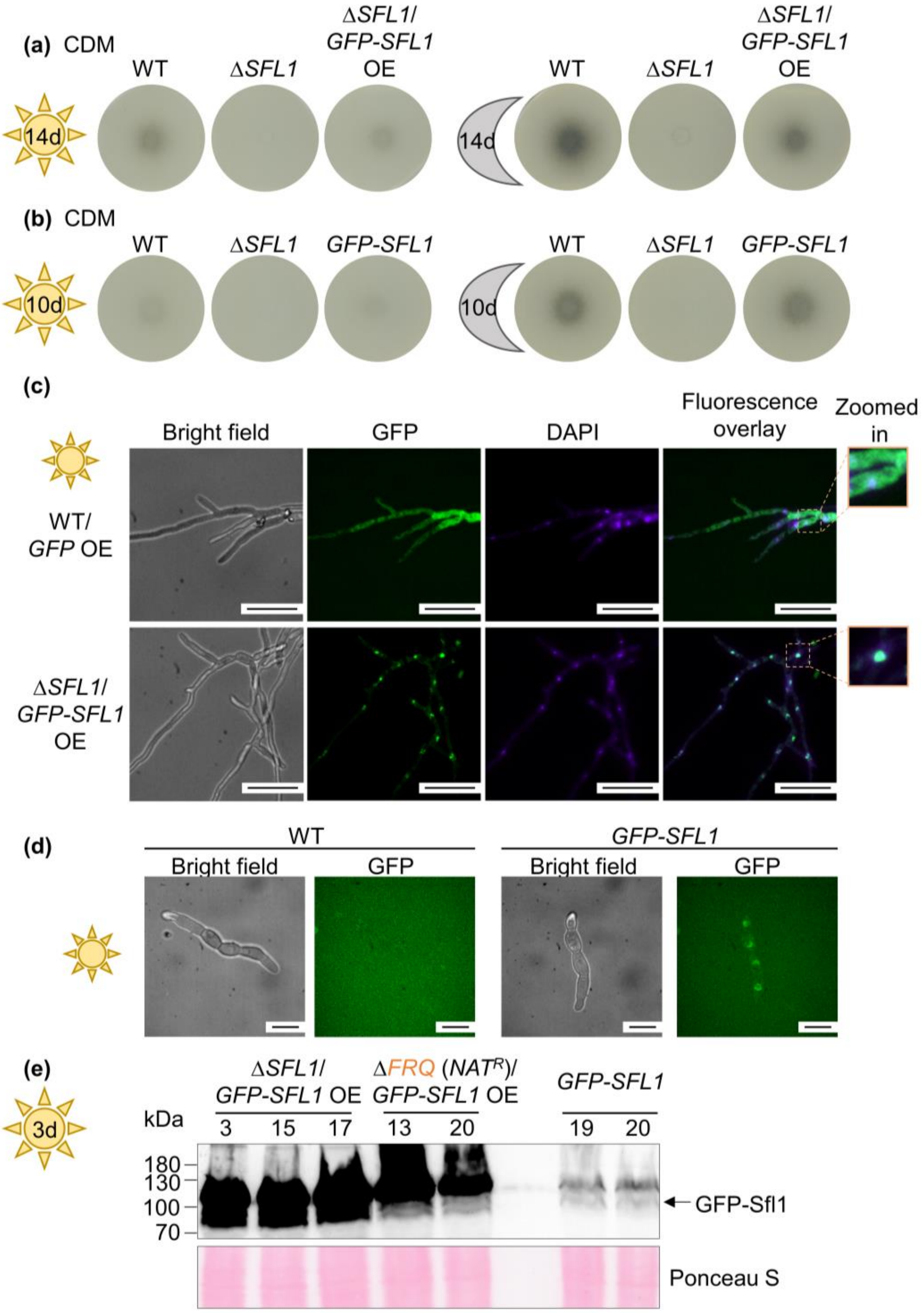

The figure legend is on the next page.

Figure S17. The GFP–Sfl1 fusion protein is present, functional, and predominantly localized in nuclei. *SFL1* deletion strains ectopically overexpressing *GFP–SFL1* (Δ*SFL1*/*GFP–SFL1* OE) or endogenously expressing *GFP–SFL1* in locus (*GFP–SFL1*) were tested for colony melanization **(a, b)** and fusion protein localization **(c, d)**. Fusion protein levels were compared in a western experiment **(e)** also including a *FRQ* deletion strain ectopically overexpressing *GFP–SFL1* (Δ*FRQ*/*GFP–SFL1* OE). **(a, b)** 50,000 spores of the *V. dahliae* wild-type (WT), *SFL1* deletion strain (Δ*SFL1*), Δ*SFL1*/*GFP–SFL1* OE, and *GFP–SFL1* strain were point-inoculated onto CDM and incubated at 25 °C in the light (left panel, sun) or in the dark (right panel, moon) for **(a)** 14 or **(b)** ten days. Depicted are top view pictures of the colonies. The *SFL1* deletion strain is impaired in melanization. Ectopic integration of the *GFP–SFL1* overexpression construct allowed melanization, which seemed slightly reduced compared with WT. The strain expressing endogenous levels of *GFP–SFL1* grew similar to the WT. **(c, d)** Subcellular localization of GFP–Sfl1 was analyzed via fluorescence microscopy. For this, spores were incubated at 25 °C in PDM overnight. GFP–Sfl1 predominantly localized in nuclei. **(c)** Nuclei were stained with DAPI (purple). A wild-type strain ectopically overexpressing *GFP* (WT/*GFP* OE) served as control. Scale bar = 20 µm. **(d)** Wild-type served as negative control. Scale bar = 10 µm. **(e)** Protein extracts of cultures grown at 25 °C in PDM for three days were subjected to a western experiment with GFP antibody. The GFP–Sfl1 fusion protein was detected to migrate slightly higher than the predicted size of ~93 kDa. Ponceau S staining served as loading control. All tested transformants of the *GFP–SFL1* overexpression strains produced more GFP–Sfl1 than detected in the strain endogenously expressing *GFP–SFL1*.

Figure S18

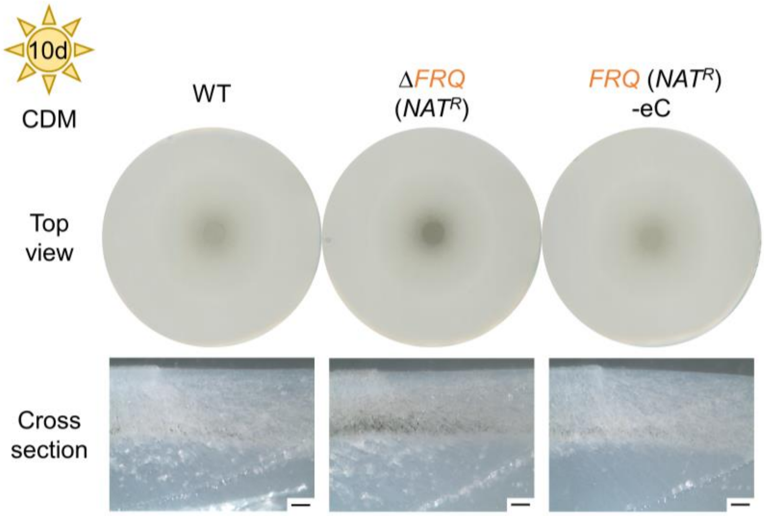

Figure S18. Ectopic *FRQ* complementation in the Δ*FRQ* (*NAT*^{R}) background allows for wild-type-like growth. 50,000 spores of wild-type (WT), the *FRQ* deletion strain with nourseothricin resistance cassette (Δ*FRQ* (*NAT*^{R})), and the ectopic complementation strain (*FRQ* (*NAT*^{R})-eC) were point-inoculated onto CDM and incubated at 25 °C in the light for ten days. Pictures of the colony top views and cross sections of the colony center are depicted. The *FRQ* deletion strain melanized more than wild-type, whereas the ectopic complementation strain melanized WT-like. Scale bar: 500 µm.

Supplementary tables

Table S1. Primers used in this study.

Primer name	Sequence (5' to 3')	Length (bp)	Overhang to (restriction site)
AN5	AGG TAA TCC TTC TTT GTC GAT GAC AAA TTC TTA TCA	36	*trpC* terminator
AN6	AGG ACT TCT AGA AGG GTG GGG TGT GCT ATG G	31	pME4548 (*Stu*I)
AN15	AGT AGA TGC CGA CCG G	16	-
AN16	ATT CTT AAT TAA GAT agg cct CCG CGA CGT TAA CTG ATA TT	41	pME4548 (*Stu*I) plus *Stu*I restriction site (agg cct)
AN17	CGG TCG GCA TCT ACT CTA TTC CTT TGC CCT CGG	33	*trpC* terminator
AN23	TTA ACG TCG CGG agg TTA CTT GTA CAG CTC GTC C	34	Half of *Stu*I restriction site (agg) and *trpC* promoter
AN30	CCT CCG CGA CGT TAA C	16	-
AN43	AGG TAA TCC TTC TTT GAT GAA GCG CAA ATG C	31	*trpC* terminator
AN44	AGG ACT TCT AGA AGG TTC TGT TTG ATC GAG CTG	33	pME4548 (*Stu*I)
AN45	GTC GAG GGT GGC CAT ATC GAT GCT TGG GTA GAA T	34	*NAT^R*
AN46	ATG GCC ACC CTC GAC G	16	-
AN47	AAA GAA GGA TTA CCT CTA AAC AAG TGT A	28	-
AN55	TGA GCA GAC ATC ACC ATG GCC GAC CCA ACG G	31	*gpdA* promoter
AN56	AGC GAG TGC AGT TAA CCG CGA CGT TAA CTG A	31	*FRQ*
AN57	CGT TGG GTC GGC CAT GGA TAT GGT GCG TCC C	31	*FRQ*
AN58	ACC ACC GCT ACC ACC ACT GCA CTC GCT GCT A	31	*GFP* linker
AN59	ATT CTT AAT TAA GAT AGG CCT GGT AAG TTT TCC TCT CGA CA	41	pME4548 (*Eco*RV)
AN60	AGG ACT TCT AGA AGG AAT ACT TGT CGC TGT CTC CG	35	pME4548 (*Stu*I)
AN61	CTT AAT TAA GAT AGG AGG TTA CAT AAG CCC ACG TG	35	pME5495
AN62	GGA TCT GAC TGA TGC TCT TC	20	-
AN63	GCA TCA GTC AGA TCC aCG TTT TCA TGC CTA AGG	33	*FRH*; introduces adenine (a) instead of guanine (G)

**Table S1.** Primers used in this study, continued.			
**Primer name**	**Sequence (5' to 3')**	**Length (bp)**	**Overhang to (restriction site)**
AN64	TTA CAG GTA CAA GCT CTG	18	-
AN65	AGC TTG TAC CTG TAA CCG CGA CGT TAA CTG A	31	*FRH*
AN66	GGA AAA CTT ACC AGG AAA GAA GGA TTA CCT CTA AAC	36	pME5495
AN67	ATG GAC GAC CTC TTC G	16	-
AN68	CCT ATC TTA ATT AAG AAT TCG TAA TCA TG	29	-
AN69	CCT GGT AAG TTT TCC TCT CG	20	-
AN70	TTT TTA CAG CCT GGC CG	17	-
AN71	ATC TGT GAT ATA CTG TGT GCT	21	-
AN73	ACC AGC CCC TGG GTT CCT GGT AAG TTT TCC TCT CG	35	*trpC* terminator
AN74	ATA TCA GTT AAC GTC TTA CAG GTA CAA GCT CTG CG	35	*trpC* promoter
AN76	ATG GAG TCC TAC TAT CAG GC	20	-
AN77	CTA GTT GGT GTT GTA AAT CTC TCG	24	-
AN80	ATT CTT AAT TAA GAT GAC GAC ATG ATG TCC TGT TTC	36	pME4548 (*Eco*RV)
AN81	ATA TCA GTT AAC GTC ATT GAG GGC ATC AGT ACT GG	35	*trpC* promoter
AN82	ACC AGC CCC TGG GTT ACG GCC ATC GAC ATC C	31	*trpC* terminator
AN83	AGG ACT TCT AGA AGG GAG AAA TCG ATC CAG CAG AG	35	pME4548 (*Stu*I)
AN91	AGG TAA TCC TTC TTT ACG GCC ATC GAC ATC C	31	*trpC* terminator
AN93	TAC AAC ACC AAC TAG CCG CGA CGT TAA CTG A	31	*WC1* gDNA
AN111	ACC ACC GCT ACC ACC GTT GGT GTT GTA AAT CTC TCG	36	*GFP* linker
AN114	AGG TAA TCC TTC TTT GGT AAG TTT TCC TCT CGA CAA	36	*trpC* terminator
AN115	ACC ACC GCT ACC ACC CAG GTA CAA GCT CTG CG	32	*GFP* linker
AN116	ATT CTT AAT TAA GAT TCG GGG TCA AGG TGC G	31	pME4548 (*Eco*RV)
AN117	CAG TTA ACG TCG CGG GGA AAA AGG CAG AGC CGA C	34	*trpC* promoter
AN118	CCG CGA CGT TAA CTG ATA TTG	21	-
AO165	GGT GGT AGC GGT GGT GT	17	-
JST253	GAC GTT AAC TGA TAT TGA AGG AGC AC	26	-
JST254	AAC CCA GGG GCT GGT GA	17	-
ML8	AAA GAA GGA TTA CCT CTA AAC AA	23	-
ML9	TGT ACA GTG ACC GGT GAC	18	-
ML31	GGT GAT GTC TGC TCA AGC GG	20	-

**Table S1.** Primers used in this study, continued.			
**Primer name**	**Sequence (5' to 3')**	**Length (bp)**	**Overhang to (restriction site)**
ML60	ATG GCC GAC CCA ACG G	16	-
ML61	TTA ACT GCA CTC GCT GCT ACT CTT	24	-
ML67	GGT ACC GAG CTC GAT TTT CTG AGG AGA TGC GAG AAG	36	pPK2 (*Eco*RV)
ML73	GGT GGT AGC GGT GGT ATG GCT GCC GCG ATT GAG A	34	*GFP* linker
ML74	AAA CGG ATG CAG TGA TCC ACT TAA CGT TAC TGA AAT CAT	39	*SFL1*
ML77	GCC CTT GCT CAC CAT TTG CGC TGC ACG ATG C	31	*GFP*
ML78	AGA TCC CCG GGT ACC TCA CTG CAT CCG TTT GCG	33	*gpdA* promoter
ML79	AGA TCC CCG GGT ACC GGA TAT GGT GCG TCC CCA	33	*gpdA* promoter
ML114	TGT TGT GTG GAA GAT AGG CCA CAC CAT CCC ACT	33	pME4815 (*Eco*RV)
ML115	GGT CAC TGT ACA GAT TTT CTG AGG AGA TGC GAG AAG	36	pME4815 (*Eco*RV)
RH631	ATT CTT AAT TAA GAT AGG CCA CAC CAT CCC ACT	33	pME4564 (*Eco*RV)
RH632	ACC GGT CAC TGT ACA GGA TAT GGT GCG TCC CCA	33	*gpdA* promoter
RH633	AGG TAA TCC TTC TTT TGC TGC CAC GGA CGT ATT	33	*trpC* terminator
RH634	AGG ACT TCT AGA AGG TTT CTG AGG AGA TGC GAG AAG	36	pME4564 (*Stu*I)
RH635	GGT ACC GAG CTC GAT GTG ACC GGT GAC TCT TTC TG	35	pPK2 (*Eco*RV)
RH636	ATT CTT AAT TAA GAT CGA GTG GAG ATG TGG AGT G	34	pPK2 (*Eco*RV)
RH659	ACC GGT CAC TGT ACA TTG CGC TGC ACG ATG C	31	*gpdA* promoter
RH660	AGG TAA TCC TTC TTT TAT CGT CTC TCC GCA ACC G	34	*trpC* terminator
RH664	ATG GCT GCC GCG ATT GA	17	-
RH665	TCA CTG CAT CCG TTT GCG	18	-
RH667	ATT CTT AAT TAA GAT AGC GTC CCC TTG CCC ATT	33	pME4548 (*Eco*RV)
RH668	GTC GCA CAA AAG AGG AGA TGT TG	23	-
RO3	GGT ACC CGG GGA TCT TTC G	19	-
SZ19	ACC TCT GGA GGC AAG GCT T	19	-
SZ20	GCT TGG CCT TCT TCT TCT GC	20	-
ZQY10	ATG GTG AGC AAG GGC GAG	18	-
ZQY11	ACC ACC GCT ACC ACC CTT GTA CAG CTC GTC CAT GC	35	*GFP* linker

Table S2. Plasmids used in this study.

Plasmid	Description	Reference
pCOM	$^{P}trpC{:}GEN^{R}{:}trpC^{T}$; KAN^{R}, left and right border for ATMT	[110]
pGreen2	$^{P}gpdA{:}GFP{:}trpC^{T}{:}^{P}gpdA{:}HYG^{R}{:}trpC^{T}$; KAN^{R},<br>left and right border for ATMT	[67]
pJet1.2	Cloning vector with AMP^{R}	Thermo Fisher Scientific
pME4548	$^{P}trpC{:}NAT^{R}$; KAN^{R}<br>Cloning vector with NAT^{R} without terminator and KAN^{R}, use *Eco*RV and *Stu*I digestion for exchange of $^{P}trpC{:}NAT^{R}$ with the desired fragment, left and right border for ATMT	[61]
pME4564	$^{P}trpC{:}HYG^{R}$; KAN^{R}<br>Cloning vector with HYG^{R} without terminator and KAN^{R}, use *Eco*RV and *Stu*I digestion for exchange of $^{P}trpC{:}HYG^{R}$ with the desired fragment, left and right border for ATMT	[60]
pME4727	$^{P}SFL1{:}^{P}gpdA{:}NAT^{R}{:}trpC^{T}{:}SFL1^{T}$ in pME4548	[61]
pME4815	$^{P}gpdA{:}NAT^{R}{:}trpC^{T}$ in pME4564	[60]
pME4876	$^{P}gpdA{:}GFP{:}SFL1{:}trpC^{T}{:}^{P}gpdA{:}HYG^{R}{:}trpC^{T}$ in pPK2	This study
pME4877	$^{P}SFL1{:}GFP{:}SFL1{:}^{P}gpdA{:}HYG^{R}{:}trpC^{T}{:}SFL1^{T}$ in pME4548	This study
pME4879	$^{P}FRQ{:}^{P}gpdA{:}NAT^{R}{:}trpC^{T}{:}FRQ^{T}$ in pME4564	This study
pME4880	$^{P}FRQ{:}FRQ{:}FRQ^{T}{:}^{P}gpdA{:}HYG^{R}{:}trpC^{T}$ in pPK2	This study
pME4881	$^{P}FRQ{:}^{P}gpdA{:}HYG^{R}{:}trpC^{T}{:}FRQ^{T}$ in pME4564	This study
pME4882	$^{P}FRQ{:}FRQ{:}FRQ^{T}{:}^{P}gpdA{:}NAT^{R}{:}trpC^{T}$ in pME4564	This study
pME4976	$^{P}gpdA{:}RFP{:}H2B{:}trpC^{T}$ in pCOM	[69]
pME5072	$^{P}VEL1{:}VEL1{:}GFP{:}^{P}gpdA{:}HYG^{R}{:}trpC^{T}{:}VEL1^{T}$ in pME4564	[70]
pME5489	$^{P}trpC{:}NAT^{R}{:}trpC^{T}{:}TYR^{T}$ in pME4548	This study
pME5490	$^{P}trpC{:}HYG^{R}{:}trpC^{T}{:}DOD^{T}$ in pME4548	This study
pME5491	$^{P}FRQ{:}^{P}gpdA{:}FRQ{:}^{P}trpC{:}NAT^{R}{:}trpC^{T}{:}FRQ^{T}$ in pME4564	This study
pME5492	$^{P}FRQ{:}^{P}gpdA{:}FRQ{:}GFP{:}^{P}trpC{:}NAT^{R}{:}trpC^{T}{:}FRQ^{T}$<br>in pME4564	This study
pME5493	$^{P}FRQ{:}FRQ{:}^{P}trpC{:}NAT^{R}{:}trpC^{T}{:}FRQ^{T}$ in pME4564	This study
pME5494	$^{P}FRQ{:}FRQ{:}GFP{:}^{P}trpC{:}NAT^{R}{:}trpC^{T}{:}FRQ^{T}$ in pME4564	This study
pME5495	FRH^{T} in pME4548	This study
pME5496	$^{P}FRH{:}FRH^{R806H}{:}^{P}trpC{:}HYG^{R}{:}trpC^{T}{:}FRH^{T}$ in pME4548	This study
pME5497	$^{P}FRH{:}FRH{:}^{P}trpC{:}GEN^{R}{:}trpC^{T}{:}FRH^{T}$ in pME4548	This study
pME5498	$^{P}FRH{:}FRH{:}GFP{:}^{P}trpC{:}NAT^{R}{:}trpC^{T}{:}FRH^{T}$ in pME4548	This study
pME5499	$^{P}FRH{:}FRH^{R806H}{:}GFP{:}^{P}trpC{:}NAT^{R}{:}trpC^{T}{:}FRH^{T}$ in pME4548	This study
pME5500	$^{P}FRH{:}^{P}trpC{:}HYG^{R}{:}trpC^{T}{:}FRH^{T}$ in pME4548	This study
pME5501	$^{P}WC1{:}^{P}trpC{:}GEN^{R}{:}trpC^{T}{:}WC1^{T}$ in pME4548	This study

**Table S2.** Plasmids used in this study, continued.		
**Plasmid**	**Description**	**Reference**
pME5502	^{P}*WC1*:*WC1*:^{P}*trpC*:*NAT*^{R}:*trpC*^{T}:*WC1*^{T} in pME4548	This study
pME5503	^{P}*WC1*:*WC1*:*GFP*:^{P}*trpC*:*NAT*^{R}:*trpC*^{T}:*WC1*^{T} in pME4548	This study
pPK2	^{P}*gpdA*:*HYG*^{R}:*trpC*^{T}; *KAN*^{R}<br>Cloning vector with *KAN*^{R} and *HYG*^{R}, use *Eco*RV for linearization and fragment integration, left and right border for ATMT	[109]

AMP^{R}: ampicillin resistance marker; ATMT: *Agrobacterium tumefaciens*-mediated transformation; *GEN*^{R}: geneticin resistance marker; *HYG*^{R}: hygromycin B resistance marker; *KAN*^{R}: kanamycin resistance marker; *NAT*^{R}: nourseothricin resistance marker; ^{P}: promoter; ^{T}: terminator

Table S3. List of bacterial and fungal strains used in this work.

Strain	Description	Reference
Agrobacterium tumefaciens		
A. tumefaciens AGL1	Chemically competent cells for *Agrobacterium tumefaciens*-mediated transformation of *V. dahliae*	[87]
Escherichia coli		
E. coli DH5α	Chemically competent cells for cloning and plasmid propagation	Invitrogen, Thermo Fisher Scientific
Verticillium dahliae		
JR2/ WT	*Solanum lycopersicum* isolate	[75]
VGB45<br>(WT/*GFP* OE)	JR2 overexpressing ectopically integrated *GFP*<br>(^{P}*gpdA:GFP:trpC*^{T}:^{P}*gpdA:HYG*^{R}*:trpC*^{T})	[67]
VGB266/<br>VGB348/<br>VGB349<br>(Δ*SFL1/GFP–SFL1* OE)	*SFL1* deletion strain ectopically overexpressing *GFP–SFL1*<br>(Δ*SFL1::*^{P}*gpdA:NAT*^{R}*:trpC*^{T};<br>^{P}*gpdA:GFP:SFL1:trpC*^{T}:^{P}*gpdA:HYG*^{R}*:trpC*^{T})	This study
VGB296<br>(Δ*FRQ* (*NAT*^{R}))	*FRQ* deletion strain with *NAT* resistance marker<br>(Δ*FRQ::*^{P}*gpdA:NAT*^{R}*:trpC*^{T})	This study
VGB324/<br>VGB325<br>(Δ*SFL1*)	*SFL1* deletion strain<br>(Δ*SFL1::*^{P}*gpdA:NAT*^{R}*:trpC*^{T})	[61]
VGB342/<br>VGB343<br>(*SFL1*-eC)	*SFL1* ectopic complementation strain<br>(Δ*SFL1::*^{P}*gpdA:NAT*^{R}*:trpC*^{T};<br>^{P}*SFL1:SFL1:SFL1*^{T}:^{P}*gpdA:HYG*^{R}*:trpC*^{T})	[61]
VGB353/<br>VGB354<br>(*FRQ–GFP*)	*FRQ–GFP*-expressing strain<br>(Δ*FRQ::*^{P}*FRQ:FRQ:GFP:*^{P}*trpC:NAT*^{R}*:trpC*^{T}*:FRQ*^{T})	This study
VGB402/<br>VGB403<br>(Δ*FRQ*)	*FRQ* deletion strain with *HYG* resistance marker<br>(Δ*FRQ::*^{P}*gpdA:HYG*^{R}*:trpC*^{T})	This study
VGB404/<br>VGB405<br>(Δ*FRQ*/Δ*SFL1*)	*FRQ* and *SFL1* double-deletion strain<br>(Δ*FRQ::*^{P}*gpdA:HYG*^{R}*:trpC*^{T};<br>Δ*SFL1::*^{P}*gpdA:NAT*^{R}*:trpC*^{T})	This study
VGB411<br>(*FRQ* (*NAT*^{R})-eC)	Ectopic *FRQ* complementation in *FRQ* deletion with *NAT* resistance marker<br>(Δ*FRQ::*^{P}*gpdA:NAT*^{R}*:trpC*^{T};<br>^{P}*FRQ:FRQ:FRQ*^{T}:^{P}*gpdA:HYG*^{R}*:trpC*^{T})	This study
VGB433/<br>VGB434<br>(*GFP–SFL1*)	*GFP–SFL1*-expressing strain<br>(Δ*SFL1::*^{P}*SFL1:GFP:SFL1:*^{P}*gpdA:HYG*^{R}*:trpC*^{T}*:SFL1*^{T})	This study

Table S3. List of bacterial and fungal strains used in this work, continued.

**Strain**	**Description**	**Reference**
VGB435/ VGB436 (Δ*FRQ* (*NAT*^{R})/ *GFP–SFL1* OE)	*FRQ* deletion strain ectopically overexpressing *GFP–SFL1* ($\Delta FRQ::{}^{P}gpdA:NAT^{R}:trpC^{T}; {}^{P}gpdA:GFP:SFL1:trpC^{T}:{}^{P}gpdA:HYG^{R}:trpC^{T}$)	This study
VGB441/ VGB442 (*FRQ*-eC)	Ectopic *FRQ* complementation in *FRQ* deletion with *HYG* resistance marker ($\Delta FRQ::{}^{P}gpdA:HYG^{R}:trpC^{T}; {}^{P}FRQ:FRQ:FRQ^{T}:{}^{P}gpdA:NAT^{R}:trpC^{T}$)	This study
VGB477 (WT/*RFP–H2B*)	JR2 overexpressing ectopically integrated *RFP–H2B* (${}^{P}gpdA:RFP:H2B:trpC^{T}:{}^{P}gpdA:GEN^{R}:trpC^{T}$)	[69]
VGB507/ VGB508 (*FRQ*-C)	*FRQ* complementation at the endogenous locus in *FRQ* deletion with *NAT* resistance marker($\Delta FRQ::{}^{P}FRQ:FRQ:{}^{P}trpC:NAT^{R}:trpC^{T}:FRQ^{T}$)	This study
VGB516/ VGB517 (*FRQ–GFP* OE)	Strain expressing *FRQ–GFP* under *gpd*A promoter control ($\Delta FRQ::{}^{P}gpdA:FRQ:GFP:{}^{P}trpC:NAT^{R}:trpC^{T}$)	This study
VGB539/ VGB540 (*FRQ–GFP*/ *RFP–H2B*)	*FRQ–GFP*-expressing strain ectopically overexpressing *RFP–H2B* ($\Delta FRQ::{}^{P}FRQ:FRQ:GFP:{}^{P}trpC:NAT^{R}:trpC^{T}:FRQ^{T}$; ${}^{P}gpdA:RFP:H2B:trpC^{T}:{}^{P}gpdA:GEN^{R}:trpC^{T}$)	This study
VGB541/ VGB542 (*FRH*^{R806H})	*FRH*^{R806H} point mutation strain ($FRH^{R806H}::{}^{P}FRH:FRH^{R806H}:{}^{P}trpC:HYG^{R}:trpC^{T}:FRH^{T}$)	This study
VGB543/ VGB544 (*FRQ–GFP*/ *FRH*^{R806H})	*FRH*^{R806H} point mutation strain with *FRQ–GFP* expression ($\Delta FRQ::{}^{P}FRQ:FRQ:GFP:{}^{P}trpC:NAT^{R}:trpC^{T}:FRQ^{T}$; $FRH^{R806H}::{}^{P}FRH:FRH^{R806H}:{}^{P}trpC:HYG^{R}:trpC^{T}:FRH^{T}$)	This study
VGB545/ VGB546 (*FRQ–GFP* OE/*FRH*^{R806H})	*FRH*^{R806H} point mutation strain with *gpdA* promoter-controlled *FRQ–GFP* expression ($\Delta FRQ::{}^{P}gpdA:FRQ:GFP:{}^{P}trpC:NAT^{R}:trpC^{T}$; $FRH^{R806H}::{}^{P}FRH:FRH^{R806H}:{}^{P}trpC:HYG^{R}:trpC^{T}:FRH^{T}$)	This study
VGB577/ VGB578 (*FRH*-C)	*FRH* complementation strain ($FRH^{R806H}::{}^{P}FRH:FRH:{}^{P}trpC: GEN^{R}:trpC^{T}:FRH^{T}$)	This study
VGB630/ VGB631 (Δ*WC1*)	*WC1* deletion strain ($\Delta WC1::{}^{P}trpC:GEN^{R}:trpC^{T}$)	This study
VGB664/ VGB665 (*WC1*-C)	*WC1* complementation strain ($\Delta WC1::{}^{P}WC1:WC1:{}^{P}trpC:NAT^{R}:trpC^{T}:WC1^{T}$)	This study
VGB667 (*WC1–GFP*)	*WC1–GFP*-expressing strain ($\Delta WC1::{}^{P}WC1:WC1:GFP:{}^{P}trpC:NAT^{R}:trpC^{T}:WC1^{T}$)	This study

Table S3. List of bacterial and fungal strains used in this work, continued.

Strain	Description	Reference
VGB695/ VGB696 (*FRH–GFP*)	*FRH–GFP*-expressing strain (*FRH*^{R806H}::^{P}*FRH:FRH:GFP*:^{P}*trpC:NAT*^{R}:*trpC*^{T}:*FRH*^{T})	This study
VGB697/ VGB698 (*FRH*^{R806H}–*GFP*)	*FRH*^{R806H}–*GFP*-expressing strain (*FRH*^{R806H}::^{P}*FRH:FRH*^{R806H}:*GFP*:^{P}*trpC:NAT*^{R}:*trpC*^{T}:*FRH*^{T})	This study
VGB699/ VGB700 (Δ*WC1*/Δ*FRQ*)	*WC1/FRQ* double-deletion strain (Δ*WC1*::^{P}*trpC:GEN*^{R}:*trpC*^{T}; Δ*FRQ*::^{P}*gpdA*:*HYG*^{R}:*trpC*^{T})	This study
VGB701/ VGB702 (Δ*WC1*/ *FRH*^{R806H})	*WC1* deletion and *FRH*^{R806H} point mutation double mutant strain (Δ*WC1*::^{P}*trpC:GEN*^{R}:*trpC*^{T}; *FRH*^{R806H}::^{P}*FRH:FRH*^{R806H}:^{P}*trpC:HYG*^{R}:*trpC*^{T}:*FRH*^{T})	This study

Δ: deletion; C: complementation; eC: ectopic complementation; OE: overexpression; ^{P}: promoter; ^{R}: resistance marker; ^{T}: terminator; VGB: *Verticillium* strain collection Gerhard H. Braus; two VGB numbers for one strain represent two independent transformants

Table S4. Oligonucleotides for qRT-PCR.

Primer name	Sequence (5' to 3')	Target gene	Reference
AN98	GGC AGA TGG AGA AGG TGA ATC	*WC1*/ *VDAG_JR2_Chr2g01990a*	This study
AN99	GCG CAG TCT CGG ACC ACA TT	*WC1*/ *VDAG_JR2_Chr2g01990a*	This study
AN112	GTA CGG ATT GTG GCA CGC TC	*WC2*/ *VDAG_JR2_Chr7g03830a*	This study
AN113	CTC GGG CTG TTC TTG ACC TTC	*WC2*/ *VDAG_JR2_Chr7g03830a*	This study
AN121	CCG GAC TCT TTT GCT GGT TG	*SFL1*/ *VDAG_JR2_Chr4g02790a*	This study
AN122	GTC GGT GGG GTT CAG TAT GTG	*SFL1*/ *VDAG_JR2_Chr4g02790a*	This study
AN123	GCG AAG AGA CCT CGT CAT C	*ABA1*/ *VDAG_JR2_Chr1g03550a*	This study
AN124	GGT TGA CGC AGA CAT TTT GCT G	*ABA1*/ *VDAG_JR2_Chr1g03550a*	This study
ML118	ATG CAC CAT CAG GCT TCT ATC	*FRQ*/ *VDAG_JR2_Chr1g01960a*	This study
ML119	ATG AGT TGG CCT AGG CTC	*FRQ*/ *VDAG_JR2_Chr1g01960a*	This study
q*SOM1*-F	CCA ACA AGC AGA TGC CTA ATG C	*SOM1*/ *VDAG_JR2_Chr1g09120a*	[61]
q*SOM1*-R	CTT GCA GAG CGT GGT TAC TTC C	*SOM1*/ *VDAG_JR2_Chr1g09120a*	[61]
q*VTA2*-F	TAC TCC TTC GTT CCG ATT CCT G	*VTA2*/ *VDAG_JR2_Chr5g09630a*	[67]
q*VTA2*-R	GCG CAT TGA GAT GGT TCA GAG T	*VTA2*/ *VDAG_JR2_Chr5g09630a*	[67]
q*VTA3*-F	GGA TGG CAA AGT CAA CGT CT	*VTA3*/ *VDAG_JR2_Chr1g07600a*	[61]
q*VTA3*-R	CGA ACA GAC CGA ATT GAT CC	*VTA3*/ *VDAG_JR2_Chr1g07600a*	[61]
SB34	GGC GAG TCT AGG TAT CAG TCT	*VTA1*/ *VDAG_JR2_Chr1g15920a*	[68]
SB35	CAC ATC TCG GAA CTG AGC GTA	*VTA1*/ *VDAG_JR2_Chr1g15920a*	[68]
SB40	ACC ATA GTT TGG TGG ATG CAA G	*CMR1*/ *VDAG_JR2_Chr1g15930a*	[68]
SB41	CGA CGG GTC ACC ATG ATG	*CMR1*/ *VDAG_JR2_Chr1g15930a*	[68]
SZ9	AAC ACC CAG AAC AAG ATG CGC	*H2A*/ *VDAG_JR2_Chr4g01430a*	[69]
SZ10	GCT TGA CCT TGA GAT CCT TG	*H2A*/ *VDAG_JR2_Chr4g01430a*	[69]

**Table S4.** Oligonucleotides for qRT-PCR, continued.			
**Primer name**	**Sequence (5’ to 3’)**	**Target gene**	**Reference**
SZ11	TGC ATT CTT GGC AAG AGA TGT GTG	*EIF2Bl VDAG_JR2_Chr4g00410a*	[68]
SZ12	AGC TTG TTA TCC TTG TCC TCG GT	*EIF2Bl VDAG_JR2_Chr4g00410a*	[68]

Table S5. Significantly enriched proteins with LFQ intensities, MS/MS counts, sequence coverage, and unique peptides in all three replicates of Frh–GFP in comparison with the wild-type control. Listed are proteins that were significantly enriched in all four repetitions of the “Replace missing values from normal distribution“ command.

LFQ intensity						MS/MS count						Sequence coverage [%]						Unique peptides						Protein IDs
WT			Frh			WT			Frh			WT			Frh			WT			Frh			
1	2	3	1	2	3	1	2	3	1	2	3	1	2	3	1	2	3	1	2	3	1	2	3	
NaN	NaN	NaN	27.5254	28.4655	28.6644	0	0	0	7	10	13	0	0	0	8.9	13.9	15.2	0	0	0	7	11	13	VDAG_JR2_Chr1g01960a-00001 (Frq)
27.7727	27.6803	27.6232	28.7411	28.626	28.5515	11	7	5	9	11	10	34.7	17.2	19	26.7	34.7	27.4	10	4	5	9	10	9	VDAG_JR2_Chr1g22320a-00001
24.7941	NaN	NaN	26.0249	25.9681	26.4116	2	0	0	2	3	2	12.6	0	0	18.5	31.1	12.6	2	0	0	2	3	2	VDAG_JR2_Chr3g06170a-00001
NaN	NaN	NaN	33.6518	36.2984	35.3208	0	0	0	86	193	139	0	0	0	41.3	61.6	50.7	0	0	0	48	75	59	VDAG_JR2_Chr4g00070aa-00001 (Frh)
26.187	25.9518	26.2343	27.4336	26.91	27.3289	5	2	3	5	9	6	11.6	3.3	7.2	15.4	25.8	14	5	2	3	5	9	6	VDAG_JR2_Chr5g06120a-00001
28.6871	28.7241	29.5188	31.2076	32.24	30.5252	9	7	10	13	15	9	38.4	18.5	41.1	42.4	57	45.7	7	3	7	8	9	7	VDAG_JR2_Chr8g05380a-00001
NaN	NaN	NaN	29.6374	30.8504	30.6564	2	0	1	2	3	2	17	0	9.4	25.5	24.5	17.9	2	0	1	3	3	2	VDAG_JR2_Chr8g06220a-00001

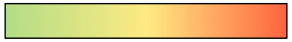

24.79 36.30
Log2(x)LFQ intensity

Table S6. Predicted protein domains or family types of proteins significantly enriched with Frh–GFP. Listed are proteins that were significantly enriched in all four repetitions of the "Replace missing values from normal distribution" command. Protein domains or family types were predicted using InterPro [79].

Protein IDs	Predicted domains or family types
VDAG_JR2_Chr1g01960a-00001 (Frq)	No predicted domain;<br>Family type: Frequency clock protein (IPR018554)
VDAG_JR2_Chr1g22320a-00001	No predicted domain;<br>Homologous superfamily type: Acyl-CoA N-acyltransferase (IPR016181)
VDAG_JR2_Chr3g06170a-00001	No predicted domain;<br>Family type: Protein of unknown function DUF3602 (IPR022024)
VDAG_JR2_Chr4g00070aa-00001 (Frh)	Helicase superfamily 1/2, ATP-binding domain (IPR014001);<br>DEAD/DEAH box helicase domain (IPR011545);<br>Helicase, C-terminal (IPR001650);<br>rRNA-processing arch domain (IPR025696);<br>ATP-dependent RNA helicase Ski2, C-terminal (IPR012961)
VDAG_JR2_Chr5g06120a-00001	RNA recognition motif domain (IPR000504)
VDAG_JR2_Chr8g05380a-00001	Ribosomal protein S13/S15, N-terminal (IPR012606)
VDAG_JR2_Chr8g06220a-00001	No predicted domain;<br>Family type: Ribosomal protein L36e (IPR000509)

Table S7. Significantly enriched protein with LFQ intensities, MS/MS counts, sequence coverage, and unique peptides in all three replicates of Frh^{R806H}–GFP in comparison with the wild-type control. Listed is the protein that was significantly enriched in all four repetitions of the "Replace missing values from normal distribution" command.

LFQ intensity						MS/MS count						Sequence coverage [%]						Unique peptides						Protein IDs
WT			Frh^{R806H}			WT			Frh^{R806H}			WT			Frh^{R806H}			WT			Frh^{R806H}			
1	2	3	1	2	3	1	2	3	1	2	3	1	2	3	1	2	3	1	2	3	1	2	3	
NaN	NaN	NaN	34.984	35.4083	34.2067	0	0	0	110	82	104	0	0	0	49.6	32.5	52.1	0	0	0	58	36	59	VDAG_JR2_Chr4g00070aa-00001 (Frh)

24.79 36.30
Log2(x)LFQ intensity
